# Universal Non-Extensive Statistical Physics Temporal Pattern of Major Subduction Zone Aftershock Sequences

**DOI:** 10.3390/e24121850

**Published:** 2022-12-19

**Authors:** Eleni-Apostolia Anyfadi, Sophia-Ekaterini Avgerinou, Georgios Michas, Filippos Vallianatos

**Affiliations:** 1 Section of Geophysics-Geothermics, Department of Geology and Geoenvironment, National and Kapodistrian University of Athens, 15784 Athens, Greece; 2 Institute of Physics of Earth’s Interior and Geohazards, UNESCO Chair on Solid Earth Physics and Geohazards Risk Reduction, Hellenic Mediterranean University Research & Innovation Center, 73133 Chania, Greece

**Keywords:** subduction zones, megathrust earthquakes, aftershock sequences, interevent times, superstatistics, Tsallis entropy

## Abstract

Large subduction-zone earthquakes generate long-lasting and wide-spread aftershock sequences. The physical and statistical patterns of these aftershock sequences are of considerable importance for better understanding earthquake dynamics and for seismic hazard assessments and earthquake risk mitigation. In this work, we analyzed the statistical properties of 42 aftershock sequences in terms of their temporal evolution. These aftershock sequences followed recent large subduction-zone earthquakes of M ≥ 7.0 with focal depths less than 70 km that have occurred worldwide since 1976. Their temporal properties were analyzed by investigating the probability distribution of the interevent times between successive aftershocks in terms of non-extensive statistical physics (NESP). We demonstrate the presence of a crossover behavior from power-law (*q* ≠ 1) to exponential (*q* = 1) scaling for greater interevent times. The estimated entropic *q*-values characterizing the observed distributions range from 1.67 to 1.83. The *q*-exponential behavior, along with the crossover behavior observed for greater interevent times, are further discussed in terms of superstatistics and in view of a stochastic mechanism with memory effects, which could generate the observed scaling patterns of the interevent time evolution in earthquake aftershock sequences.

## 1. Introduction

Megathrust faults, which bring together the surfaces of overthrusting and underthrusting plates in subduction zones, host the largest earthquakes on Earth. The downward motion of the subducting plate can generate exceptionally larger earthquakes with magnitudes even greater than M_w_ 7, releasing tremendous amounts of seismic energy that can have devastating repercussions across wide geographic regions. These large earthquakes generate prolonged aftershock sequences, which may last days to months or even years [1,2,3,4,5,6]. Not only can the mainshock of a megathrust earthquake cause extensive damages to many structures in its vicinity and the loss of lives, but the aftershocks that follow may also have destructive consequences. As a result, emergency preparedness and planning must include the effects of aftershocks on susceptible sectors of society and infrastructure [1,2,3,4]. This is achieved by estimating the parameters of various well-established empirical laws for aftershock sequences following major mainshocks in a specific seismogenic region [6,7,8]. In a subduction zone, the intense friction between the descending and overriding plates may generate shallow, intermediate, or deep earthquakes, which may occur both within the descending and overriding plates as well as along the interface between the two plates [9,10]. The magnitudes of such earthquakes vary greatly, depending on the sorts of boundaries that cause them. Almost all major earthquakes occur along boundaries of convergence or transform. Megathrust earthquakes are the most devastating events, with magnitudes reaching or exceeding M_w_ 9.0 in some cases. As a result, a deeper understanding is crucial for earthquake physics and earthquake early warning systems. Aftershock sequences following megathrust earthquakes can raise the level of seismic risk, even in distant areas far away from the mainshock’s fault zone.

To this aim, the aftershock sequences of significant subduction-zone earthquakes with magnitudes greater than 7.0 that occurred between 1976 and 2020 were investigated, with a focus on their temporal distributions in view of the ideas of statistical physics. The statistical physics approach expresses the basic principles of the evolution of seismicity using the universal principle of entropy. In particular, we used the non-extensive statistical physics (NESP) framework, which provides a generalization of the ordinary Boltzmann–Gibbs (BG) statistical physics [11,12,13,14,15,16,17]. The main advantage of using NESP is that all length correlations and memory effects are considered in the temporal evolution of seismicity, including BG statistical physics as a particular case [18,19,20,21,22]. In this work, the temporal scaling characteristics of major subduction zone aftershock sequences in terms of NESP were studied by estimating the probability distribution of the interevent times between successive aftershocks and its non-additive entropic parameter (*q*) [12,18,23,24,25,26,27,28,29]. We show that the observed probability distributions present a universal behavior with a crossover from power-law (*q* ≠ 1) to exponential (*q* = 1) scaling at longer interevent times.

## 2. Materials and Methods

Non-extensive statistical physics (NESP), based on the generalization of BG statistics, is an appropriate method to describe complex systems with (multi)fractality, long-range interactions, and long-term memory effects, resulting in power-law asymptotic behavior that is widely observed in nature [12,30,31,32,33,34,35,36,37,38,39,40]. Central to NESP is the expression of Tsallis entropy (*S_q_*) [12,31,32,33], which is given as

(1) $$S_{q}=k_{B}\frac{1-\sum p^{q}\left(X\right)}{q-1},\;$$

in terms of a fundamental parameter’s probability distribution (*p*(*X*)). In the present work, *X* is the interevent time (*T*), i.e., the time interval between successive aftershock events [35,41], and *k_B_* is Boltzmann’s constant. The *q* parameter is the so-called entropic index, which signifies the degree of non-additivity in the system [12]. For the particular case of *q* = 1, $S_{q}=S_{BG}$ and the BG entropy is recovered. Despite the fact that $S_{q}$ and $S_{BG}$ have many similarities, such as non-negativity, expansibility, and concavity, there is a major difference between the two formalisms. The BG entropy is additive, meaning that the entropy of a coupled system is equal to the sum of the entropies of its constituent components, whereas the Tsallis entropy ($S_{q}$) (with *q* ≠ 1) is non-additive [12,18,30,42,43]. Tsallis entropy satisfies the following condition for any two probabilistically independent systems, *A* and *B*:

(2) $$S_{q}\left(A+B\right)=S_{q}\left(A\right)+S_{q}\left(B\right)+\frac{\left(q-1\right)}{k_{B}}S_{q}\left(A\right)S_{q}\left(B\right),\;$$

In particular, *q* < 1 corresponds to superadditivity, and *q* > 1 corresponds to subadditivity, while the right-hand side of Equation (2) disappears when *q* = 1, so it corresponds to the additivity characteristics [12,24,44,45,46,47,48,49,50,51,52,53,54,55,56].

If *X* is a physical parameter that characterizes the system, such as earthquake interevent times (*T*), the probability distribution (*p*(*T*)) is determined using the Lagrange multipliers method by maximizing the entropy under suitable constraints [12]. Using the previous approach, Equation (1) leads to the probability distribution function

(3) $$p\left(T\right)=\frac{{\left[1-\left(1-q\right)\left({\frac{T}{T}}_{q}\right)\right]}^{\frac{1}{1-q}}}{Z_{q}}=\frac{exp_{q}\left(-T/T_{q}\right)}{Z_{q}},\;$$

where *Z_q_* is the *q*-partition function

(4) $$Z_{q}=\int_{0}^{\infty }exp_{q}\left(-T/T_{q}\right)dT,\;$$

and *T_q_* is a generalized scaled interevent time. The nominator of Equation (3) is the *q*-exponential function, which is defined as [12]

(5) $$exp_{q}\left(X\right)={\left[1+\left(1-q\right)X\right]}^{\frac{1}{1-q}},\;$$

for $1+\left(1-q\right)X\geq 0$, while in other cases $exp_{q}\left(X\right)=0$.

The cumulative distribution function $P\left(>T\right)=N(>T)/N_{o}$, where *N*(>*T*) is the number of interevent times with values greater than *T* and *N_o_*, their total number, is estimated as [24]

(6) $$P\left(>T\right)=exp_{Q}\left(-T/T^{*}\right),\;$$

which has the mathematical form of a *q*-exponential function with $T^{*}=T_{q}Q$ and q=2−(1/Q). The inverse function of the Q-exponential function is the so-called Q-logarithmic function, which is defined as [24]

(7) $$ln_{Q}P(>T)=\frac{P{\left(>T\right)}^{1-Q}-1}{1-Q},\;$$

From Equation (7), it follows that $ln_{Q}P\left(>T\right)=-\frac{T}{T^{*}}$, indicating a straight line with slope −1/$T^{*}$.

## 3. Data Selection and Analysis

**Figure 1:**
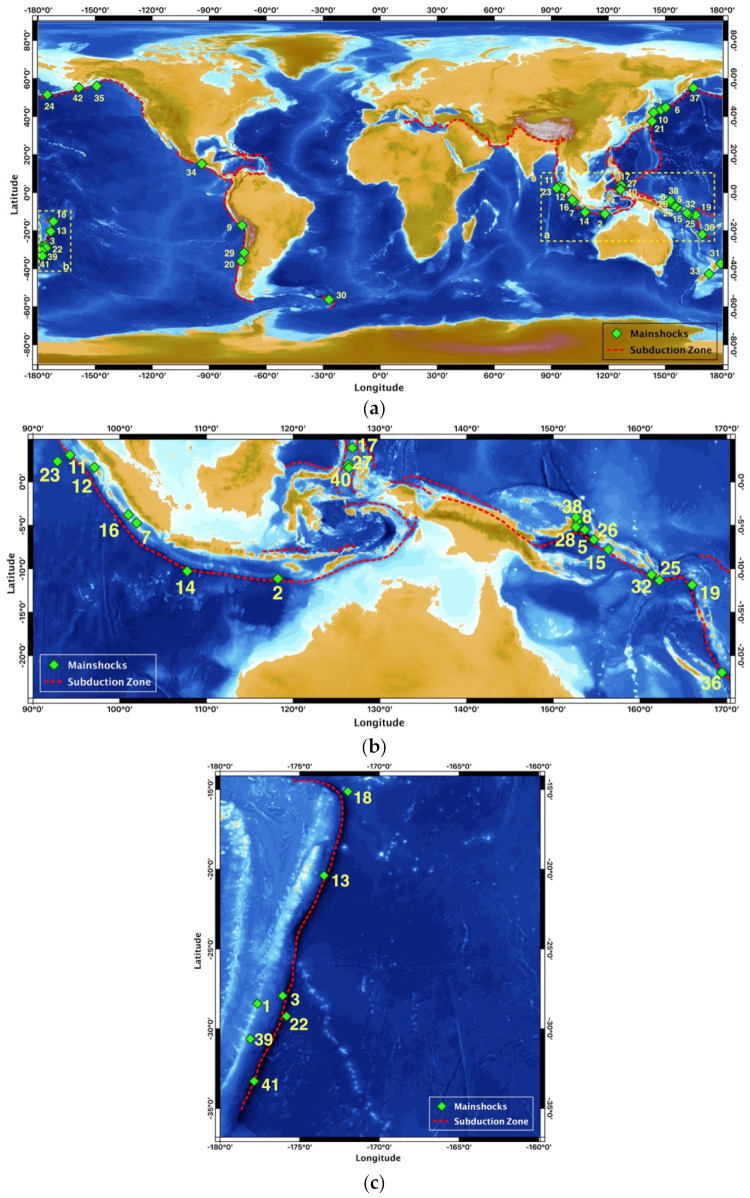
(**a**) Geographical distribution of the 42 subduction-zone earthquakes that were studied. The event indexes correspond to those listed in Table 1, while the dashed lines represent the subduction zones. The details of earthquakes located on the dashed squares are extracted in panels (**b**,**c**).

Herein, the statistical properties of the temporal evolution of aftershock sequences that followed large subduction-zone earthquakes worldwide during the last few decades are presented. The analyzed earthquakes occurred in various subduction zones all around the edge of the Pacific Ocean, Canada, Alaska, Russia, Indonesia, and Japan (Figure 1). We used 42 aftershock sequences generated by mainshocks of M_w_ 7.0 and greater with focal depths less than 70 km that occurred from January 1976 to July 2020 and were located at a maximum distance of 100 km from the main event. The aftershock catalogues were extracted from the United States Geological Survey (USGS) database [48]. To create the catalogues of the aftershock sequences, an elliptical region with a maximum distance of 100 km from the mainshock was selected for each main event, based on the distribution of aftershocks, to designate a probable aftershock zone. Then, all earthquake events that occurred within this region, for a maximum period of two years after the mainshock, were included in the catalogue. Once the catalogues were defined, the frequency–magnitude distribution was used to estimate the completeness magnitude (M*_c_*) of each aftershock sequence [49,50]. The catalogues that were analyzed consisted of at least 100 events. Ultimately, the final list included 42 mainshocks and their aftershock sequences (see Table 1).

Figure 1 depicts the geographical locations of the 42 studied mainshocks that occurred in subduction zones around the world. The event indexes correspond to the number of each mainshock in Table 1, which are listed chronologically from the oldest to the most recent. The parameters of each mainshock and its aftershock sequence, along with the entropic parameter (*q*), were calculated for the interevent time distribution and are presented in Table 1, along with the parameter *T_c_*, which marks the crossover points between the non-additive and additive behavior in each aftershock sequence.

**Figure 2:**
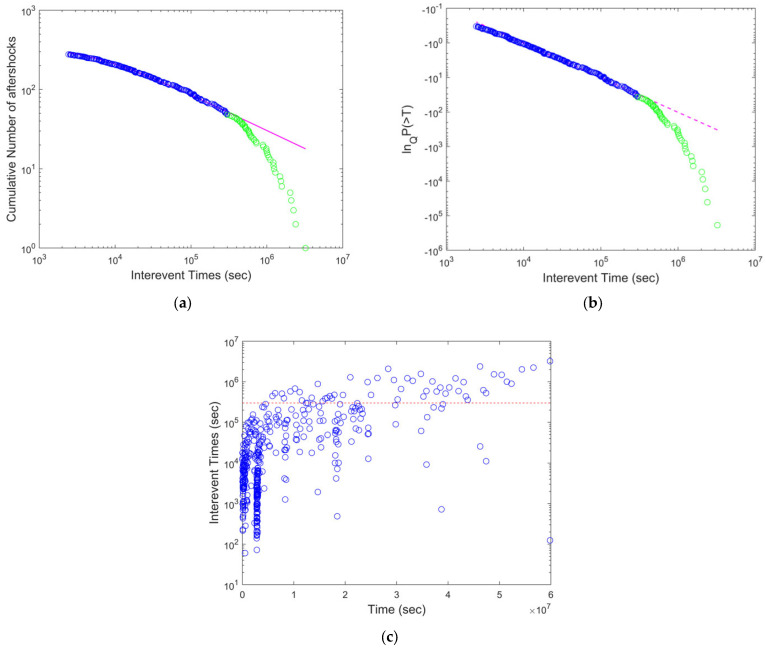
(**a**) The cumulative distribution function of the interevent times for the *2004 M_w_ 9.0 Sumatra–Andaman Islands Earthquake.* The magenta line is the Q-exponential function fitting with *q* = 1.69. (**b**) The Q-logarithmic function of *P*(>*T*) as a function of the interevent times (*T*), where the dashed line is the Q-exponential function fitting with *q* = 1.69, exhibiting a correlation coefficient of 0.9923. The deviation from linearity suggests a *T_c_* value close to 3 × 10^5^ s. (**c**) The evolution of interevent times (*T*) as a function of the time (*t*) since the main event. The *T_c_* value is indicated by the red dashed line.

For each aftershock sequence, the cumulative interevent time distribution was estimated, and the corresponding fitting with the Q-exponential function, up to the value *T_c_*, provided the Q and *q* parameter values. We noted that, for large values of *T*, with *Τ* > *T_c_*, a deviation from the Q-exponential function was observed (e.g., Figure 2a). In addition, the Q-logarithmic function of *P*(>T) as a function of the interevent times (*T*) was plotted using the Q value estimated in the previous analysis. Then, the range of interevent times where ln_Q_*P*(>*T*) vs. *T* was a straight line, given by Equation (8), was defined with its correlation coefficient. The deviation from the linearity at *T_c_* indicated the crossover between NESP and BG statistical physics. Furthermore, the evolution of the interevent times (*T*) as a function of time (*t*) since the main event further indicated that, in short time scales after the mainshock, the main driving mechanism is governed by NESP, while, as the aftershock sequence evolves at *T* > *T_c_*, the system is governed by BG statistical mechanics. The aforementioned earthquake statistical analysis and processing was carried out for all major earthquake aftershock sequences listed in Table 1, with results consistent with the previous analysis for all aftershock sequences, indicating a universal behavior in their temporal evolution.

In Table 1, the results of the analysis of the 42 subduction zone aftershock sequences are summarized. For each aftershock sequence, the entropic index (*q*) of the interevent time distribution, along with the generalized scaled interevent time (*T_q_*) and the critical interevent time (*T_c_*), are presented. In the following section, we present some characteristic cases referring to the strongest events during the last few decades: the 2004 M_w_ 9.0 Sumatra–Andaman Islands Earthquake and the 2011 M_w_ 9.1 Great Tohoku (Japan) Earthquake. The corresponding results for the other aftershock sequences listed in Table 1 are provided in the Supplementary Materials. 

### 3.1. The 2004 M_w_ 9.0 Sumatra–Andaman Islands Earthquake

The M_w_ 9.0 Sumatra earthquake, which occurred on 26 December 2004 at a focal depth of 30 km, was the fourth largest earthquake recorded since 1900. It originated from thrust faulting between the meeting point of the Indian plate and the Burma micro-plate. According to the USGS database, 356 aftershocks occurred with magnitudes greater than M_w_ 5.1 in a period of two years after the mainshock. One of the greatest disasters recorded in human history was brought on by the tsunami generated by the mainshock. More than 283,000 people were killed in total, while the severeness and impact of the earthquake were demonstrated by the fact that the tsunami crossed the Pacific and Atlantic Oceans and was recorded in New Zealand as well as along the west and east coasts of South and North America. Tsunamis continued to occur in Mozambique, South Africa, Australia, and Antarctica. The mainshock even caused eruptions in a mud volcano at Baratang, Andaman Islands, on 28 December 2004 [51,52].

The cumulative distribution function of the interevent times for the 2004 M_w_ 9.0 Sumatra–Andaman Islands Earthquake was fitted for *T* < *T_c_* with the Q-exponential function for *q* = 1.69 (Figure 2). The corresponding Q-logarithmic function of *P*(>*T*), as a function of the interevent times for *q* = 1.69, was fitted by a straight line, as given by Equation (8), with a correlation coefficient of 0.9923. The deviation from linearity was observed at a *T_c_* value close to 3 × 10^5^ s, indicating the crossover point between NESP and BG statistical physics. Furthermore, the evolution of the interevent times (*T*) as a function of the time (*t*) since the main event is presented in Figure 2c. The *T_c_* value (red dashed line in Figure 2c) indicates that the majority of interevent times in the early period following the mainshock had *T* values less than *T_c_*, suggesting that the Tsallis entropic mechanism was predominant in the immediate part of the aftershocks’ evolution. As time evolved, some of the characteristics of the early aftershock sequence, such as that of the long-range memory related to NESP, were less predominant and the BG statistical physics recovered (i.e., *q* = 1).

### 3.2. The 2011 M_w_ 9.1 Great Tohoku (Japan) Earthquake

The M_w_ 9.1 Tohoku earthquake, which occurred on 11 March 2011, was generated by thrust faulting at the boundary of the subduction zone between the Pacific and North American plates. It was located close to Honshu, on Japan’s northeast coast. This earthquake was preceded by many strong foreshocks that occurred over a period of two days prior to the mainshock, starting on March 9 with an M_w_ 7.2 earthquake and continuing the same day with three more earthquakes greater than M_w_ 6.0. Additionally, during the period of two years after the mainshock, 435 aftershocks of magnitudes equal or greater than M_w_ 5.0 occurred. The mainshock generated a tsunami reaching heights up to 40.5 m, with a devastating impact on Japan’s northern island coastal areas, before spreading all over the Pacific coasts of North and South America, from Alaska to Chile. The event’s destructive tsunami waves are estimated to have contributed to 19,759 fatalities and 6242 injuries. The Fukushima nuclear power plant was damaged as a result of the tsunami, which led to significant radioactive pollution [52,53].

**Figure 3:**
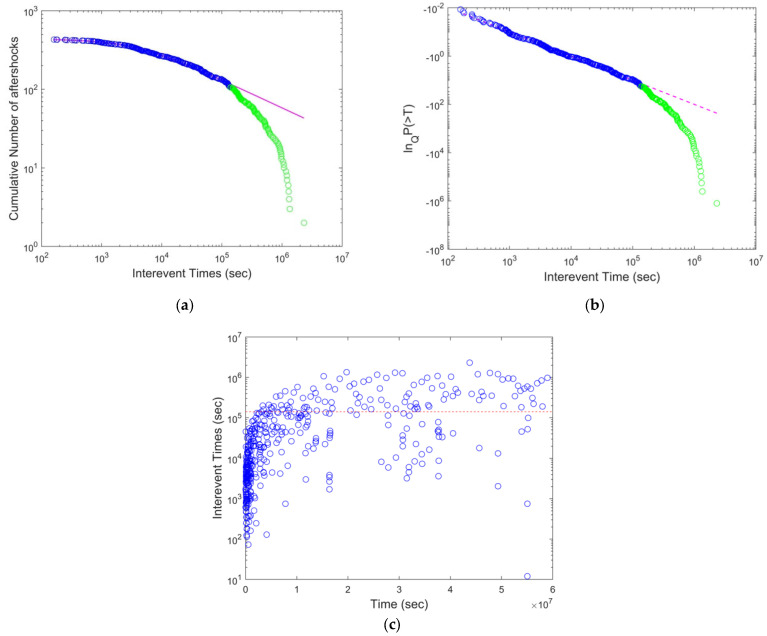
(**a**) The cumulative distribution function of the interevent times (*T*) for the *2011 Mw 9.1 Great Tohoku* (*Japan*) *Earthquake*. The magenta line is the Q-exponential function fitting with *q* = 1.74. (**b**) The Q-logarithmic function of *P*(>*T*) as a function of the interevent times (*T*). The dashed line is the Q-exponential function fitting with *q* = 1.74, presenting the correlation coefficient of 0.9828. The deviation from linearity suggests a *T_c_* value close to 1 × 10^5^ s. (**c**) The evolution of the interevent times (*T*) as a function of the time (*t*) since the main event. The *T_c_* value is indicated by the red dashed line.

The cumulative distribution function of the interevent times, presented in Figure 3a, presented similar characteristics as the previous case of the M_w_ 9.0 Sumatra earthquake. For *T* < *T_c_*, it was fitted by a Q-exponential function with *q* = 1.74 (Figure 3a), while the corresponding Q-logarithmic function of *P*(>*T*) presented a correlation coefficient of 0.9828 for *T* values up to *T_c_* (Figure 3b). The deviation from linearity was observed at a *T_c_* value close to 1 × 10^5^ s, indicating the crossover point between NESP and BG statistical physics (Figure 3c).

**Figure 4:**
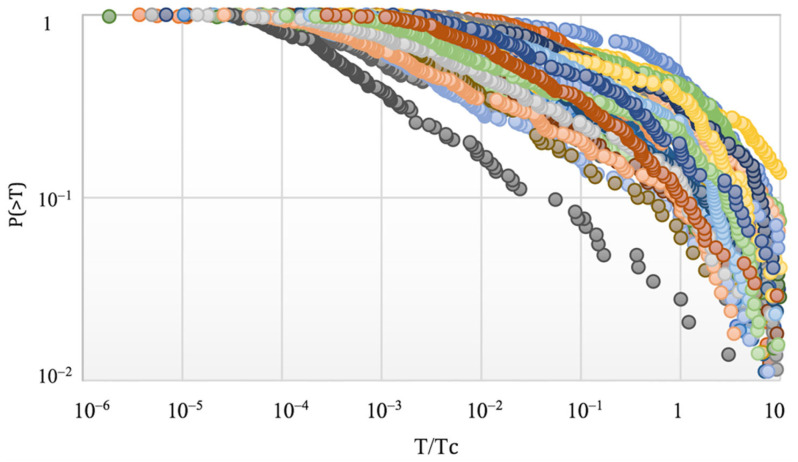
The cumulative interevent time distributions for all analyzed aftershock sequences as functions of *T*/*T_c_*. For all sequences, a deviation from the Q-exponential function is presented for *T*/*T_c_* > 1.

Since the results have demonstrated that *P*(>*T*) = exp_Q_ (−*T*/T*) for *T* < *T_c_*, we introduced a new variable, *x* = *T*/*T_c_*, for which *x* < 1 suggests the range where the Tsallis entropic mechanism is predominant, while *x* > 1 is related to the exponential roll-off in the tail of the distribution. In Figure 4, the cumulative interevent time distributions are presented for all aftershock sequences listed in Table 1. For all analyzed aftershock sequences, a deviation from the Q-exponential function existed for *x* > 1 (i.e., *T* > *T_c_*). It is straightforward that *P*(>*x*) = exp_Q_(−*x*/x*), where x* = T*/*T_c_*. An inspection of Figure 4 suggested that for 0.01 < *x* < 1 a power-law scaling range was observed for all aftershock sequences, with an average slope of 0.33, which corresponds to a value of *q* ≈ 1.75, in general agreement with the range of *q* values reported in Table 1. 

## 4. Discussion

In the present work, the temporal patterns of major subduction zone aftershock sequences that occurred from 1976 to 2020 were analyzed in terms of non-extensive statistical physics. We observed that in all cases a Q-exponential function described the cumulative distribution *P*(>*T*) of the aftershock interevent times for short timescales, while for large values of *T* (*T* > *T_c_*), where *T*_c_ was a critical crossover interevent time between the non-additive and additive behavior, a deviation from the Q-exponential function appeared. For each aftershock sequence, the entropic parameter (*q*) was estimated by fitting a Q-exponential function to the observed data up to a value near *T_c_*. Thus, the applicability of non-extensive statistical physics to the cumulative distribution functions of interevent times and the presence of a crossover behavior from power-law (*q* ≠ 1) to exponential (*q* = 1) scaling for greater interevent times was demonstrated. The latter implies a sub-additive process with *q*-values greater than one, supporting the concept of long-range memory in the temporal evolution of aftershocks for *T* < *T_c_*. Furthermore, most of the estimated non-extensive *q*-values that characterized the observed distributions were within the range of 1.67–1.83. 

The observed deviation from the Q-exponential function for longer interevent times can be described as the superposition of two aftershock mechanisms. The first mechanism, described by Tsallis entropy, was dominant for interevent times with *T* < *T_c_*, whereas the second, characterized by an exponential function, became evident for *T* > *T_c_*. To incorporate a crossover from anomalous (*q* ≠ 1) to normal BG (*q* = 1) statistical physics, we introduced the generalization presented in [54,55] where

(8) $$\frac{dp\left(T\right)}{dT}=-\beta_{1}p-\left(\beta_{q}-\beta_{1}\right)p^{q},\;$$

whose solution is

(9) $$p\left(T\right)=C{\left[1-\frac{\beta_{q}}{\beta_{1}}+\frac{\beta_{q}}{\beta_{1}}e^{\left(q-1\right)\beta_{1}Τ}\right]}^{1/1-q},\;$$

where *C* is a normalization factor and *p*(*T*) decreases monotonically with increasing *T* for positive *β_q_* and *β*_1_. As a result, in the case where (*q* − 1)*β*_1_ ≪ 1, Equation (9) is approximated with a *q*-exponential, $p\left(T\right)\approx Cexp_{q}\left(-T/T_{q}\right)$, where $T_{q}=1/\beta_{q}$, whereas for (*q* − 1)*β*_1_ ≫ 1, the asymptotic behavior of the probability distribution $p\left(T\right)\propto {\left(\frac{\beta_{1}}{\beta_{q}}\right)}^{1/\left(q-1\right)}e^{-\beta_{1}T}$ is an exponential function, where $T_{c}=1/\left(q-1\right)\beta_{1}$ is the crossover point between the non-additive and additive behavior [11,32].

The *q*-exponential scaling behavior of interevent times for *T* < *T_c_* can be originated from a simple mechanism, namely a gamma-distributed allocated parameter (*β*) of the local Poisson process, and may be used to explain the interevent time distribution in aftershock sequences. The *T_c_* value indicated that in the early aftershock period the majority of interevent times had *T* values lower than *T_c_* and their distributions were described by NESP, while properties such as long-range memory, associated with NESP, became less prominent as the system relaxed and the BG statistical physics recovered [56,57,58,59,60,61].

The *q*-exponential behavior of the interevent times can further be viewed in terms of superstatistics, which are based on a superposition of ordinary local equilibrium statistical mechanics with a suitable intensive parameter (*β*) that varies as a gamma distribution on a reasonably wide temporal scale and is supplementary to NESP [19,23,61,62,63,64,65].

Then, a superstatistical approach for the interevent times of the earthquake aftershock sequences can be used, where the local Poisson process $p\left(T|\beta \right)=\beta e^{-\beta Τ}$ with *β* as an intensive fluctuating parameter has a particular value denoted by the equation $p\left(T|\beta \right)$. On a long time scale, this parameter is distributed with the probability density (*f*(*β*)) [19,62,63,64,65,66]. Then, the probability distribution (*p*(*T*)) is given as:

(10) $$p\left(T\right)=\int_{0}^{\infty }f\left(\beta \right)\beta e^{-\beta Τ}d\beta ,\;$$

In the case where the probability density of β is given by a gamma distribution:

(11) $$f\left(\beta \right)=\frac{1}{\Gamma \left(n/2\right)}{\left(\frac{n}{2\beta_{0}}\right)}^{n/2}\beta^{\frac{n}{2}-1}exp\left(-\frac{n\beta }{2\beta_{0}}\right),\;$$

the integral (10) can easily be evaluated [67] and $p\left(T\right)\approx C{\left(1+B\left(q-1\right)T\right)}^{1/\left(1-q\right)}$ is obtained, which is exactly the result estimated in the frame of NESP, with $q=1+\left(\frac{2}{n+2}\right)$ and $B=2\beta_{0}/\left(2-q\right)$ [23]. Since the *q* value was in the range of 1.67–1.81, it suggested that the system was derived by a low number of degrees of freedom, possibly close to one. 

This implied that a stochastic mechanism with memory effects can be the driving mechanism in the temporal evolution of an aftershock sequence. In agreement with [68] (see also [59]), we may consider the following stochastic differential equation for the evolution of seismicity:

(12) $$dT=-\gamma \left(Τ-\langle Τ\rangle \right)dt+\varphi \sqrt{Τ\;}W_{t},\;$$

where the temporal occurrence of earthquakes is represented by the interevent time series (*T*) after some time (*t*). The latter stochastic equation manifests two parts controlling the evolution of seismicity. The first deterministic part aims to keep the seismic rate (*R*) stable to the typical value of *R* = 1/<*T*>, according to a restoring constant (*γ*) that represents the rate of relaxation to the mean waiting time (<*Τ*>). The second stochastic part represents memory effects in the evolution of seismicity. The stochastic term *W_t_* is the standard Wiener process following a Gaussian distribution with a zero mean and unitary variance that mimics the microscopic effects in the evolution of interevent times in the aftershock sequence. Due to its random sign, *W_t_* leads to an increase (*W_t_* > 0) or decrease (*W_t_* < 0) in *Τ*. We note that large values of *T* provoke large amplitudes in the stochastic term, leading to an increase or decrease in *T,* depending on the sign of *W_t_*. The term *φ* adds some noise to the process and can be expressed as a function of the mean interevent time (<*T*>) and the restoring constant (*γ*) as $\varphi =\sqrt{2\gamma \langle Τ\rangle }$. 

The stochastic differential equation given in Equation (12) is a classic example of multiplicative noise, further known in statistics as the Feller process [67,69].

To determine the evolution of the interevent time series (*Τ*) after some time (*t*), given by the probability distribution *f*(*Τ*,*t*), we can write the corresponding Fokker–Planck equation for Equation (12) [70,71]:

(13) $$\frac{\partial f\left(T,t\right)}{\partial t}=\frac{\partial }{\partial T}\left[\gamma \left(Τ-\langle Τ\rangle \right)f\left(T,t\right)\right]+\frac{\partial^{2}}{\partial T^{2}}\left[T\langle T\rangle \gamma f\left(T,t\right)\right],\;$$

The stationary solution of the latter Fokker–Planck equation, Equation (13), is the distribution [70]:

(14) $$p(T/\langle T\rangle )=f\left(T\right)=\frac{1}{\langle T\rangle }e^{-\frac{\gamma }{\langle Τ\rangle }Τ},\;$$

In this case, Equation (14) provides the conditional probability of *Τ,* given <*Τ*>. 

Furthermore, we can consider local fluctuations in the seismic rate (*R* = 1/<*Τ*>), which are associated with non-stationarities in the evolution of the earthquake activity over time scales much larger than *γ*^−1^, which is necessary for Equation (12) to reach stationarity. In this case, local fluctuations in the mean interevent time (<*T*>) appear, and we may assume that these fluctuations follow the stationary gamma distribution:

(15) fT=(1λ)δΓδΤ−1+δe−1λΤ, 

The marginal probability of *T*, independent of <*T*>, is then given by [68]:

(16) $$p\left(T\right)=\int_{0}^{\infty }p\left(T/\langle T\rangle \right)f\left(\langle T\rangle \right)d\langle T\rangle ,\;$$

Performing the integration, we obtain the solution for varying <*T*>:

(17) $$p\left(T\right)=\frac{\lambda \Gamma \left[1+\delta \right]}{\Gamma \left[\delta \right]}{\left(1+\lambda Τ\right)}^{-\left(1+\delta \right)},\;$$

By further carrying out the changes in the variables:

(18) $$\lambda =\frac{q-1}{{Τ}_{ο}}\;\mathrm{and}\;\delta =\frac{1}{q-1}-1=\frac{2-q}{q-1}$$

and considering the *q*-exponential function given in Equation (3), Equation (12) can be written as [26,68]:

(19) pT=q−1Γ1q−1ToΓ1q−1−1expq−TTo                          

The last equation, Equation (19), is the *q*-generalized gamma function [68], and the last term on the right-hand side has the exact form of the *q*-exponential function given in Equation (3). Equation (19) was derived by the stochastic model, Equation (12), for a varying mean interevent time (<*T*>), i.e., non-stationary earthquake activity.

## 5. Conclusions

In order to statistically analyze the temporal patterns of aftershock sequences in major subduction zones, we examined the interevent time distribution for each sequence. The NESP approach to the interevent time distribution indicated a system in anomalous equilibrium, with a crossover behavior from anomalous (*q* > 1) to normal (*q* = 1) statistical physics for greater interevent times. The range of the non-extensive parameter (*q_T_*) for all analyzed sequences was between 1.67 and 1.83. The models used in the analysis fit the observed distributions rather well, indicating the usefulness of NESP in investigating such phenomena. Finally, the superstatistical approach led us to the conclusion that significant non-additive characteristics and a high-level organizational structure describe the earthquake aftershock sequences that occurred in subduction zones [55].

## Figures and Tables

**Table 1:** Summary of the results for the 42 subduction zone aftershock sequences. *M_c_* is the completeness magnitude of each catalogue, *N* is the number of aftershocks, *q* the entropic index of the interevent time distribution, *T_q_* is the generalized scaled interevent time, and *T_c_* is the critical interevent time, where a crossover from NESP to BG statistical mechanics appeared (see the text for details).

Index Number	Date	Epicenter (Lat, Lon)	Depth (km)	Duration (days)	Mainshock Magnitude (M_w_)	*M_c_*	*N*	*q_T_*	*T_q_* (s)	*T_c_* (s)
1	14 January 1976	−28.43, −177.66	33.00	346	8.0	5.4	101	1.78	4444	4 × 10^5^
2	19 August 1977	−11.14, 118.23	23.30	649	8.3	5.3	124	1.69	3750	5 × 10^5^
3	20 October 1986	−27.93, −176.07	50.40	732	7.7	5.3	103	1.74	1579	10 × 10^5^
4	4 October 1994	43.60, 147.63	68.20	248	8.3	5.2	219	1.72	472	2 × 10^5^
5	16 August 1995	−5.51, 153.64	45.60	89	7.7	5.2	100	1.77	2791	4 × 10^5^
6	3 December 1995	44.82, 150.17	25.90	333	7.9	5.2	138	1.83	200	2 × 10^5^
7	4 June 2000	−4.73, 101.94	43.90	719	7.9	5.2	162	1.82	1636	3 × 10^5^
8	16 November 2000	−4.56, 152.79	24.00	357	8.0	5.5	165	1.73	822	5 × 10^5^
9	23 June 2001	−17.28, −72.71	29.60	710	8.4	5.2	109	1.79	250	20 × 10^5^
10	25 September 2003	42.21, 143.84	28.20	708	8.3	5.1	110	1.78	4000	6 × 10^5^
11	26 December 2004	3.09, 94.26	28.60	692	9.0	5.1	356	1.69	3063	3 × 10^5^
12	28 March 2005	1.67, 97.07	25.80	709	8.6	5.1	210	1.83	667	2 × 10^5^
13	3 May 2006	−20.39, −173.47	67.80	713	8.0	5.0	100	1.75	22,500	3 × 10^5^
14	17 July 2006	−10.28, 107.78	20.00	186	7.7	5.3	133	1.67	433	0.2 × 10^5^
15	1 April 2007	−7.79, 156.34	14.10	687	8.1	5.2	115	1.81	2692	8 × 10^5^
16	12 September 2007	−3.78, 100.99	24.40	722	8.5	5.1	174	1.83	833	1 × 10^5^
17	11 February 2009	3.92, 126.81	23.90	699	7.2	5.2	126	1.81	346	20 × 10^5^
18	29 September 2009	−15.13, −171.97	12.00	728	8.1	5.2	228	1.83	1500	1 × 10^5^
19	7 October 2009	−11.86, 166.01	41.70	695	7.8	5.1	154	1.80	1600	4 × 10^5^
20	27 February 2010	−35.98, −73.15	23.20	720	8.8	5.0	190	1.76	6098	3 × 10^5^
21	11 March 2011	37.52, 143.05	20.20	717	9.1	5.0	435	1.74	26,316	1 × 10^5^
22	6 July 2011	−29.22, −175.83	32.50	364	7.6	5.1	140	1.71	6286	4 × 10^5^
23	11 April 2012	2.35, 92.82	45.60	359	8.6	5.2	129	1.80	286	8 × 10^5^
24	30 August 2013	51.54, −175.23	29.00	724	7.0	4.8	133	1.80	224	10 × 10^5^
25	12 April 2014	−11.35, 162.24	27.30	411	7.6	4.9	177	1.75	173	60 × 10^5^
26	19 April 2014	−6.64, 154.67	43.40	722	7.5	4.7	107	1.83	776	2 × 10^5^
27	15 November 2014	1.98, 126.37	45.00	664	7.1	4.6	119	1.81	472	3 × 10^5^
28	29 March 2015	−5.18, 152.59	37.60	719	7.5	4.7	245	1.71	2857	2 × 10^5^
29	16 September 2015	−31.57, −71.67	22.40	575	8.3	4.6	213	1.78	222	6 × 10^5^
30	28 May 2016	−56.24, −26.94	68.00	616	7.2	4.9	107	1.78	3333	3 × 10^5^
31	1 September 2016	−37.36, 179.15	19.00	580	7.0	4.7	166	1.74	263	3 × 10^5^
32	13 November 2016	−42.74, 173.05	15.10	648	7.8	4.9	144	1.72	556	50 × 10^5^
33	8 December 2016	−10.68, 161.33	40.00	479	7.8	5.0	100	1.72	278	10 × 10^5^
34	8 September 2017	15.02, −93.90	47.40	737	8.2	4.8	252	1.74	921	3 × 10^5^
35	23 January 2018	56.00, −149.17	14.10	437	7.9	4.5	113	1.81	367	2 × 10^5^
36	5 December 2018	−21.95, 169.43	10.00	670	7.5	4.8	166	1.78	217	1 × 10^5^
37	20 December 2018	55.10, 164.70	16.60	662	7.3	4.6	131	1.78	435	8 × 10^5^
38	14 May 2019	−4.05, 152.60	10.00	573	7.6	4.9	103	1.86	411	4 × 10^5^
39	15 June 2019	−30.64, −178.10	46.00	530	7.3	4.8	217	1.75	2250	3 × 10^5^
40	14 November 2019	1.62, 126.42	33.00	364	7.1	4.8	191	1.82	143	2 × 10^5^
41	18 June 2020	−33.29, −177.86	10.00	102	7.4	4.9	121	1.72	694	0.6 × 10^5^
42	22 July 2020	55.07, −158.60	28.00	138	7.8	4.2	207	1.69	313	0.5 × 10^5^

## Data Availability

Not applicable.

## Supplementary Materials

The following supporting information can be downloaded at: https://www.mdpi.com/article/10.3390/e24121850/s1.
