# Supplementary material for: Universal Non-Extensive Statistical Physics Temporal Pattern of Major Subduction Zone Aftershock Sequences
Source: Entropy (Basel). 2022 Dec 19;24(12):1850. doi: 10.3390/e24121850 (PMC9778025; doi:10.3390/e24121850)
Supplement: Supplementary file 1 [file entropy-24-01850-s001.zip › entropy-2045681-supplementary.pdf]

entropy MDPI

Supplementary Material

Universal Non-Extensive Statistical Physics Temporal Pattern of Major Subduction Zone Aftershock Sequences

Eleni-Apostolia Anyfadi 1,2, Sophia-Ekaterini Avgerinou 1,2, Georgios Michas 1,2 and Filippos Vallianatos 1,2,*

[1] Section of Geophysics-Geothermics, Department of Geology and Geoenvironment, National and Kapodistrian University of Athens, 15784 Athens, Greece
[2] Institute of Physics of Earth's Interior and Geohazards, UNESCO Chair on Solid Earth Physics and Geohazards Risk Reduction, Hellenic Mediterranean University Research & Innovation Center, Crete, 73133 Chania, Greece
* Correspondence: fvallian@geol.uoa.gr

Herein, we present the cumulative interevent time distributions for all aftershock sequences that were studied. Figures S1a-S42a present a typical Q-exponential (see Eq. 5) pattern in the log–log plot of the cumulative distribution function ($P(>T)$) of the aftershock interevent times and reveal that for large values of T ($T > T_c$), where T_c is a critical crossover interevent time, there is a deviation from the Q-exponential function. The entropic parameter (q) is obtained by fitting a Q-exponential function to the observed data up to a value near T_c. The Q-logarithm (see Eq. 7) implies that the expression $ln_Q P(> T) = -\frac{1}{T^*} T$ is linear with T, with a slope of $-1/T^*$ when the Q, which describes the distribution of the interevent time intervals, is introduced [48]. Figures S1b-S42b present $ln_Q P(> T)$ as a function of the interevent time (T), introducing the estimated q parameter, as presented in Table 1, while T_c is estimated from the linearity deviation. Figures S1c-S42c present the evolution of interevent time (T) as a function of the time (t) since the main event, indicating that at times close to the mainshock the main driving mechanism is governed by NESP, while as the aftershock sequence evolves to $T > T_c$, the system recovers BG statistical mechanics. Furthermore, the correlation coefficient for $ln_Q P(>T)$ with T, for $T < T_c$, is further provided.

1. The 1976 M_w 8.0 Kermadec Island Earthquake (New Zealand)

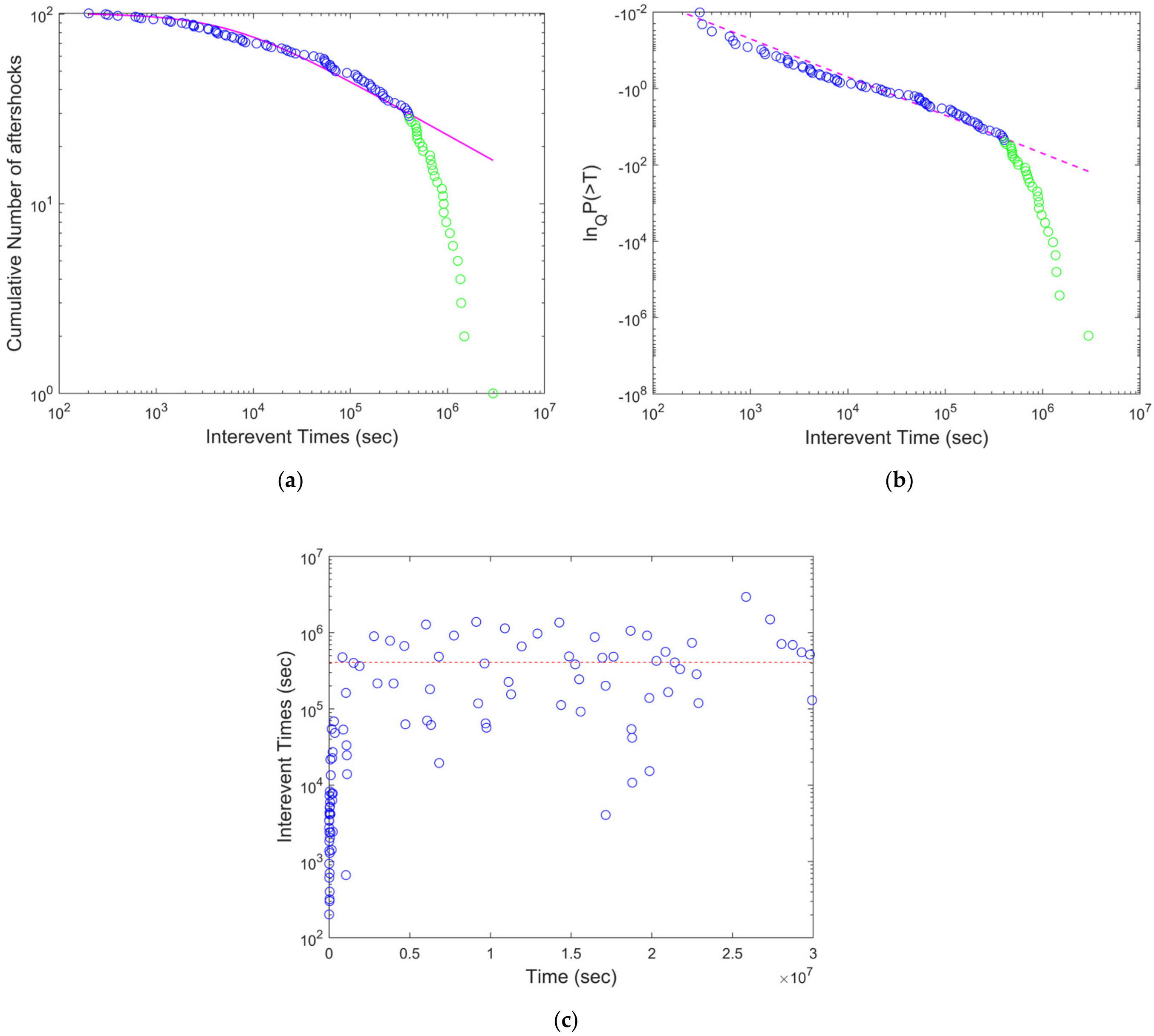

Figure S1. (**a**) The cumulative distribution function of the interevent times for the 1976 M_w 8.0 Kermadec Island Earthquake (New Zealand). The magenta line is the Q-exponential function fitting with $q = 1.78$. (**b**) The Q-logarithmic function of $P(>T)$ as a function of the interevent times, where the dashed line is the fitting with $q = 1.78$. The deviation from linearity suggests T_c values close to $4x10^5$ s. The correlation coefficient for the Q-logarithmic function up to T_c is $R^2 = 0.9827$. (**c**) The evolution of the interevent time (T) as a function of the time (t) since the main event. The T value is indicated by the red dashed line.

2. The 1977 M_w 8.3 Waingapu Earthquake (Indonesia)

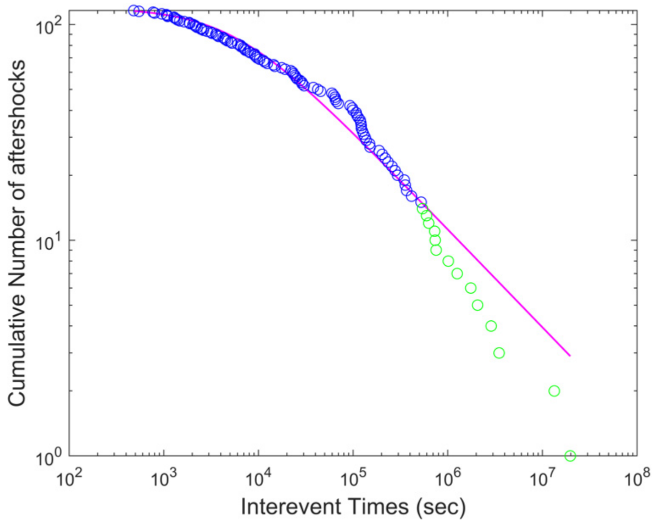

(**a**)

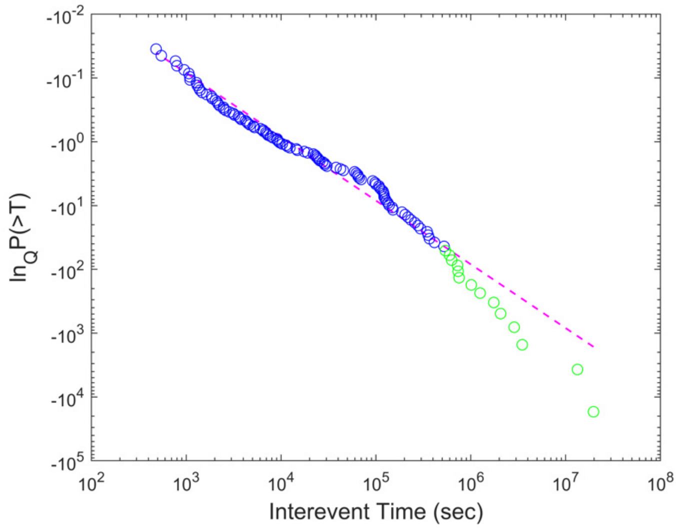

(**b**)

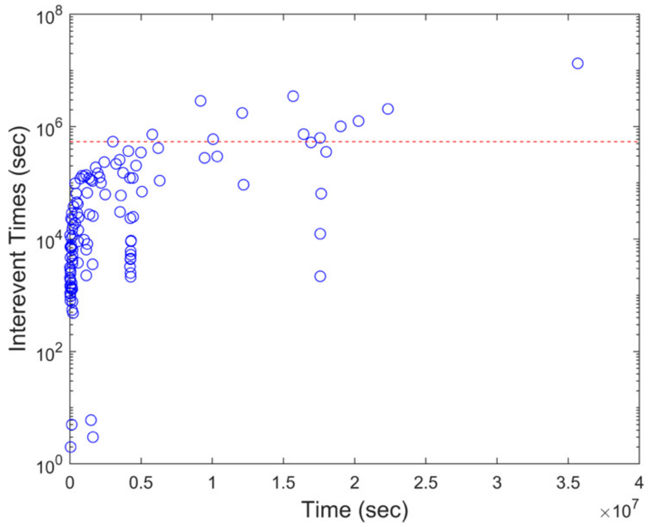

(**c**)

Figure S2. (**a**) The cumulative distribution function of the interevent times of the 1977 M_w 8.3 Waingapu Earthquake (Indonesia). The magenta line is the Q-exponential function fitting with q = 1.69. (**b**) The Q-logarithmic function of $P(>T)$ as a function of the interevent times, where the dashed line is the fitting with q = 1.69. The deviation from linearity suggests T_c values close to 5x10^{5}s. The correlation coefficient for the Q-logarithmic function up to T_c is R^2 = 0.9731. (**c**) The evolution of the interevent time (T) as a function of the time (t) since the main event. The T value is indicated by the red dashed line.

3. The 1986 M_w 7.7 Kermadec Island Earthquake (New Zealand)

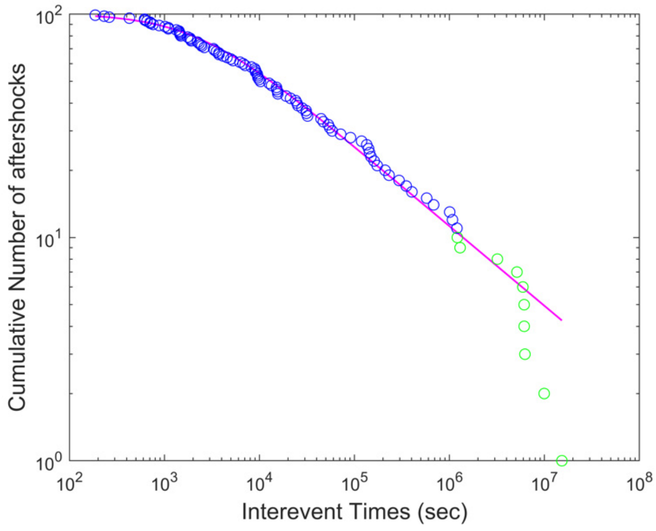

(**a**)

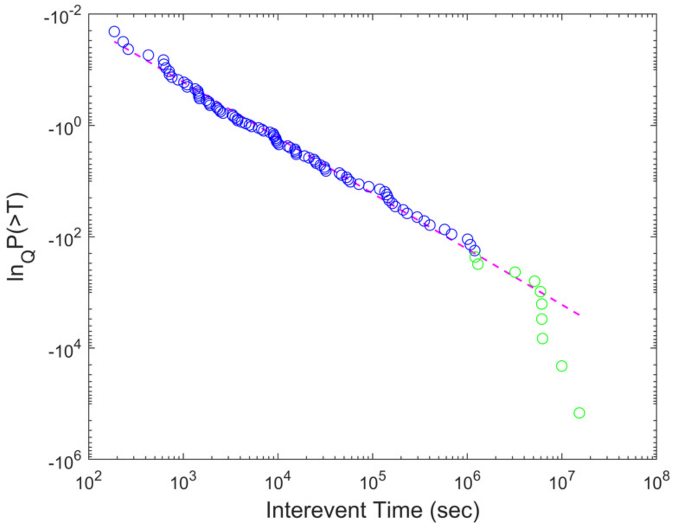

(**b**)

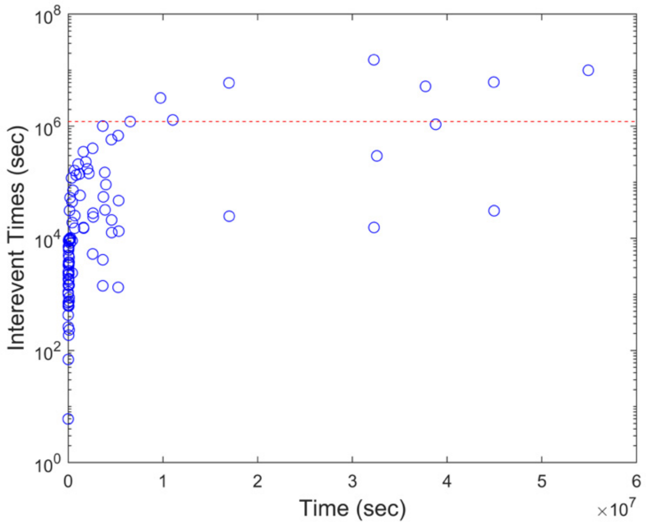

(**c**)

Figure S3. (**a**) The cumulative distribution function of the interevent times of the 1986 M_w 7.7 Kermadec Island Earthquake (New Zealand). The magenta line is the Q-exponential function fitting with $q = 1.74$. (**b**) The Q-logarithmic function of $P(>T)$ as a function of the interevent times, where the dashed line is the fitting with $q = 1.74$. The deviation from linearity suggests T_c values close to 1x10^6 s. The correlation coefficient for the Q-logarithmic function up to T_c is $R^2 = 0.9916$. (**c**) The evolution of the interevent time (T) as a function of the time (t) since the main event. The T value is indicated by the red dashed line.

4. The 1994 M_w 8.3 Shikotan Earthquake (Russia)

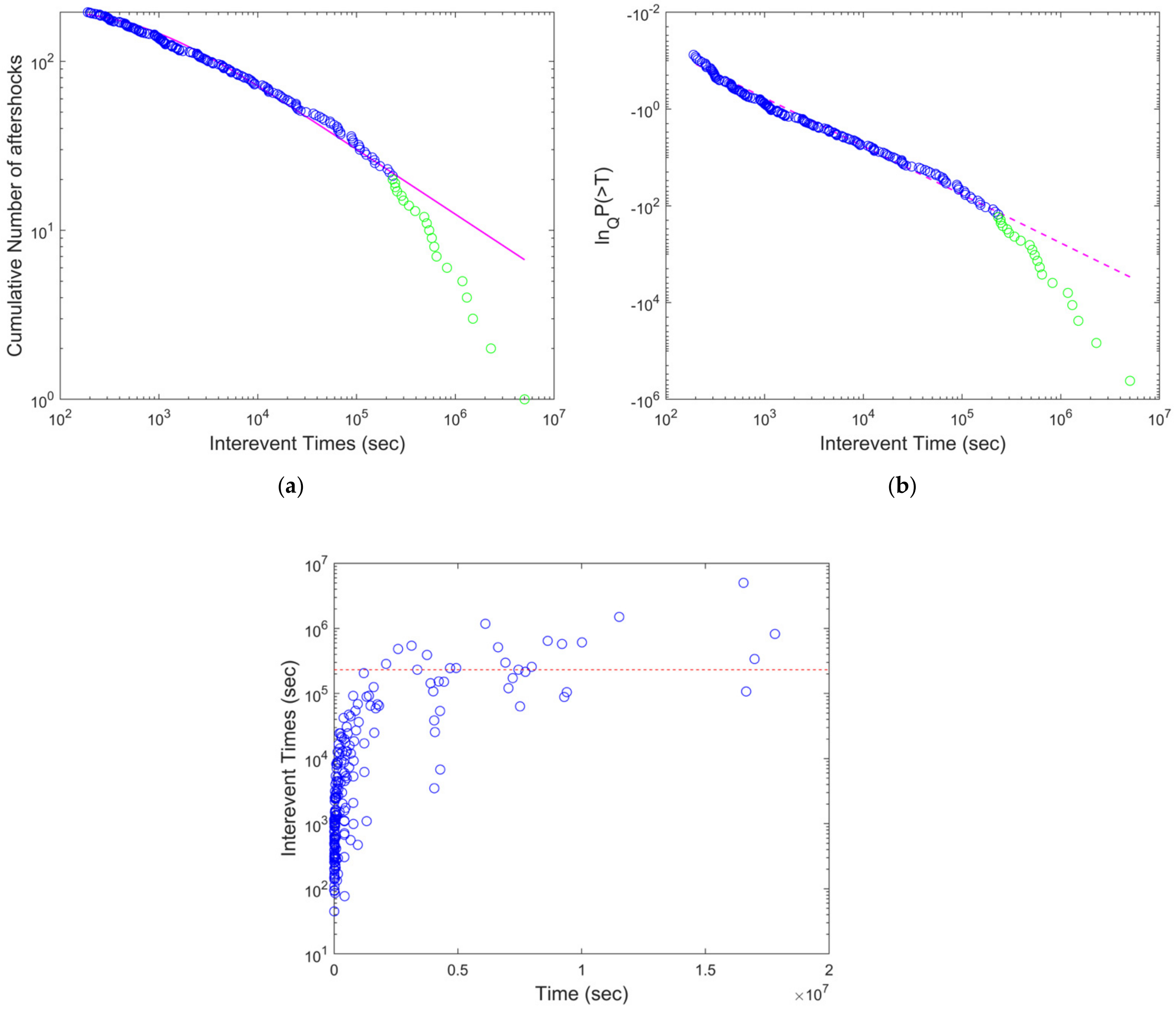

(**a**) (**b**)

(**c**)

Figure S4. (**a**) The cumulative distribution function of the interevent times of the 1994 M_w 8.3 Shikotan Earthquake (Russia). The magenta line is the Q-exponential function fitting with $q = 1.72$. (**b**) The Q-logarithmic function of $P(>T)$ as a function of the interevent times, where the dashed line is the fitting with $q = 1.72$. The deviation from linearity suggests T_c values close to $2\mathrm{x}10^5$ s. The correlation coefficient for the Q-logarithmic function up to T_c is $R^2 = 0.9821$. (**c**) The evolution of the interevent time (T) as a function of the time (t) since the main event. The T value is indicated by the red dashed line.

5. The 1995 M_w 7.7 Panguna Earthquake (Papua New Guinea)

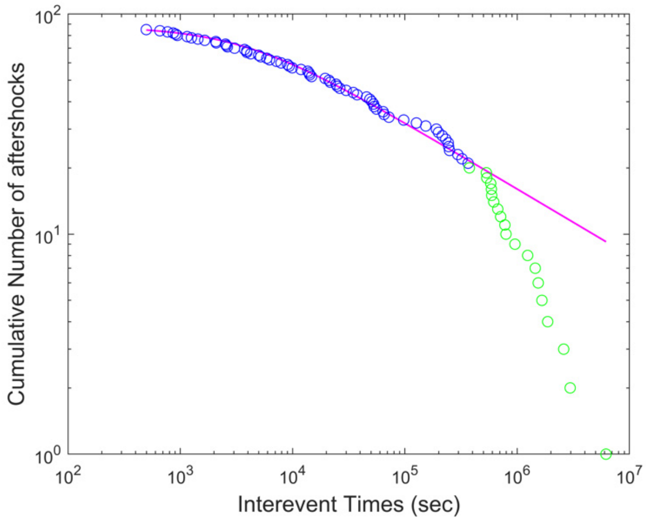

(**a**)

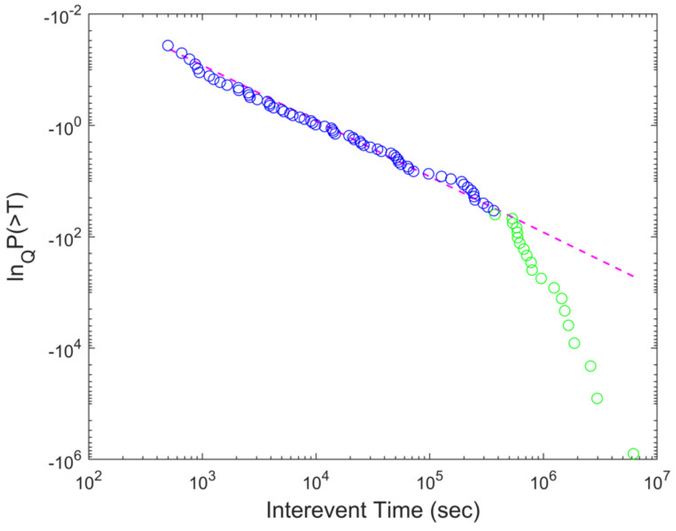

(**b**)

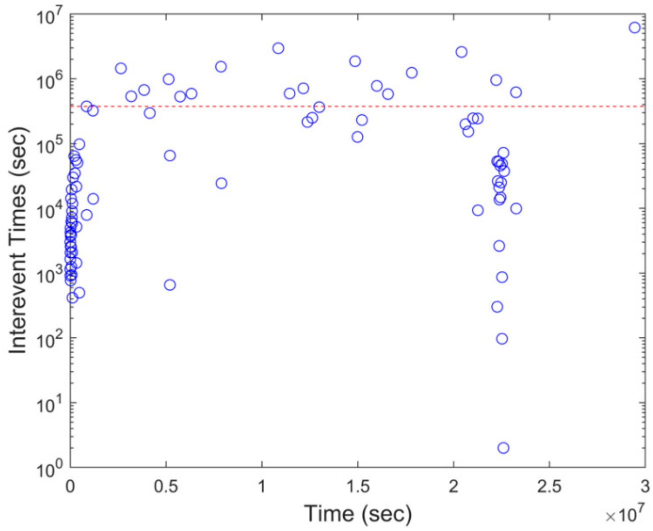

(**c**)

Figure S5. (**a**) The cumulative distribution function of the interevent times of the 1995 M_w 7.7 Panguna Earthquake (Papua New Guinea). The magenta line is the Q-exponential function fitting with q = 1.77. (**b**) The Q-logarithmic function of *P(>T)* as a function of the interevent times, where the dashed line is the fitting with q = 1.77. The deviation from linearity suggests T_c values close to 4x10^5 s. The correlation coefficient for the Q-logarithmic function up to T_c is R^2 = 0.9609. (**c**) The evolution of the interevent time (*T*) as a function of the time (t) since the main event. The *T* value is indicated by the red dashed line.

6. The 1995 M_w 7.9 Kuril Islands Earthquake (Russia)

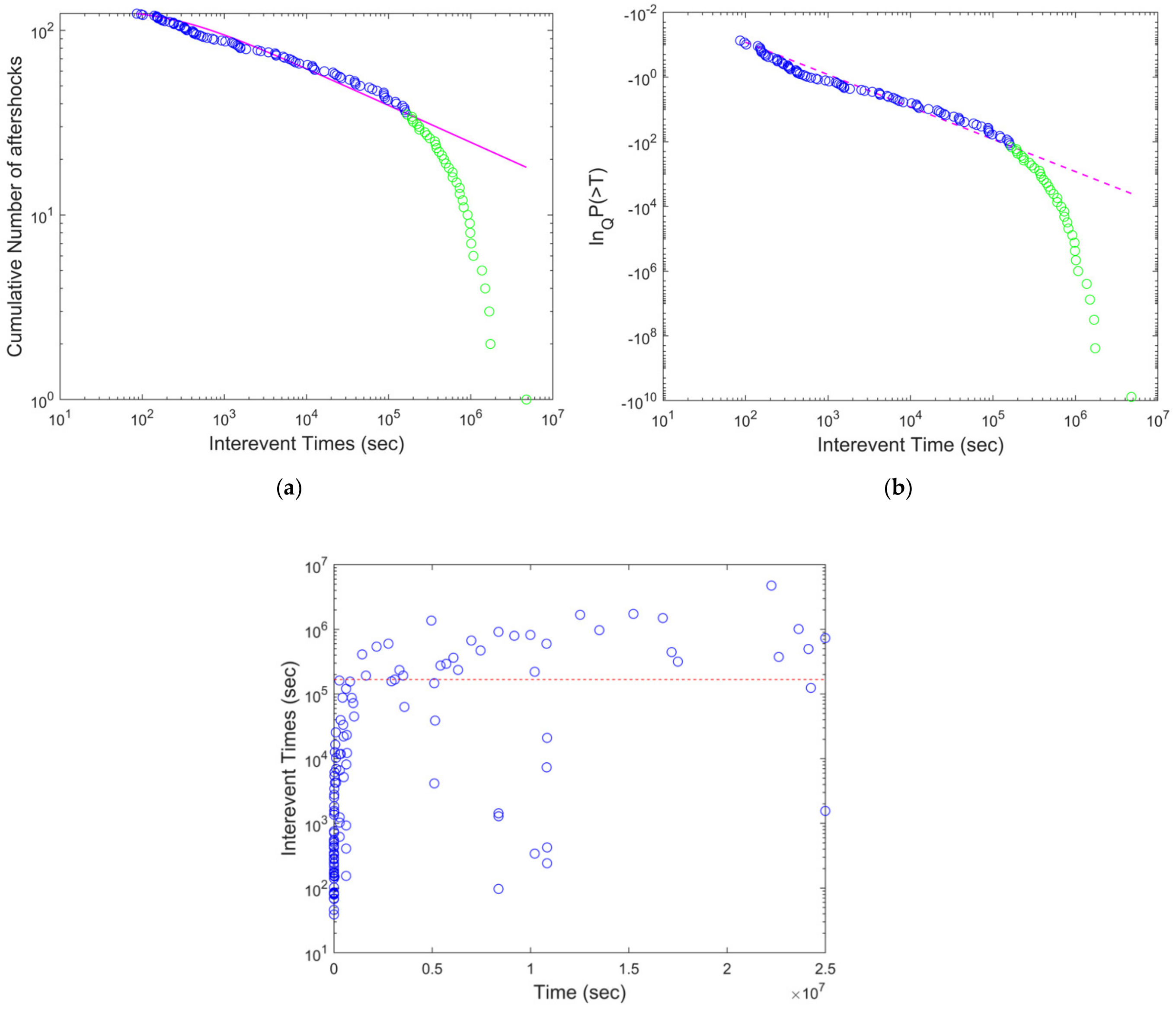

(**a**) (**b**)

(**c**)

Figure S6. (**a**) The cumulative distribution function of the interevent times of the 1995 M_w 7.9 Kuril Islands Earthquake (Russia). The magenta line is the Q-exponential function fitting with $q = 1.83$. (**b**) The Q-logarithmic function of *P(>T)* as a function of the interevent times, where the dashed line is the fitting with $q = 1.83$. The deviation from linearity suggests T_c values close to $2x10^5$ s. The correlation coefficient for the Q-logarithmic function up to T_c is $R^2 = 0.9666$. (**c**) The evolution of the interevent time (*T*) as a function of the time (t) since the main event. The *T* value is indicated by the red dashed line.

7. The 2000 M_w 7.9 Bengkulu Earthquake (Indonesia)

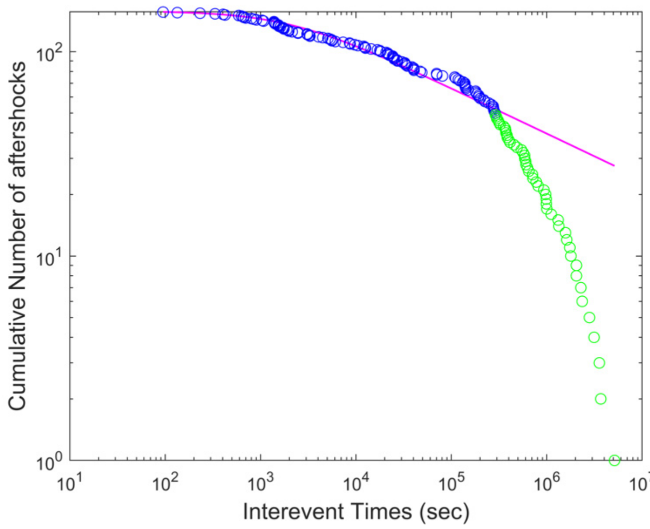

(**a**)

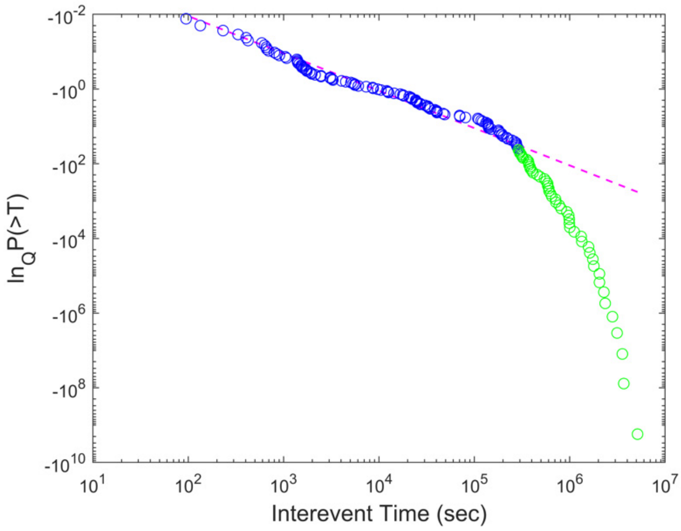

(**b**)

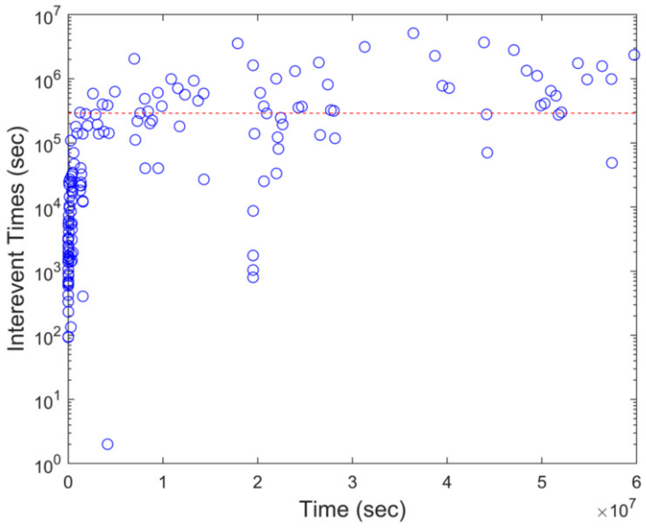

(**c**)

Figure S7. (**a**) The cumulative distribution function of the interevent times for the 2000 M_w 7.9 Bengkulu Earthquake (Indonesia). The magenta line is the Q-exponential function fitting with q = 1.82. (**b**) The Q-logarithmic function of $P(>T)$ as a function of the interevent times, where the dashed line is the fitting with q = 1.82. The deviation from linearity suggests T_c values close to 3x10^5 s. The correlation coefficient for the Q-logarithmic function up to T_c is R^2 = 0.9346. (**c**) The evolution of the interevent time (T) as a function of the time (t) since the main event. The T value is indicated by the red dashed line.

8. The 2000 M_w 8.0 Rabaul Earthquake (Papa New Guinea)

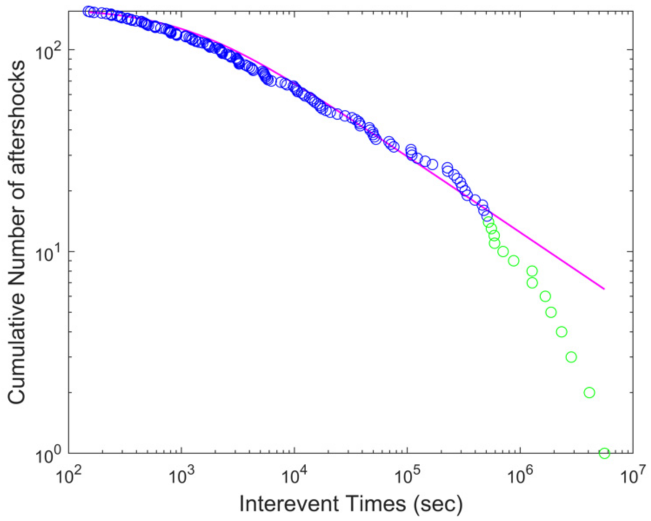

(**a**)

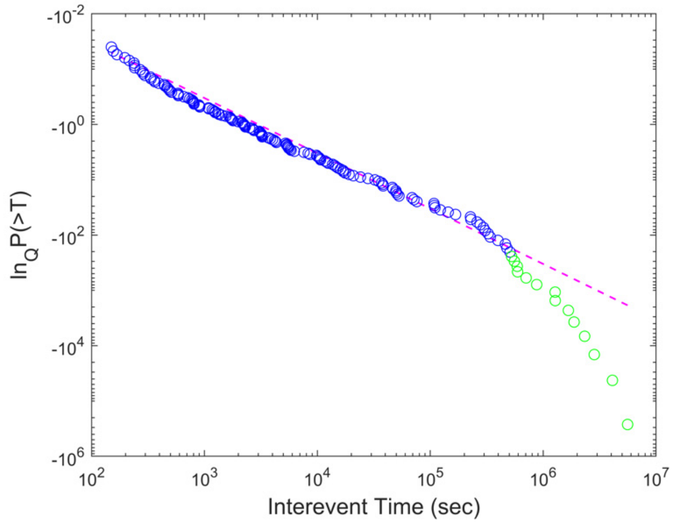

(**b**)

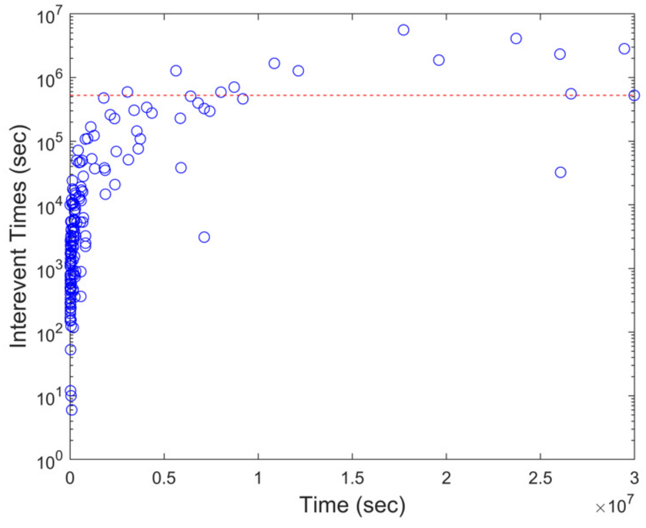

(**c**)

Figure S8. (**a**) The cumulative distribution function of the interevent times for the 2000 M_w 8.0 Rabaul Earthquake (Papa New Guinea). The magenta line is the Q-exponential function fitting with q = 1.73. (**b**) The Q-logarithmic function of $P(>T)$ as a function of the interevent times, where the dashed line is the fitting with q = 1.73. The deviation from linearity suggests T_c values close to 5×10^5 s. The correlation coefficient for the Q-logarithmic function up to T_c is $R^2 = 0.9630$. (**c**) The evolution of the interevent time (T) as a function of the time (t) since the main event. The T value is indicated by the red dashed line.

9. The 2001 M_w 8.4 Atico Earthquake (Peru)

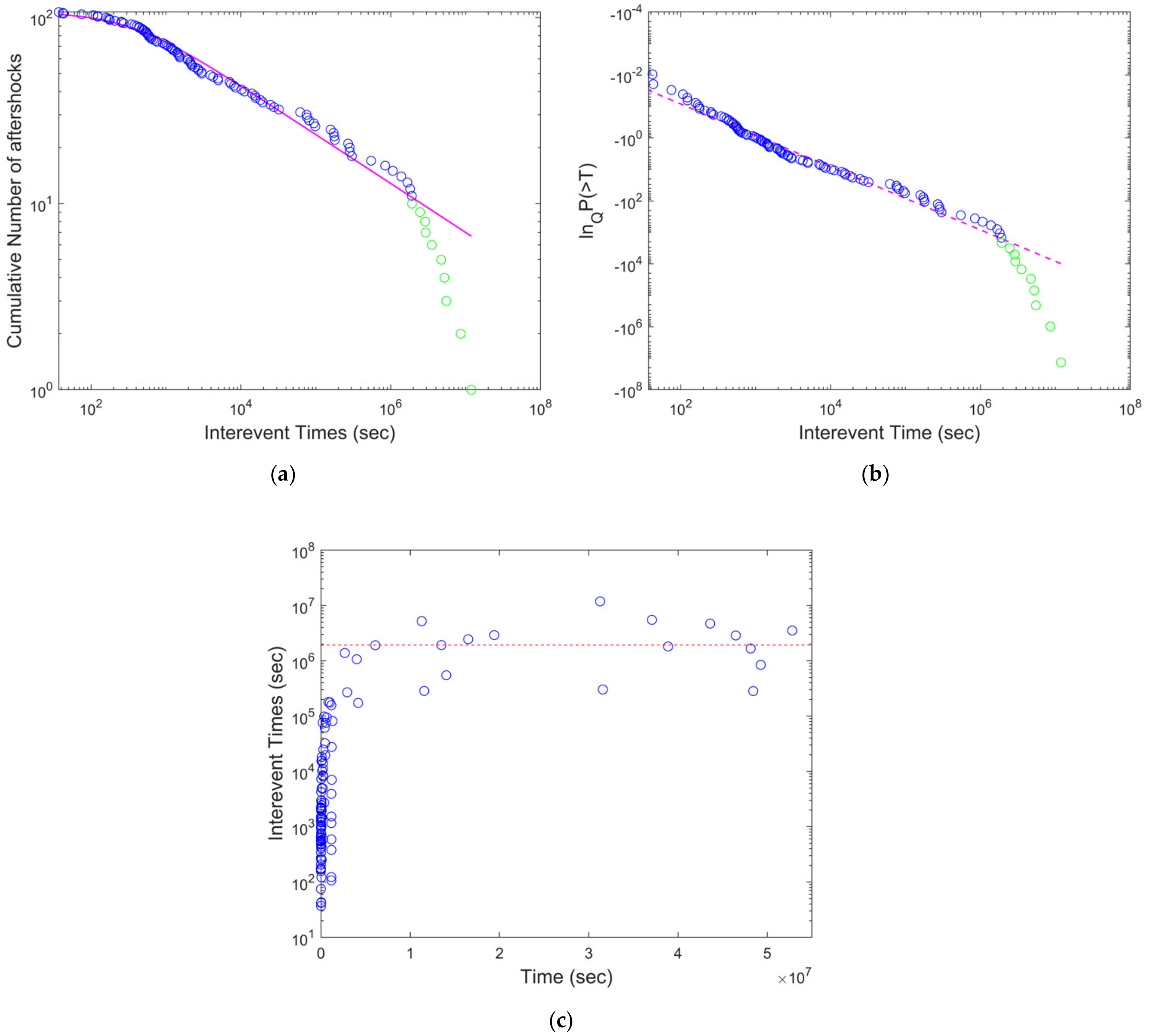

Figure S9. (**a**) The cumulative distribution function of the interevent times for the 2001 M_w 8.4 Atico Earthquake (Peru). The magenta line is the Q-exponential function fitting with q = 1.79. (**b**) The Q-logarithmic function of $P(>T)$ as a function of the interevent times, where the dashed line is the fitting with q = 1.79. The deviation from linearity suggests T_c values close to 2x10^6 s. The correlation coefficient for the Q-logarithmic function up to T_c is R^2 = 0.9400. (**c**) The evolution of the interevent time (T) as a function of the time (t) since the main event. The T value is indicated by the red dashed line.

10. The 2003 M_w 8.3 Kashiro Earthquake (Japan)

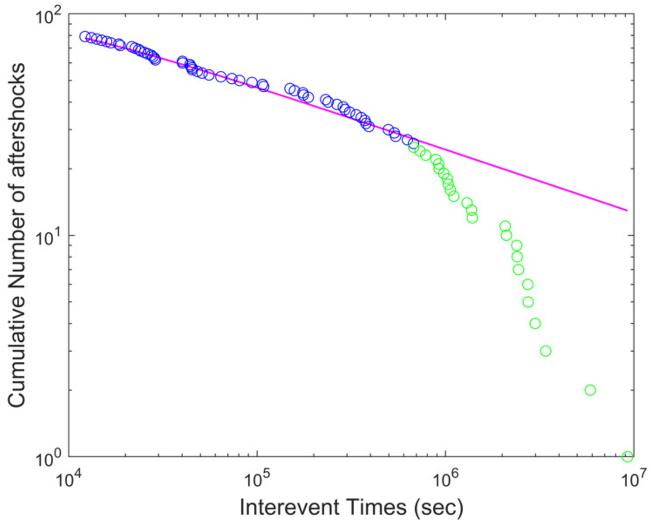

(**a**)

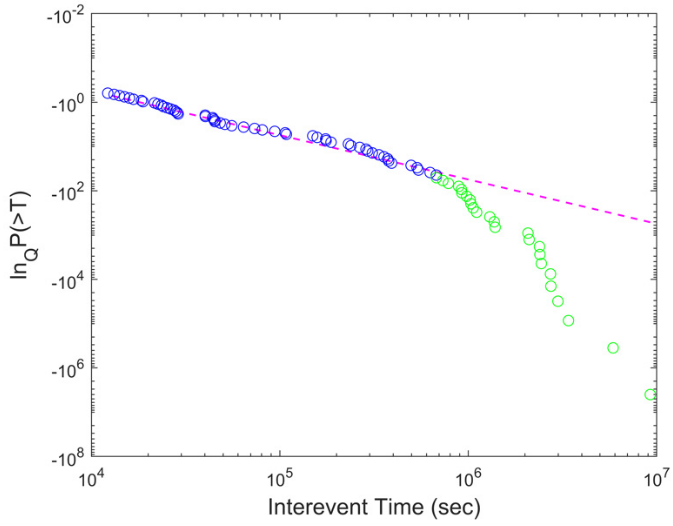

(**b**)

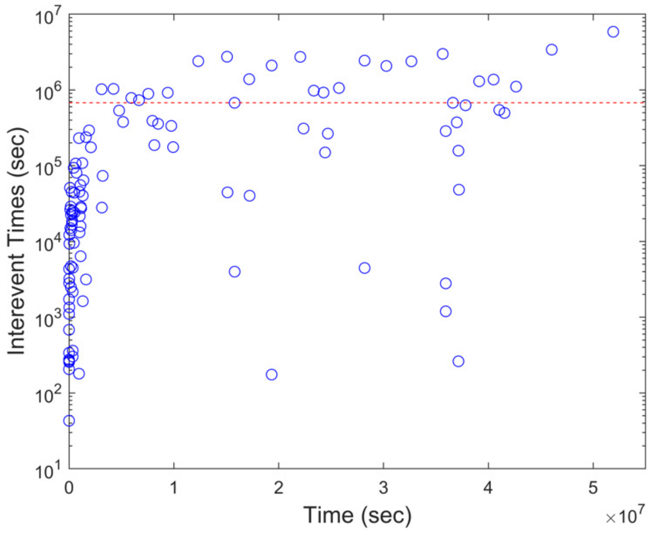

(**c**)

Figure S10. (**a**) The cumulative distribution function of the interevent times for the 2003 M_w 8.3 Kashiro Earthquake (Japan). The magenta line is the Q-exponential function fitting with $q = 1.78$. (**b**) The Q-logarithmic function of $P(>T)$ as a function of the interevent times, where the dashed line is the fitting with q = 1.78. The deviation from linearity suggests T_c values close to 6x10^5 s. The correlation coefficient for the Q-logarithmic function up to T_c is $R^2 = 0.9689$. (**c**) The evolution of the interevent time (T) as a function of the time (t) since the main event. The T value is indicated by the red dashed line.

11. The 2004 M_w 9.1 Sumatra–Andaman Islands Earthquake

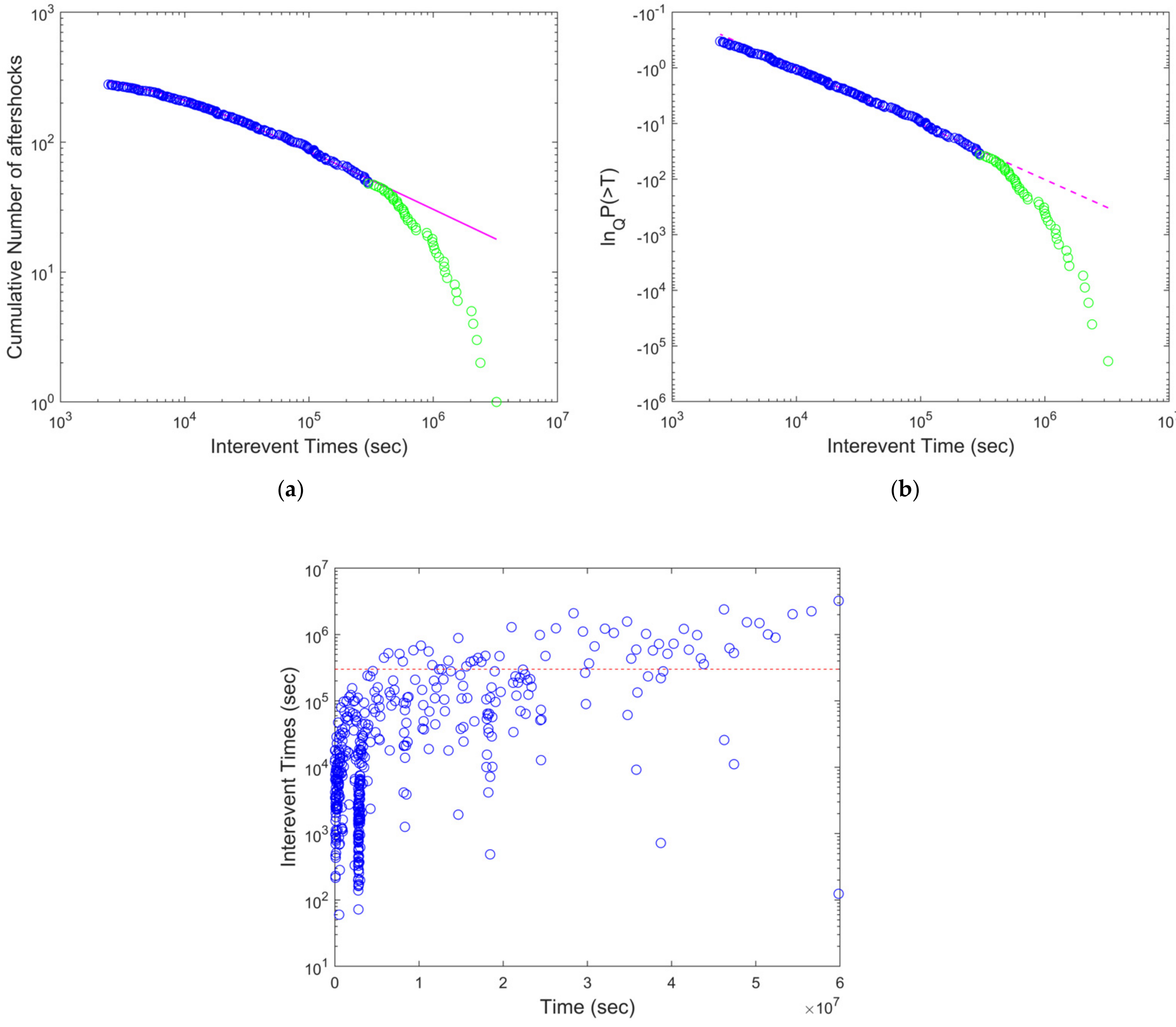

(**a**) (**b**)

(**c**)

Figure S11. (**a**) The cumulative distribution function of the interevent times for the 2004 M_w 9.1 Sumatra–Andaman Islands Earthquake. The magenta line is the Q-exponential function fitting with q = 1.69. (**b**) The Q-logarithmic function of $P(>T)$ as a function of the interevent times, where the dashed line is the fitting with q = 1.69. The deviation from linearity suggests T_c values close to 3x10^5 s. The correlation coefficient for the Q-logarithmic function up to T_c is R^2 = 0.9923. (**c**) The evolution of the interevent time (T) as a function of the time (t) since the main event. The T value is indicated by the red dashed line.

12. The 2005 M_w 8.6 Singkil Earthquake (Indonesia)

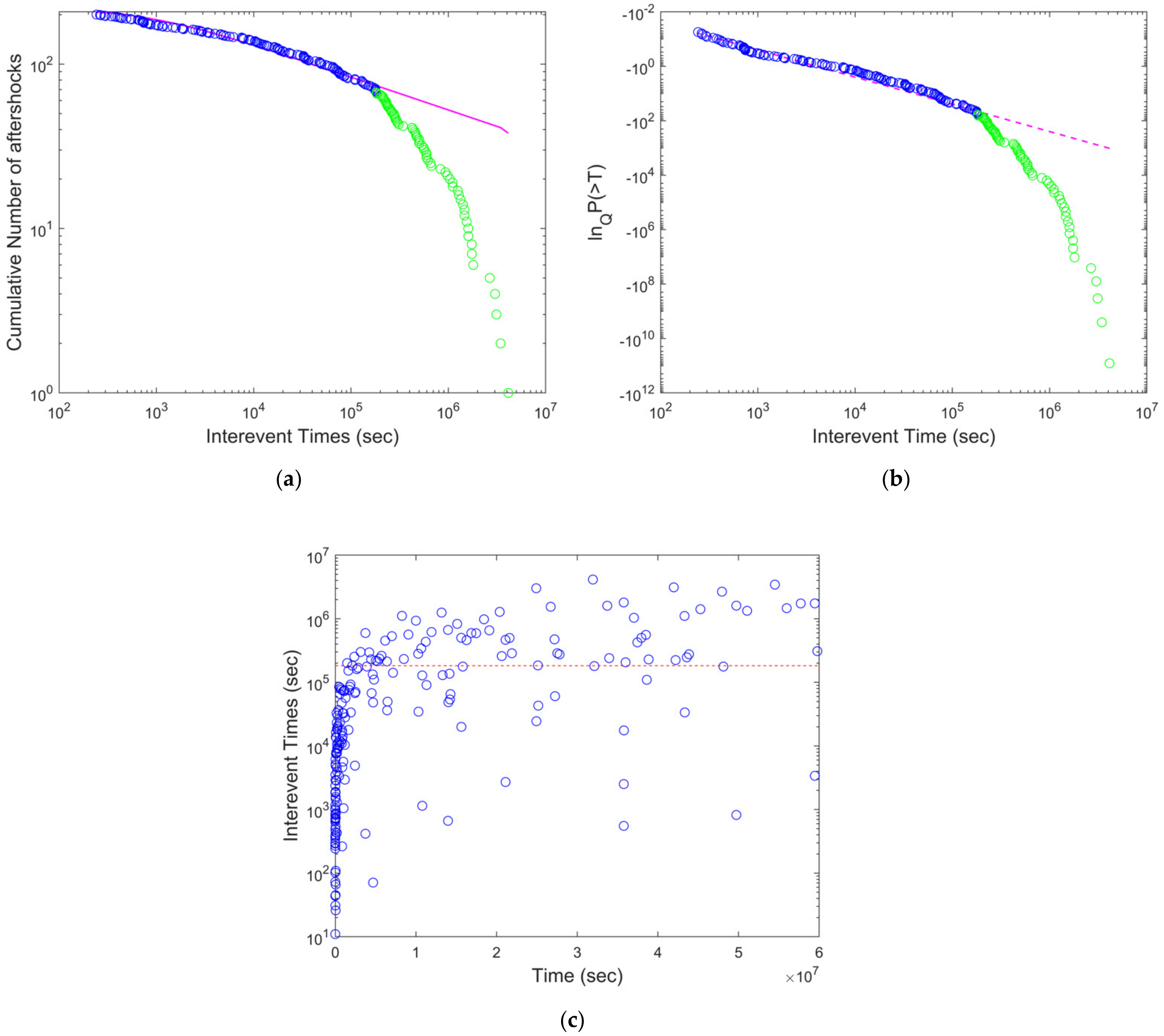

Figure S12. (**a**) The cumulative distribution function of the interevent times for the 2005 M_w 8.6 Singkil Earthquake (Indonesia). The magenta line is the Q-exponential function fitting with $q = 1.83$. (**b**) The Q-logarithmic function of $P(>T)$ as a function of the interevent times, where the dashed line is the fitting with $q = 1.83$. The deviation from linearity suggests T_c values close to $2\text{x}10^5$ s. The correlation coefficient for the Q-logarithmic function up to T_c is $R^2 = 0.9622$. (**c**) The evolution of the interevent time (T) as a function of the time (t) since the main event. The T value is indicated by the red dashed line.

13. The 2006 M_w 8.0 Pangai Earthquake (Tonga)

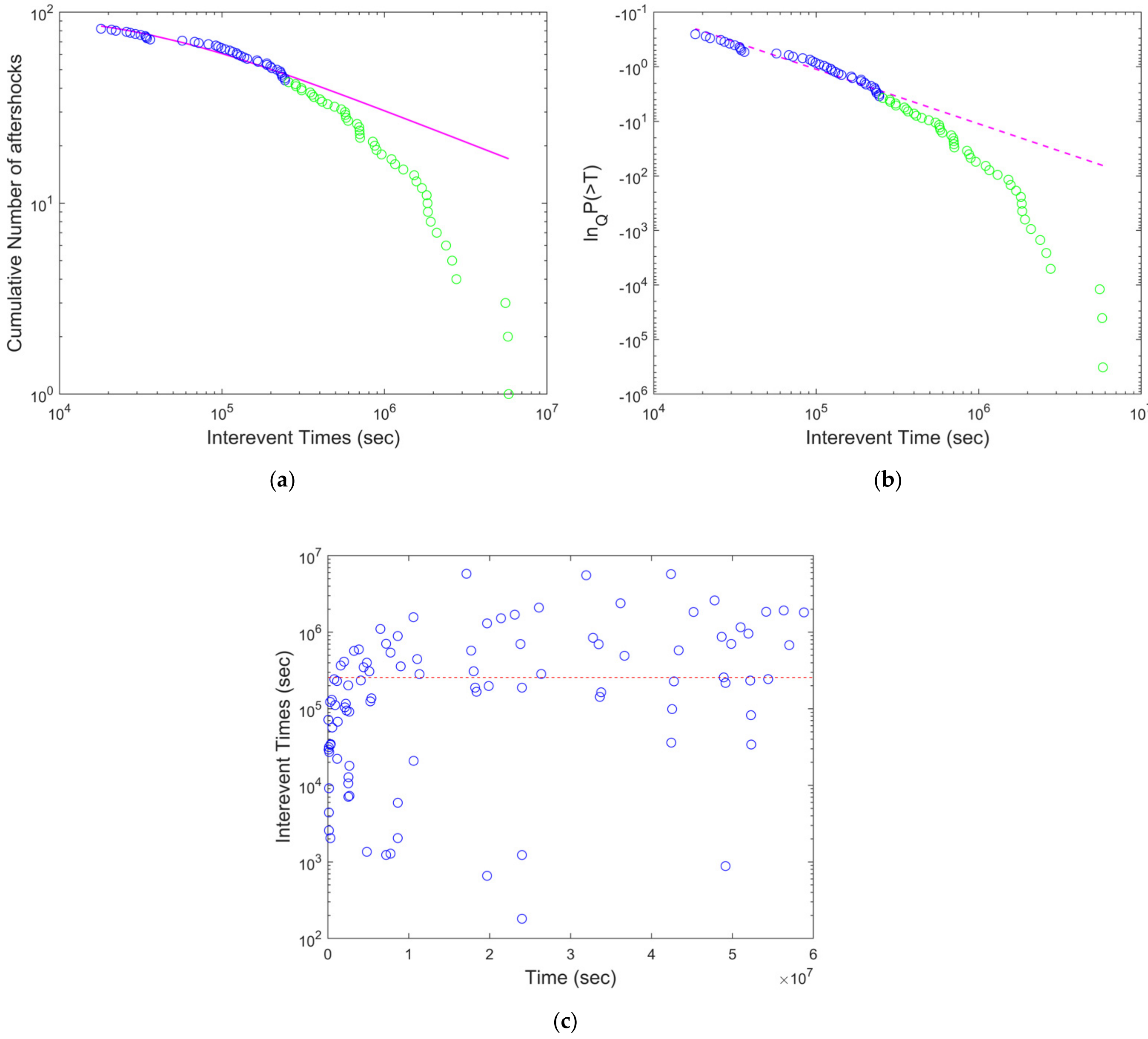

Figure S13. (**a**) The cumulative distribution function of the interevent times for the 2006 M_w 8.0 Pangai Earthquake (Tonga). The magenta line is the Q-exponential function fitting with $q = 1.75$. (**b**) The Q-logarithmic function of *P(>T)* as a function of the interevent times, where the dashed line is the fitting with q = 1.75. The deviation from linearity suggests T_c values close to 3x10^5 s. The correlation coefficient for the Q-logarithmic function up to T_c is $R^2 = 0.9494$. (**c**) The evolution of the interevent time (*T)* as a function of the time (t) since the main event. The *T* value is indicated by the red dashed line.

14. The 2006 M_w 7.7 Singaparna Earthquake (Indonesia)

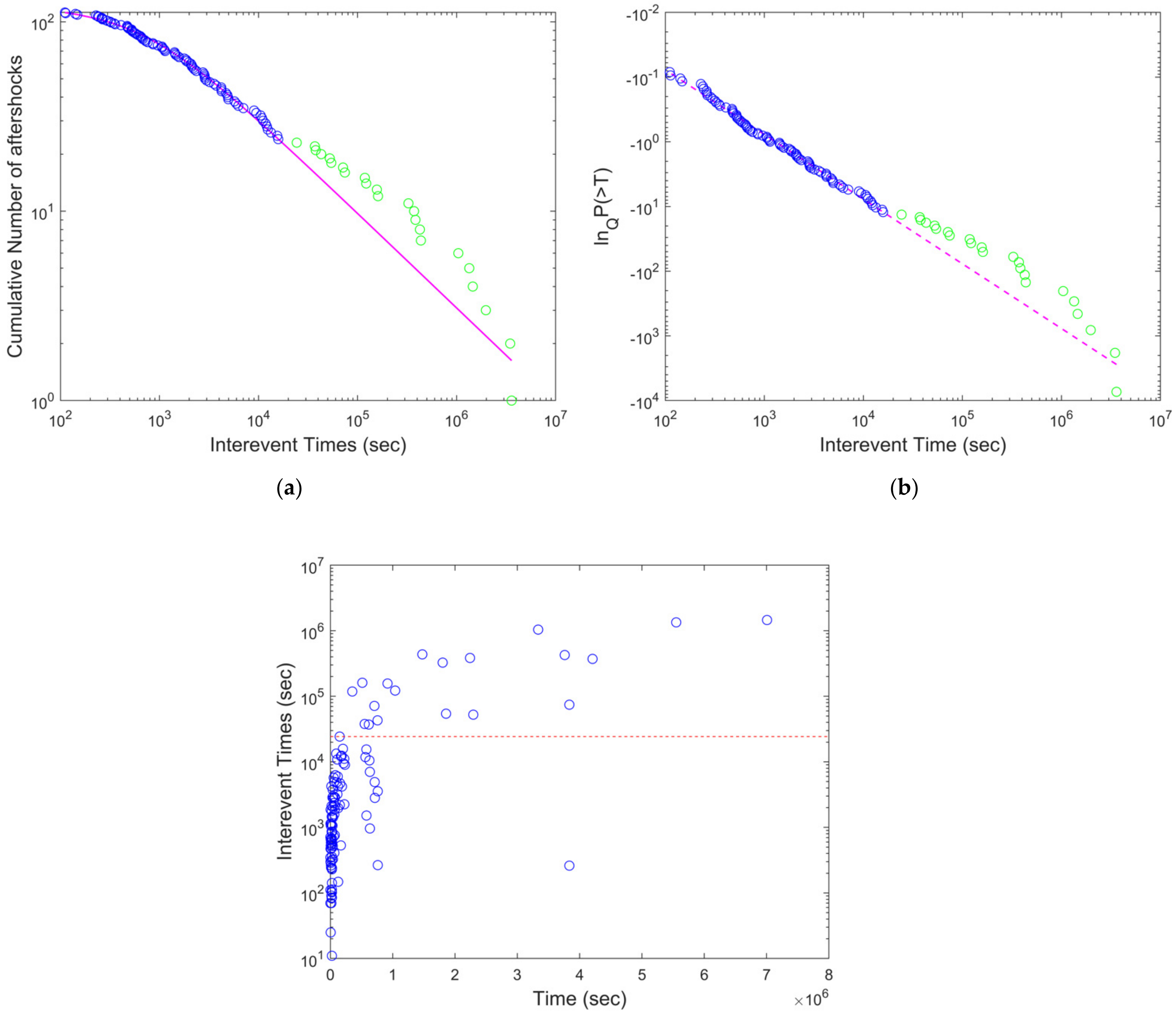

(**a**) (**b**) (**c**)

Figure S14. (**a**) The cumulative distribution function of the interevent times for the 2006 M_w 7.7 Singaparna Earthquake (Indonesia). The magenta line is the Q-exponential function fitting with q = 1.67. (**b**) The Q-logarithmic function of *P(>T)* as a function of the interevent times, where the dashed line is the fitting with q = 1.67. The deviation from linearity suggests T_c values close to 2x10^4 s. The correlation coefficient for the Q-logarithmic function up to T_c is R^2 = 0.9909. (**c**) The evolution of the interevent time (*T)* as a function of the time (t) since the main event. The *T* value is indicated by the red dashed line.

15. The 2007 M_w 8.1 Gizo Earthquake (Solomon Islands)

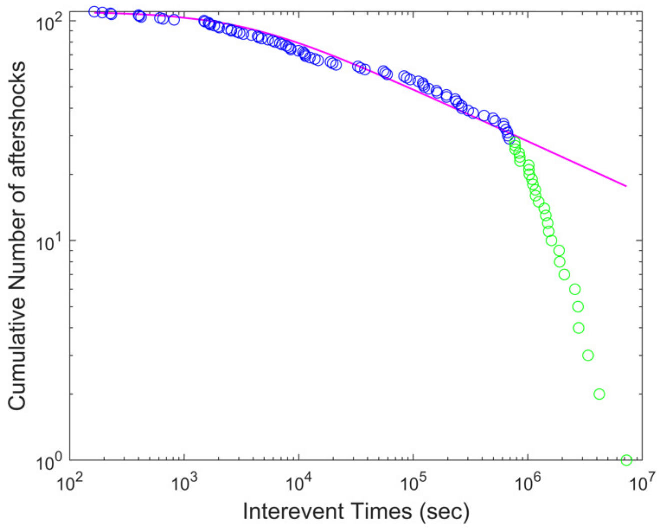

(**a**)

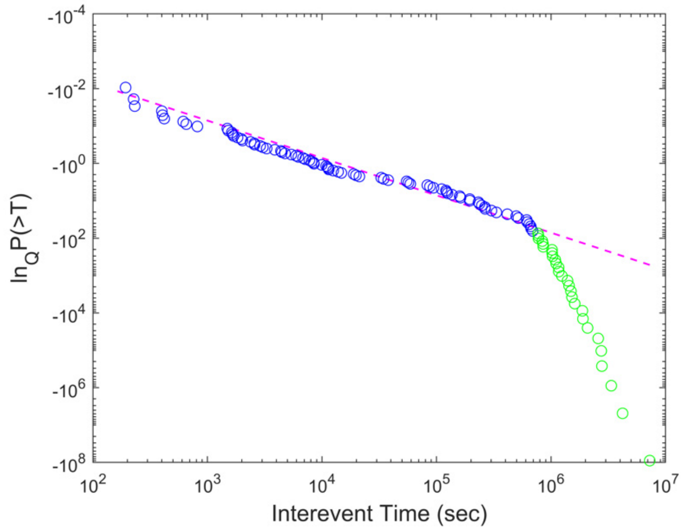

(**b**)

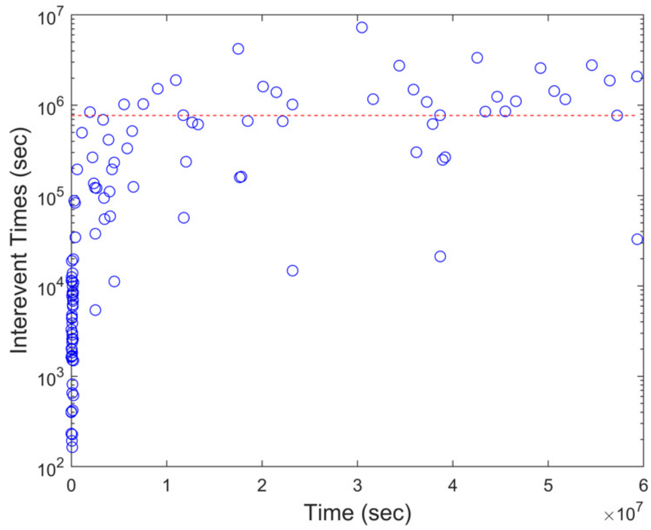

(**c**)

Figure S15. (**a**) The cumulative distribution function of the interevent times for the 2007 M_w 8.1 Gizo Earthquake (Solomon Islands). The magenta line is the Q-exponential function fitting with $q = 1.81$. (**b**) The Q-logarithmic function of $P(>T)$ as a function of the interevent times, where the dashed line is the fitting with $q = 1.81$. The deviation from linearity suggests T_c values close to 8x10^5 s. The correlation coefficient for the Q-logarithmic function up to T_c is $R^2 = 0.9433$. (**c**) The evolution of the interevent time (T) as a function of the time (t) since the main event. The T value is indicated by the red dashed line.

16. The 2007 M_w 8.5 Bengkulu Earthquake (Indonesia)

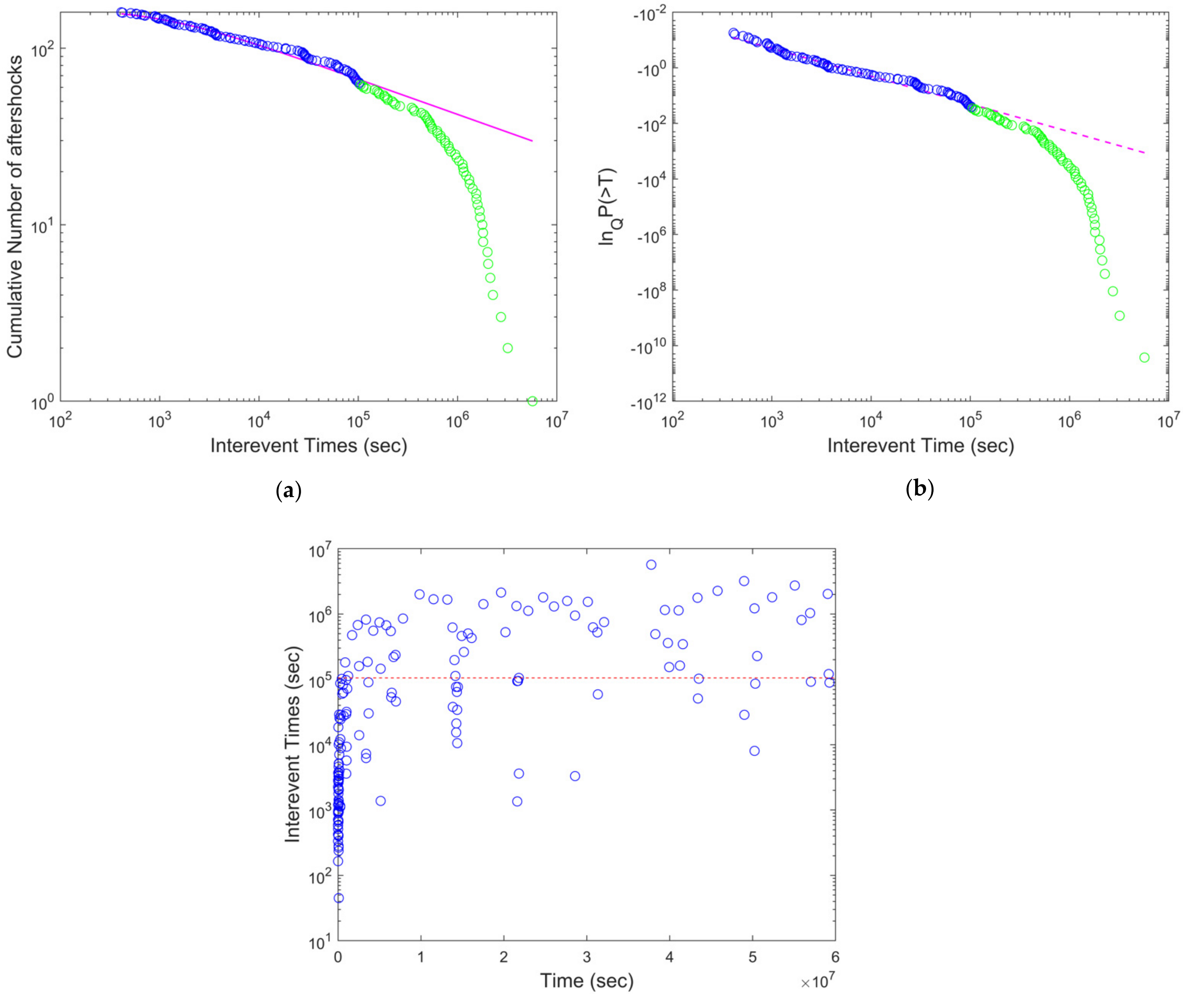

Figure S16. (**a**) The cumulative distribution function of the interevent times for the 2007 M_w 8.5 Bengkulu Earthquake (Indonesia). The magenta line is the Q-exponential function fitting with q = 1.83. (**b**) The Q-logarithmic function of *P(>T)* as a function of the interevent times, where the dashed line is the fitting with q = 1.83. The deviation from linearity suggests T_c values close to 1x10^5 s. The correlation coefficient for the Q-logarithmic function up to T_c is R^2 = 0.9305. (**c**) The evolution of the interevent time (*T*) as a function of the time (t) since the main event. The *T* value is indicated by the red dashed line.

17. The 2009 M_w 7.2 Sarangani Earthquake (Philippines)

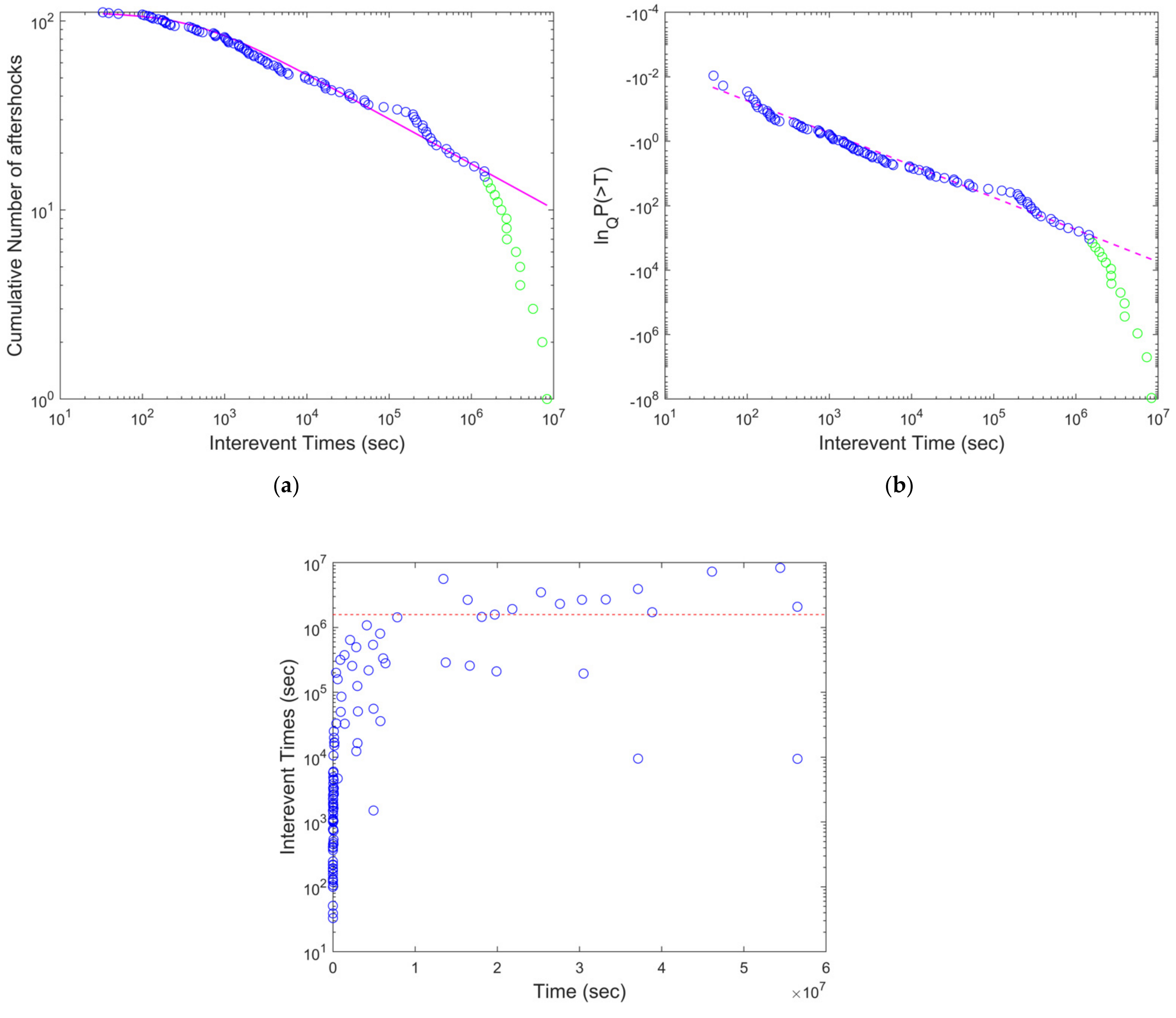

Figure S17. (**a**) The cumulative distribution function of the interevent times for the 2009 M_w 7.2 Sarangani Earthquake (Philippines). The magenta line is the Q-exponential function fitting with q = 1.81. (**b**) The Q-logarithmic function of *P(>T)* as a function of the interevent times, where the dashed line is the fitting with q = 1.81. The deviation from linearity suggests T_c values close to 2x10^6 s. The correlation coefficient for the Q-logarithmic function up to T_c is R^2 = 0.9694. (**c**) The evolution of the interevent time (*T*) as a function of the time (t) since the main event. The *T* value is indicated by the red dashed line.

18. The 2009 M_w 8.1 Matavai Earthquake (Samoa)

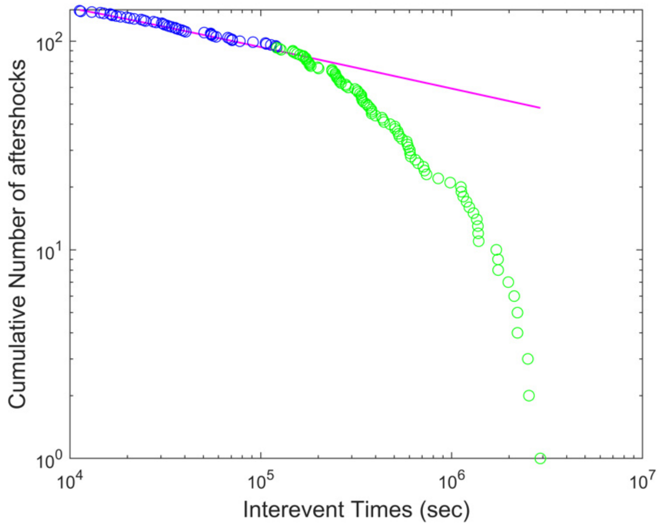

(**a**)

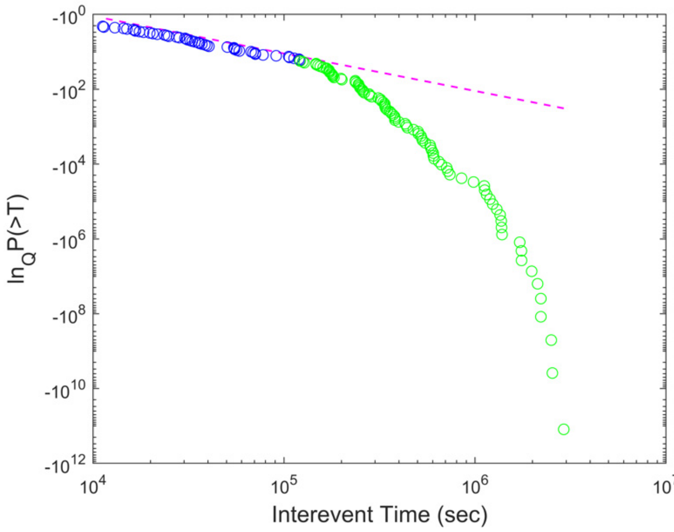

(**b**)

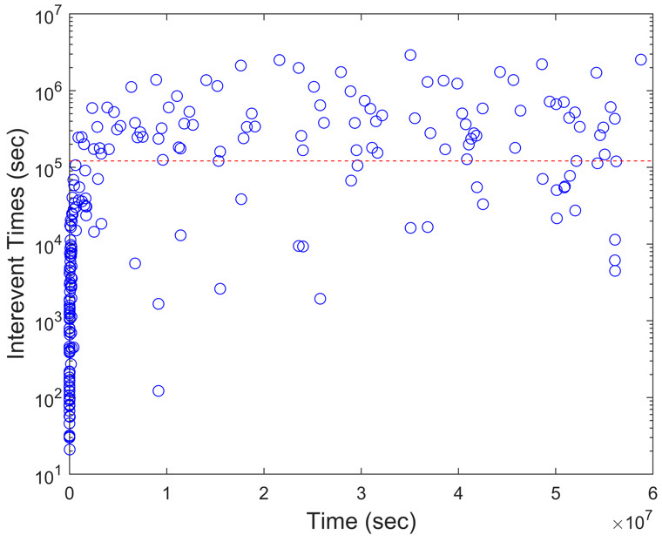

(**c**)

Figure S18. (**a**) The cumulative distribution function of the interevent times for the 2009 M_w 8.1 Matavai Earthquake (Samoa). The magenta line is the Q-exponential function fitting with $q = 1.83$. (**b**) The Q-logarithmic function of $P(>T)$ as a function of the interevent times, where the dashed line is the fitting with $q = 1.83$. The deviation from linearity suggests T_c values close to $1\text{x}10^5$ s. The correlation coefficient for the Q-logarithmic function up to T_c is $R^2 = 0.9897$. (**c**) The evolution of the interevent time (T) as a function of the time (t) since the main event. The T value is indicated by the red dashed line.

19. The 2009 M_w 7.8 Sola Earthquake (Vanuatu)

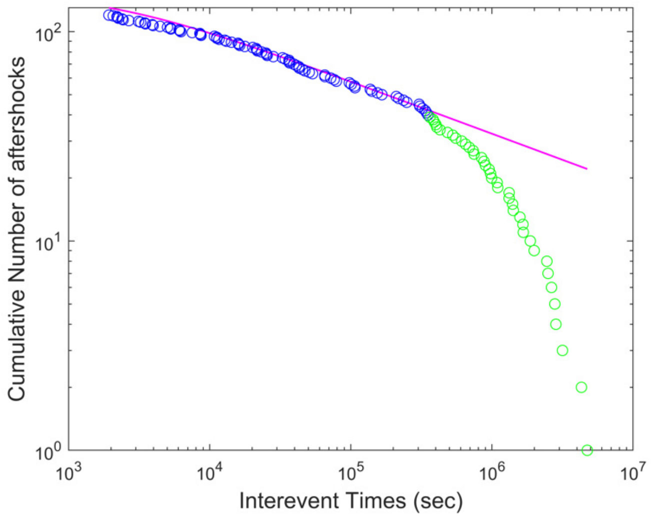

(**a**)

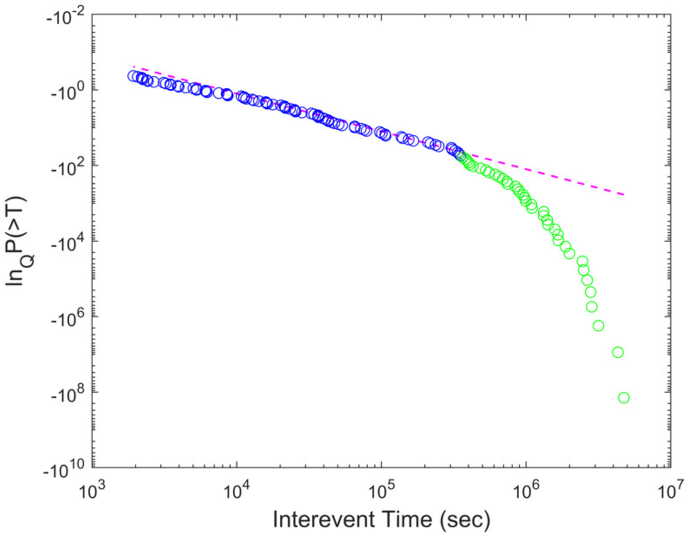

(**b**)

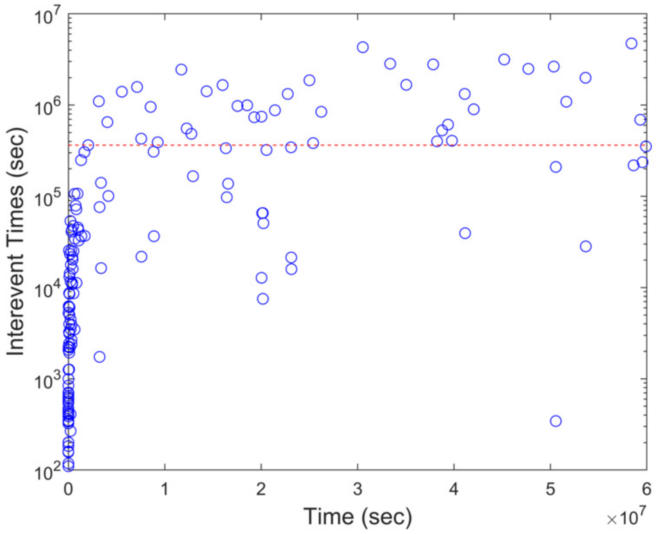

(**c**)

Figure S19. (**a**) The cumulative distribution function of the interevent times for the 2009 M_w 7.8 Sola Earthquake (Vanuatu). The magenta line is the Q-exponential function fitting with $q = 1.80$. (**b**) The Q-logarithmic function of *P(>T)* as a function of the interevent times, where the dashed line is the fitting with q = 1.80. The deviation from linearity suggests T_c values close to 4x10^5 s. The correlation coefficient for the Q-logarithmic function up to T_c is $R^2 = 0.9864$. (**c**) The evolution of the interevent time (*T*) as a function of the time (t) since the main event. The *T* value is indicated by the red dashed line.

20. The 2010 M_w 8.8 Quirihue Earthquake (Chile)

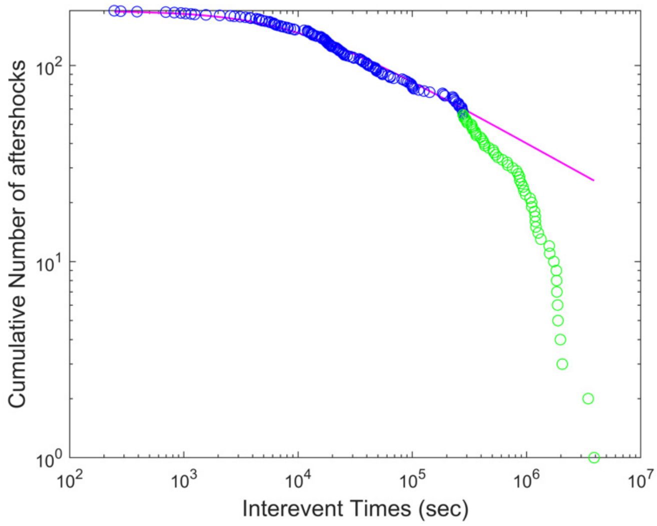

(**a**)

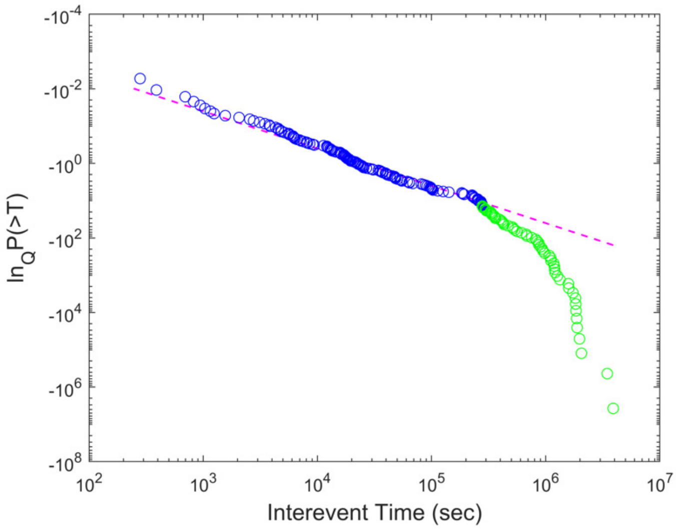

(**b**)

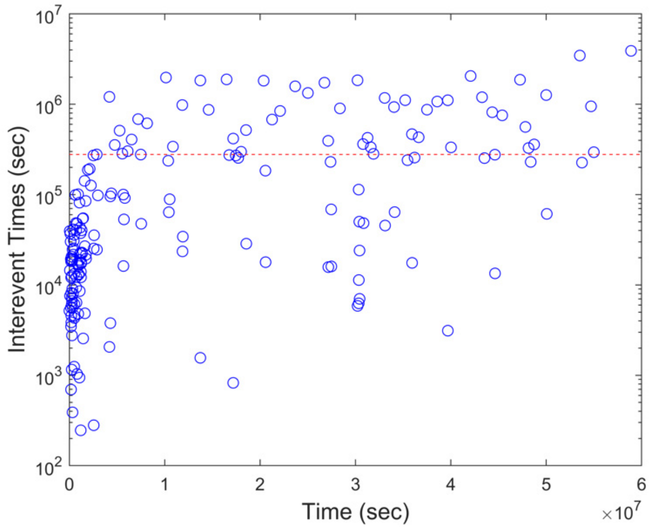

(**c**)

Figure S20. (**a**) The cumulative distribution function of the interevent times for the 2010 M_w 8.8 Quirihue Earthquake (Chile). The magenta line is the Q-exponential function fitting with $q = 1.76$. (**b**) The Q-logarithmic function of *P(>T)* as a function of the interevent times, where the dashed line is the fitting with q = 1.76. The deviation from linearity suggests T_c values close to 3x10^5 s. The correlation coefficient for the Q-logarithmic function up to T_c is $R^2 = 0.9726$. (**c**) The evolution of the interevent time (*T)* as a function of the time (t) since the main event. The *T* value is indicated by the red dashed line.

21. The 2011 M_w 9.1 Great Tohoku (Japan) Earthquake

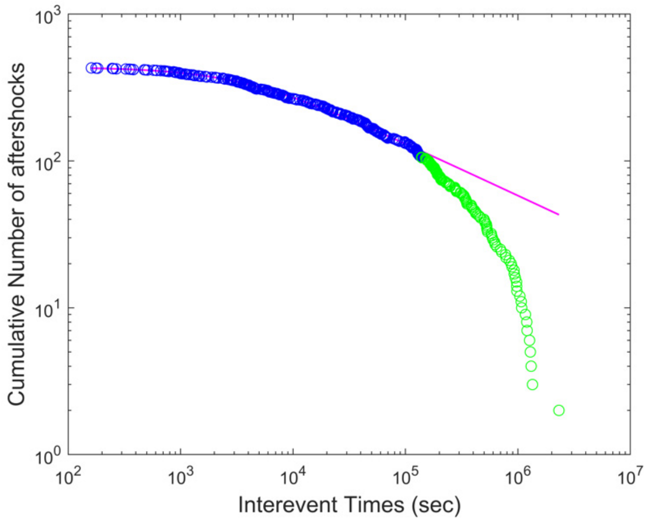

(**a**)

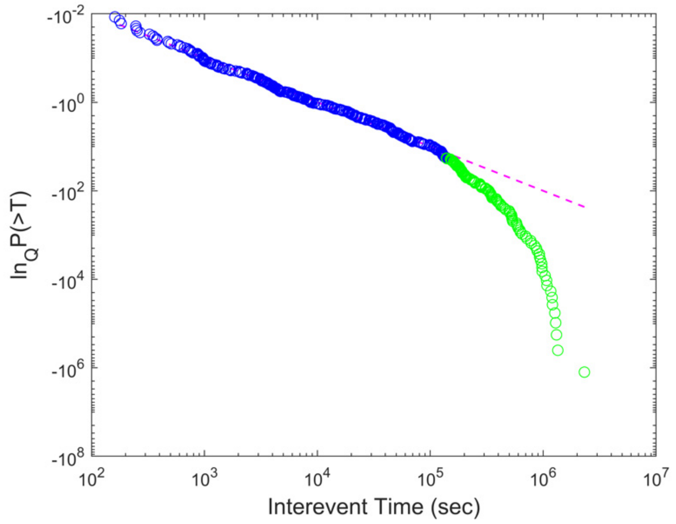

(**b**)

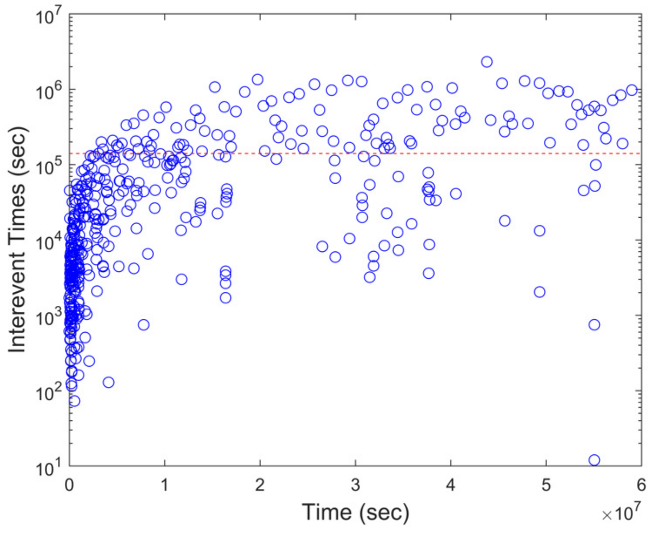

(**c**)

Figure S21. (**a**) The cumulative distribution function of the interevent times for the 2011 M_w 9.1 Great Tohoku (Japan) Earthquake. The magenta line is the Q-exponential function fitting with q = 1.74. (**b**) The Q-logarithmic function of $P(>T)$ as a function of the interevent times, where the dashed line is the fitting with q = 1.74. The deviation from linearity suggests T_c values close to 1x10^5 s. The correlation coefficient for the Q-logarithmic function up to T_c is R^2 = 0.9828. (**c**) The evolution of the interevent time (T) as a function of the time (t) since the main event. The T value is indicated by the red dashed line.

22. The 2011 M_w 7.6 Kermadec Earthquake (New Zealand)

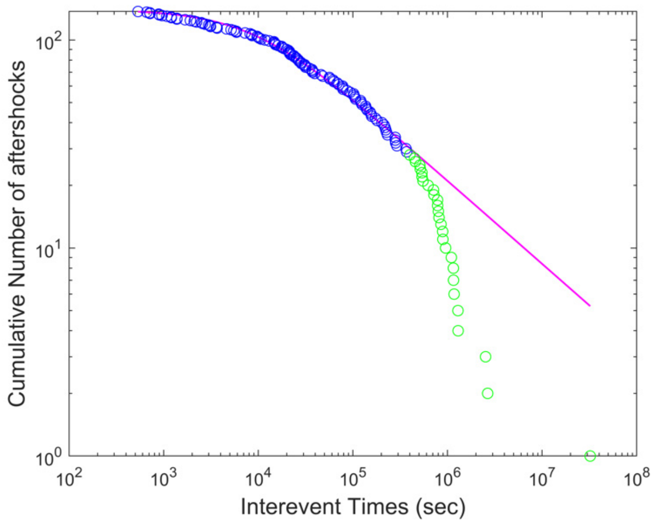

(**a**)

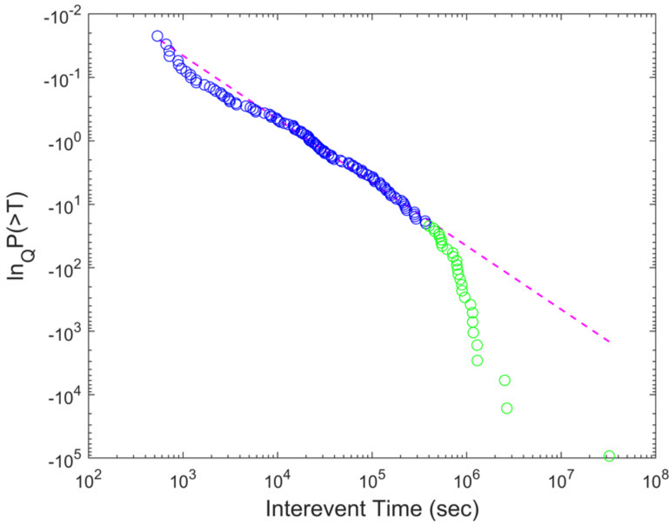

(**b**)

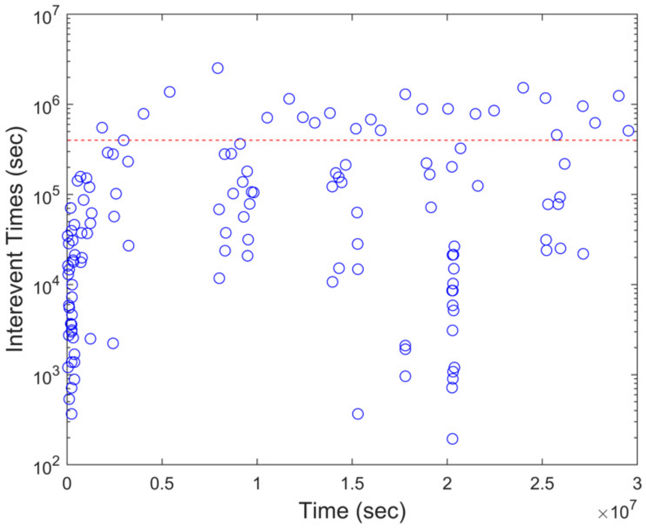

(**c**)

Figure S22. (**a**) The cumulative distribution function of the interevent times for the 2011 M_w 7.6 Kermadec Earthquake (New Zealand). The magenta line is the Q-exponential function fitting with $q = 1.71$. (**b**) The Q-logarithmic function of $P(>T)$ as a function of the interevent times, where the dashed line is the fitting with $q = 1.71$. The deviation from linearity suggests T_c values close to 4×10^5 s. The correlation coefficient for the Q-logarithmic function up to T_c is $R^2 = 0.9821$. (**c**) The evolution of the interevent time (T) as a function of the time (t) since the main event. The T value is indicated by the red dashed line.

23. The 2012 M_w 8.6 West Coast of Northern Sumatra Earthquake

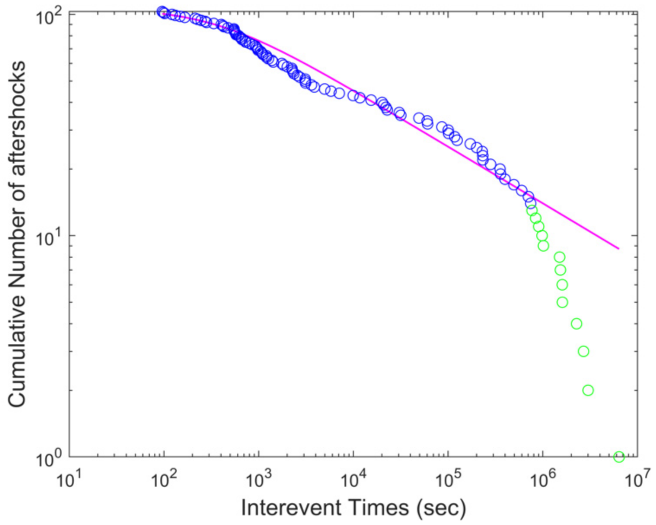

(**a**)

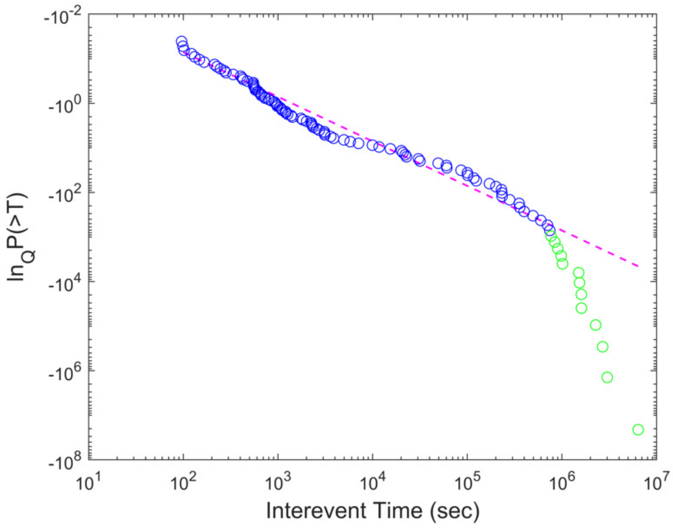

(**b**)

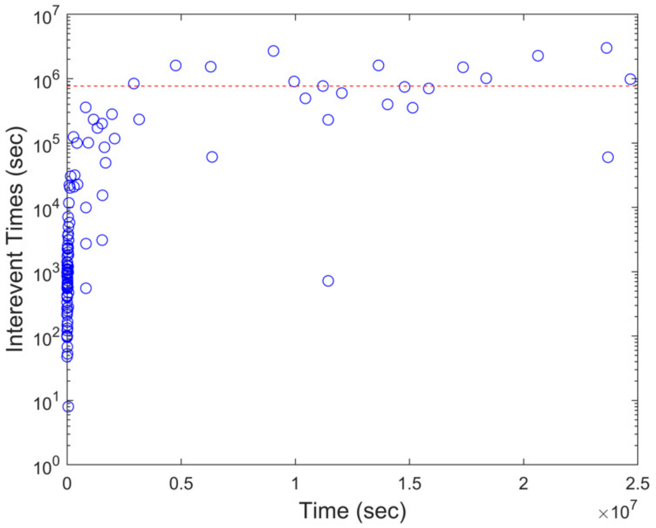

(**c**)

Figure S23. (**a**) The cumulative distribution function of the interevent times for the 2012 M_w 8.6 West Coast of Northern Sumatra Earthquake. The magenta line is the Q-exponential function fitting with q = 1.80. (**b**) The Q-logarithmic function of $P(>T)$ as a function of the interevent times, where the dashed line is the fitting with q = 1.80. The deviation from linearity suggests T_c values close to 8x10^5 s. The correlation coefficient for the Q-logarithmic function up to T_c is R^2 = 0.9411. (**c**) The evolution of the interevent time (T) as a function of the time (t) since the main event. The T value is indicated by the red dashed line.

24. The 2013 M_w 7.0 Atka Earthquake (Alaska)

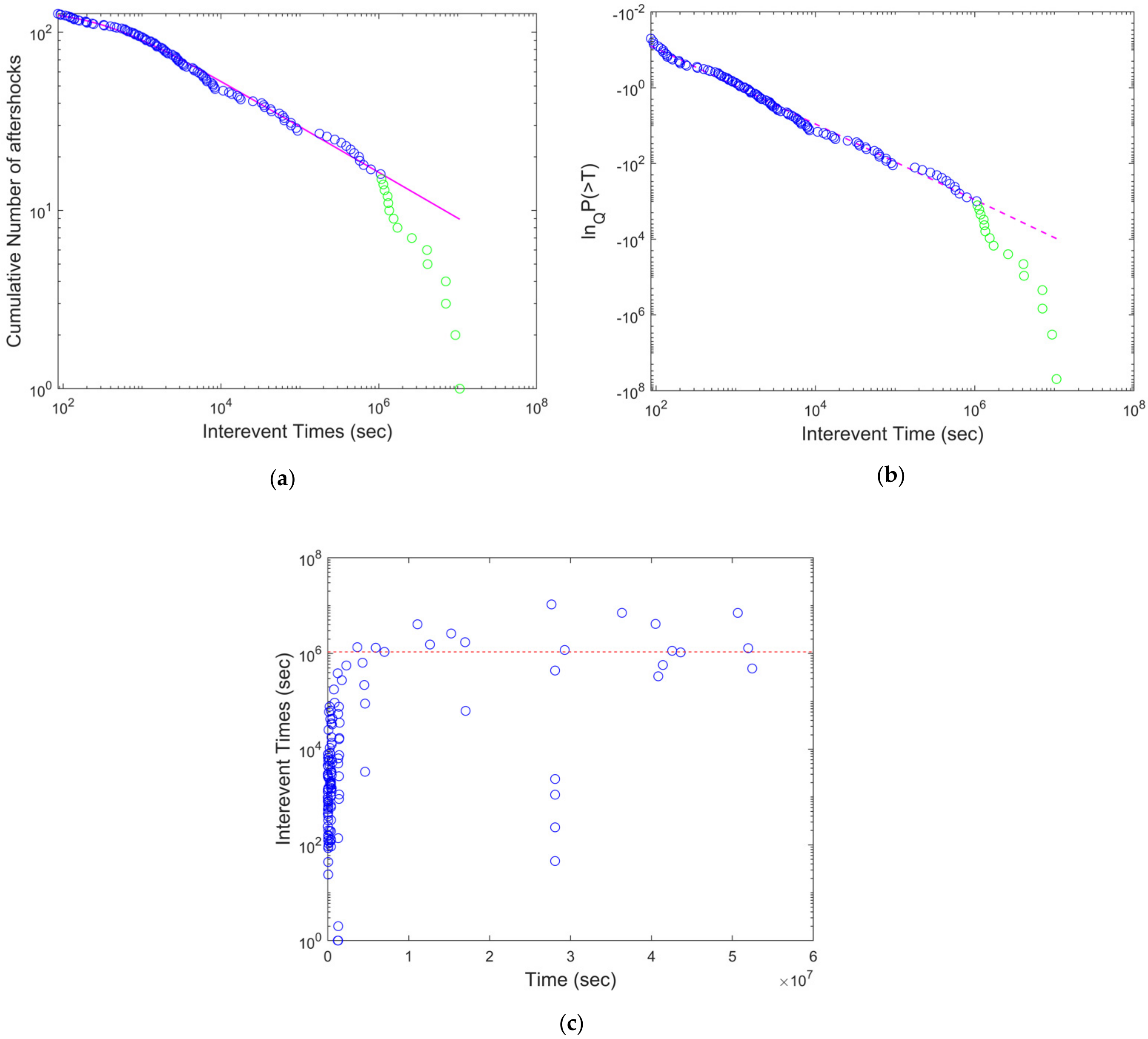

Figure S24. (**a**) The cumulative distribution function of the interevent times for the 2013 M_w 7.0 Atka Earthquake (Alaska). The magenta line is the Q-exponential function fitting with $q = 1.80$. (**b**) The Q-logarithmic function of *P(>T)* as a function of the interevent times, where the dashed line is the fitting with q = 1.80. The deviation from linearity suggests T_c values close to 1x10^6 s. The correlation coefficient for the Q-logarithmic function up to T_c is $R^2 = 0.9744$. (**c**) The evolution of the interevent time (*T*) as a function of the time (t) since the main event. The *T* value is indicated by the red dashed line.

25. The 2014 M_w 7.6 Kirakira Earthquake (Solomon Islands)

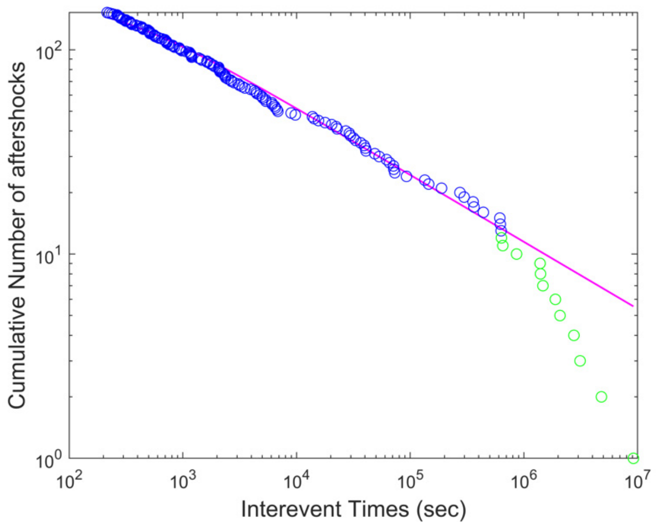

(**a**)

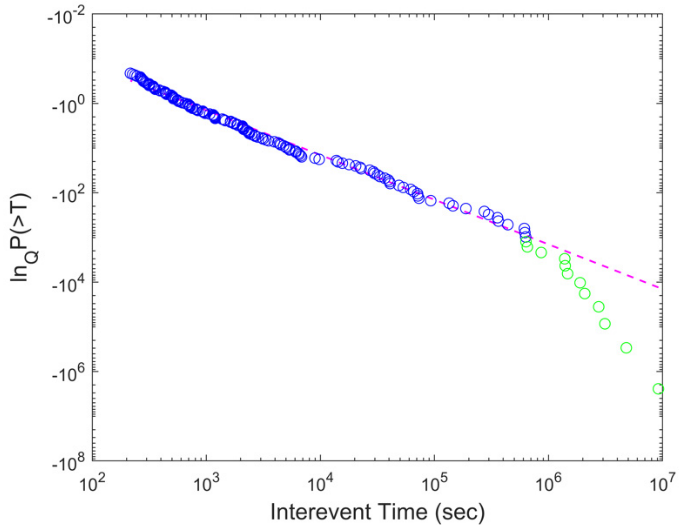

(**b**)

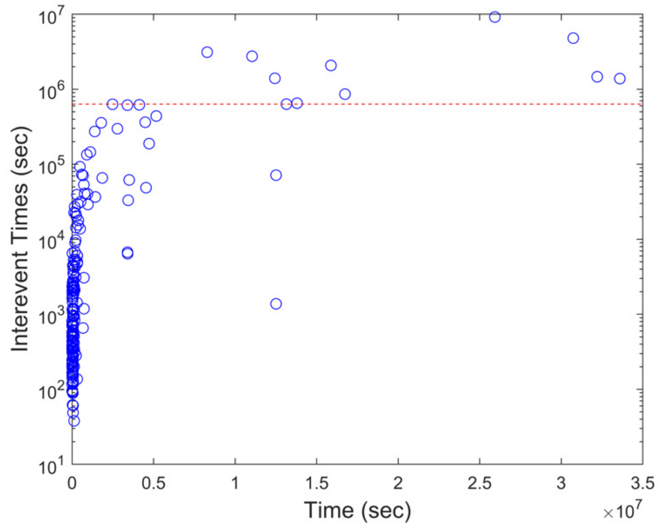

(**c**)

Figure S25. (**a**) The cumulative distribution function of the interevent times for the 2014 M_w 7.6 Kirakira Earthquake (Solomon Islands). The magenta line is the Q-exponential function fitting with q = 1.75. (**b**) The Q-logarithmic function of $P(>T)$ as a function of the interevent times, where the dashed line is the fitting with q = 1.75. The deviation from linearity suggests T_c values close to 6x10^6 s. The correlation coefficient for the Q-logarithmic function up to T_c is R^2 = 0.9711. (**c**) The evolution of the interevent time (T) as a function of the time (t) since the main event. The T value is indicated by the red dashed line.

26. The 2014 M_w 7.5 Panguna Earthquake (Papua New Guinea)

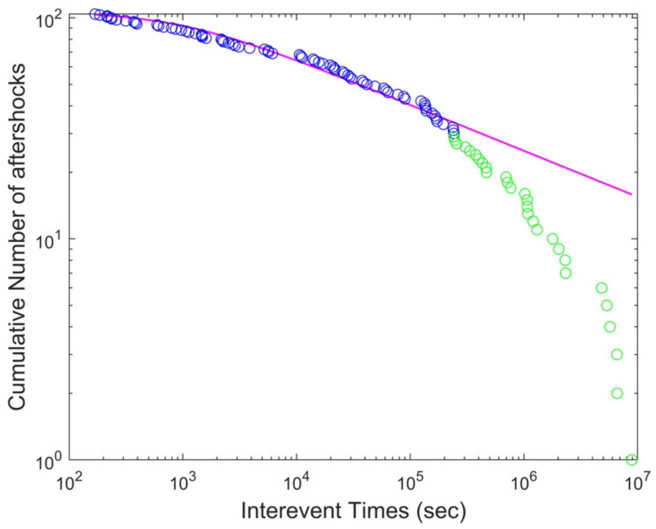

(**a**)

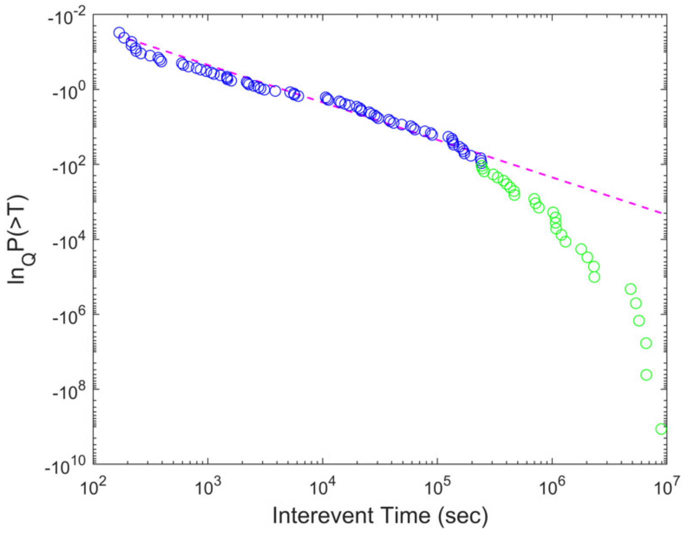

(**b**)

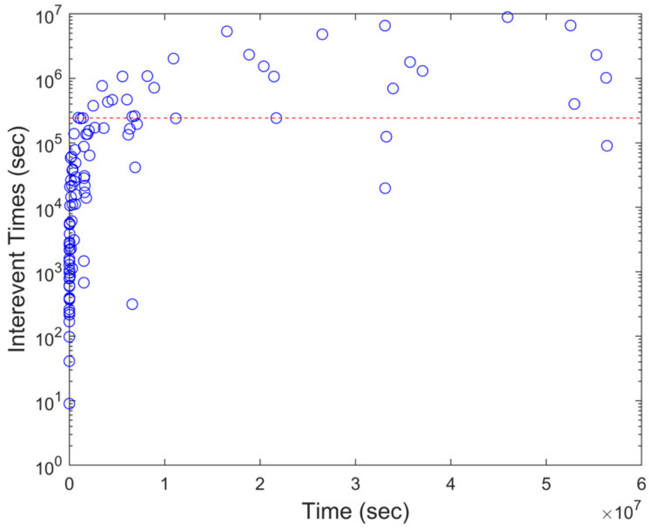

(**c**)

Figure S26. (**a**) The cumulative distribution function of the interevent times for the 2014 M_w 7.5 Panguna Earthquake (Papua New Guinea). The magenta line is the Q-exponential function fitting with q = 1.83. (**b**) The Q-logarithmic function of $P(>T)$ as a function of the interevent times, where the dashed line is the fitting with q = 1.83. The deviation from linearity suggests T_c values close to 2x10^5 s. The correlation coefficient for the Q-logarithmic function up to T_c is R^2 = 0.9173. (**c**) The evolution of the interevent time (T) as a function of the time (t) since the main event. The T value is indicated by the red dashed line.

27. The 2014 M_w 7.1 Ternate Earthquake (Indonesia)

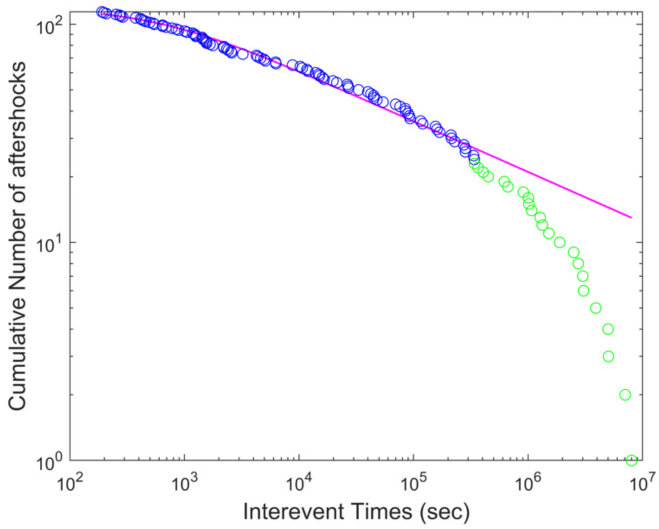

(**a**)

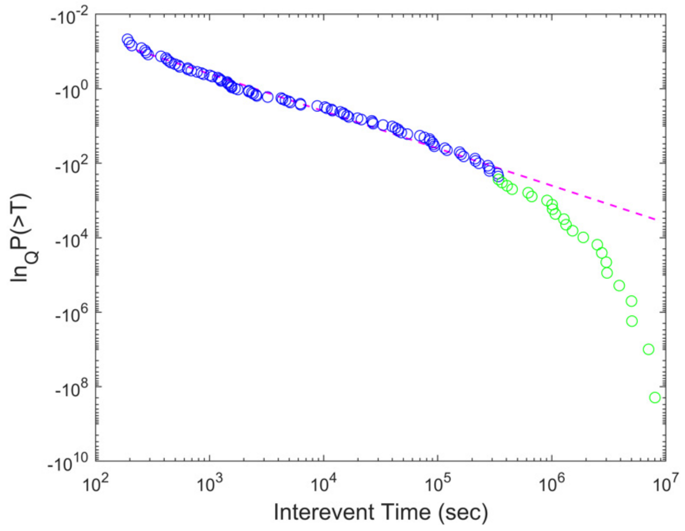

(**b**)

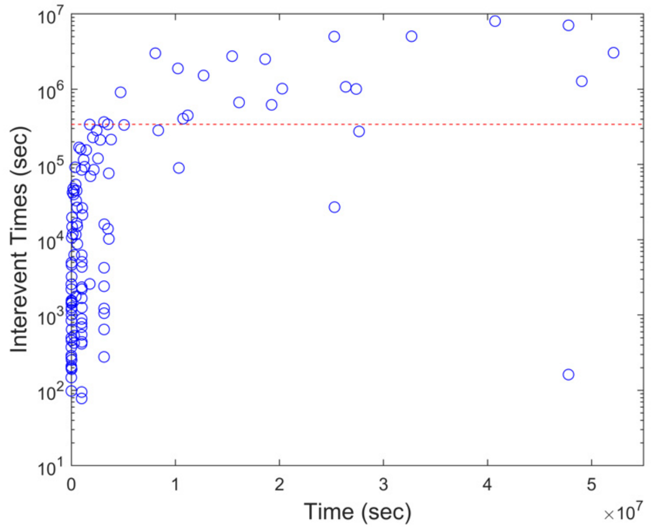

(**c**)

Figure S27. (**a**) The cumulative distribution function of the interevent times for the 2014 M_w 7.1 Ternate Earthquake (Indonesia). The magenta line is the Q-exponential function fitting with q = 1.81. (**b**) The Q-logarithmic function of $P(>T)$ as a function of the interevent times, where the dashed line is the fitting with q = 1.81. The deviation from linearity suggests T_c values close to 3x10^5 s. The correlation coefficient for the Q-logarithmic function up to T_c is R^2 = 0.9369. (**c**) The evolution of the interevent time (T) as a function of the time (t) since the main event. The T value is indicated by the red dashed line.

28. The 2015 M_w7.5 Kokopo Earthquake (Papua New Guinea)

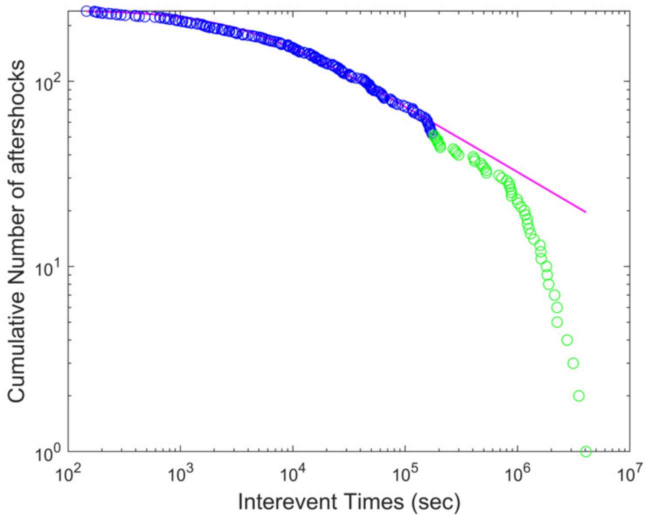

(**a**)

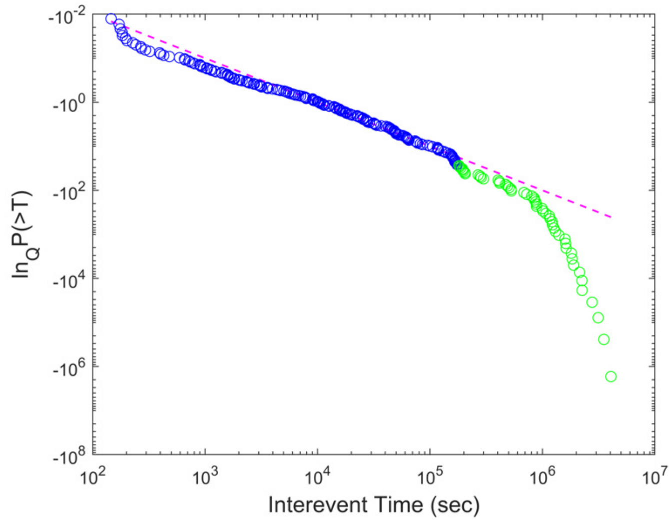

(**b**)

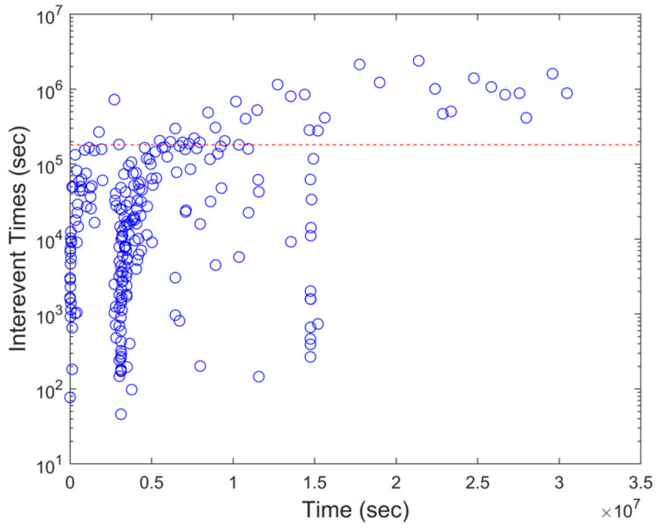

(**c**)

Figure S28. (**a**) The cumulative distribution function of the interevent times for the 2015 M_w7.5 Kokopo Earthquake (Papua New Guinea). The magenta line is the Q-exponential function fitting with q = 1.71. (**b**) The Q-logarithmic function of *P(>T)* as a function of the interevent times, where the dashed line is the fitting with q = 1.71. The deviation from linearity suggests T_c values close to 2x10^5 s. The correlation coefficient for the Q-logarithmic function up to T_c is R^2 = 0.9694. (**c**) The evolution of the interevent time (*T*) as a function of the time (t) since the main event. The *T* value is indicated by the red dashed line.

29. The 2015 M_w 8.3 Illapel (Chile) Earthquake

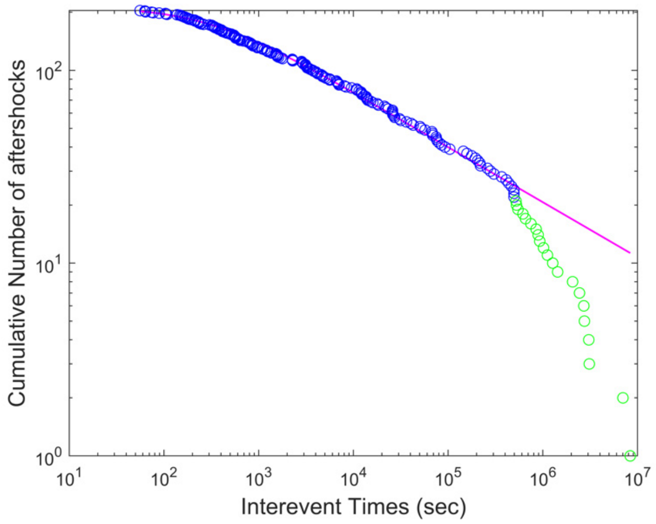

(**a**)

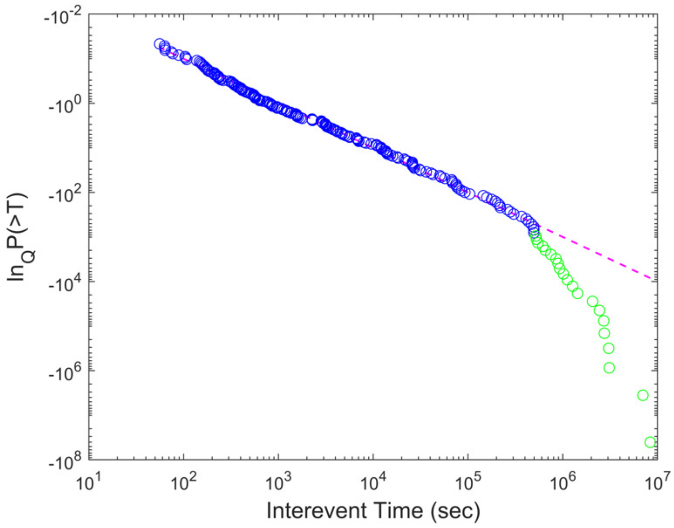

(**b**)

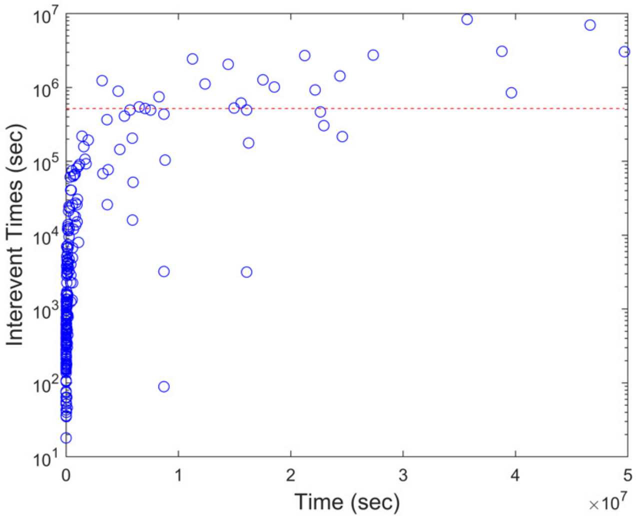

(**c**)

Figure S29. (**a**) The cumulative distribution function of the interevent times for the 2015 M_w 8.3 Illapel (Chile) Earthquake. The magenta line is the Q-exponential function fitting with $q = 1.78$. (**b**) The Q-logarithmic function of $P(>T)$ as a function of the interevent times, where the dashed line is the fitting with $q = 1.78$. The deviation from linearity suggests T_c values close to 6x10^5 s. The correlation coefficient for the Q-logarithmic function up to T_c is $R^2 = 0.9528$. (**c**) The evolution of the interevent time (T) as a function of the time (t) since the main event. The T value is indicated by the red dashed line.

30. The 2016 M_w 7.2 South Sandwich Islands Earthquake

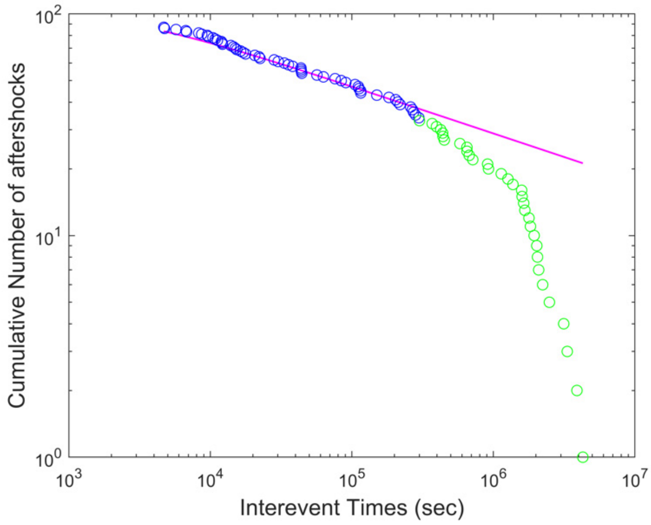

(**a**)

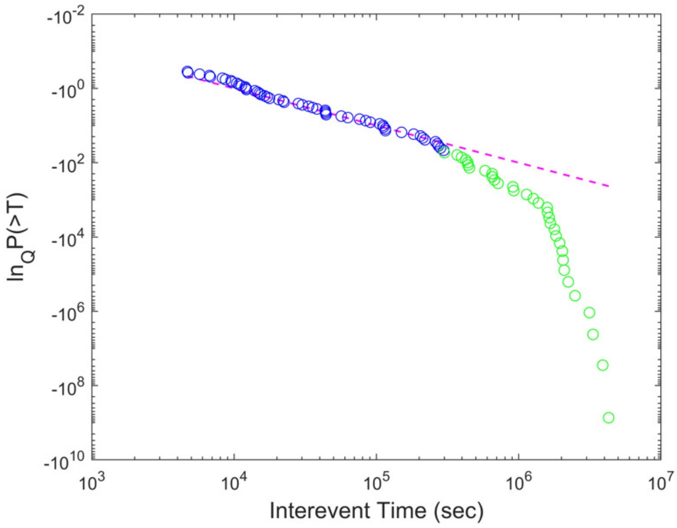

(**b**)

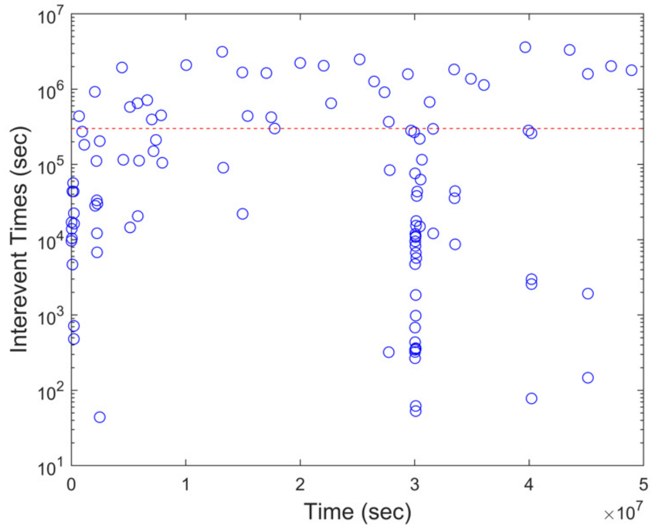

(**c**)

Figure S30. (**a**) The cumulative distribution function of the interevent times for the 2016 M_w 7.2 South Sandwich Islands Earthquake. The magenta line is the Q-exponential function fitting with q = 1.78. (**b**) The Q-logarithmic function of *P(>T)* as a function of the interevent times, where the dashed line is the fitting with q = 1.78. The deviation from linearity suggests T_c values close to 3x10^5 s. The correlation coefficient for the Q-logarithmic function up to T_c is R^2 = 0.9579. (**c**) The evolution of the interevent time (*T*) as a function of the time (t) since the main event. The *T* value is indicated by the red dashed line.

31. The 2016 M_w 7.0 Gisborne (New Zealand) Earthquake

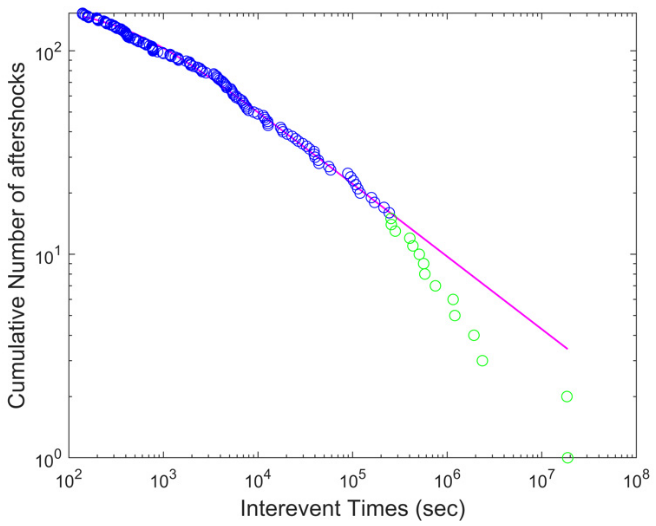

(**a**)

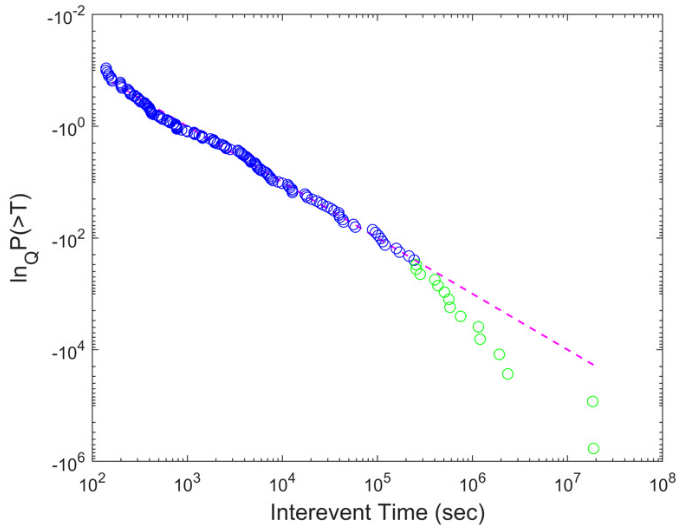

(**b**)

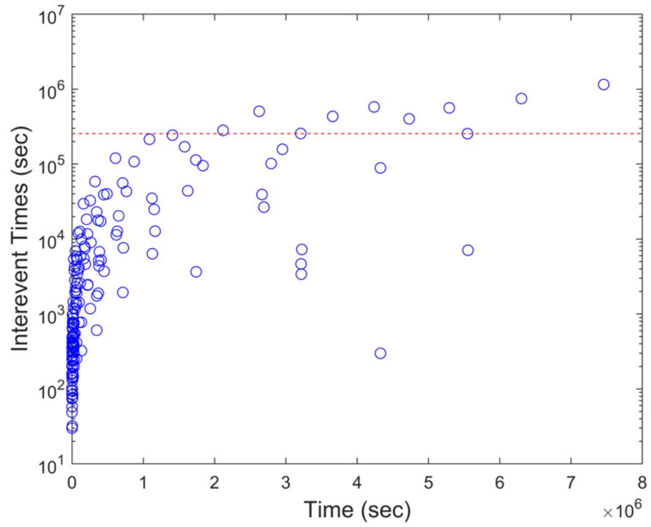

(**c**)

Figure S31. (**a**) The cumulative distribution function of the interevent times for the 2016 M_w 7.0 Gisborne (New Zealand) Earthquake. The magenta line is the Q-exponential function fitting with q = 1.74. (**b**) The Q-logarithmic function of $P(>T)$ as a function of the interevent times, where the dashed line is the fitting with q = 1.74. The deviation from linearity suggests T_c values close to 3x10^5 s. The correlation coefficient for the Q-logarithmic function up to T_c is R^2 = 0.9943. (**c**) The evolution of the interevent time (T) as a function of the time (t) since the main event. The T value is indicated by the red dashed line.

32. The 2016 M_w 7.8 Kirakira Earthquake (Solomon Islands)

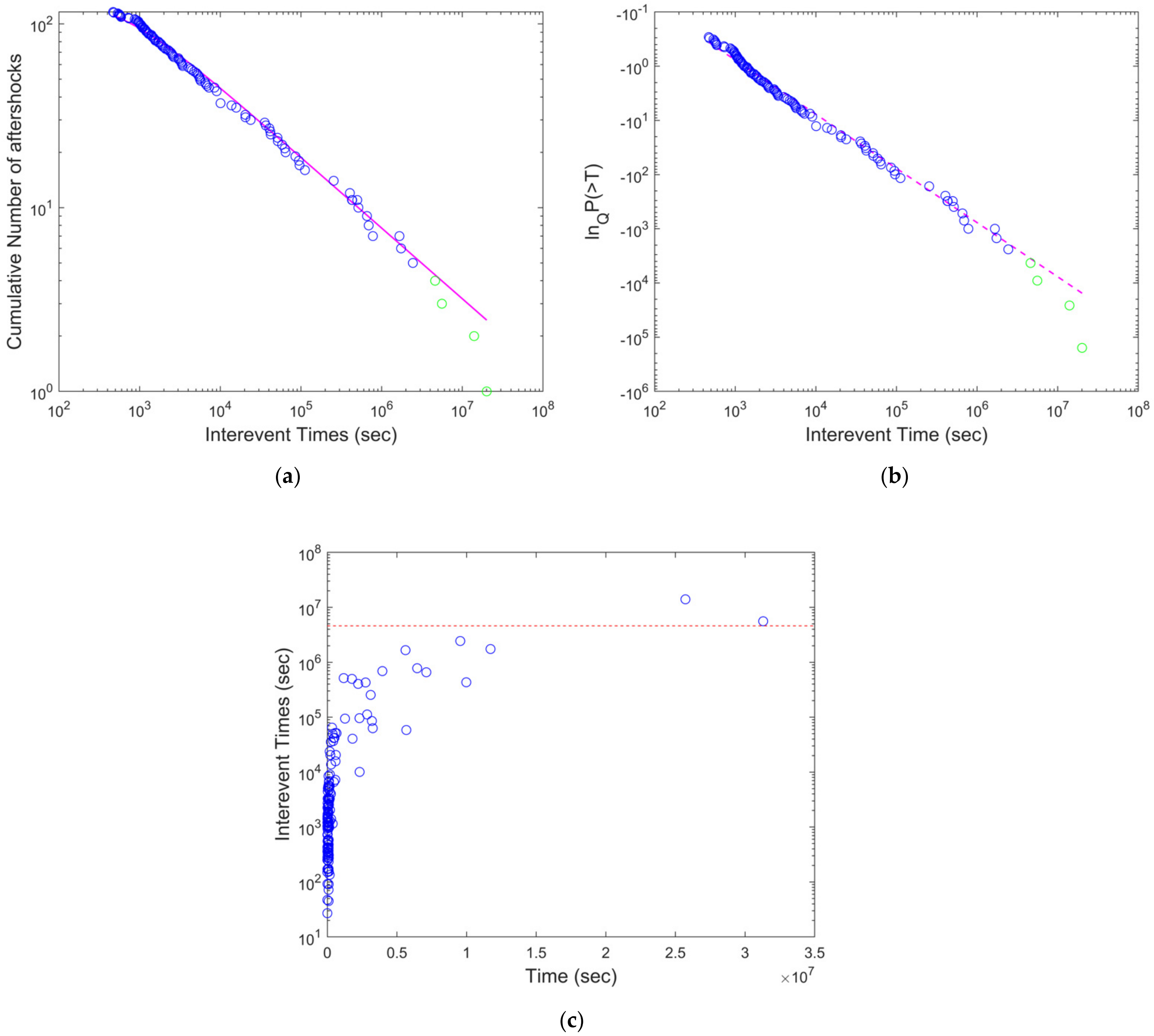

Figure S32. (**a**) The cumulative distribution function of the interevent times for the 2016 M_w 7.8 Kirakira Earthquake (Solomon Islands). The magenta line is the Q-exponential function fitting with q = 1.72. (**b**) The Q-logarithmic function of *P(>T)* as a function of the interevent times, where the dashed line is the fitting with q = 1.72. The deviation from linearity suggests T_c values close to 5x10^6 s. The correlation coefficient for the Q-logarithmic function up to T_c is R^2 = 0.9590. (**c**) The evolution of the interevent time (*T*) as a function of the time (t) since the main event. The *T* value is indicated by the red dashed line.

33. The 2016 M_w 7.8 Waiau (New Zealand)

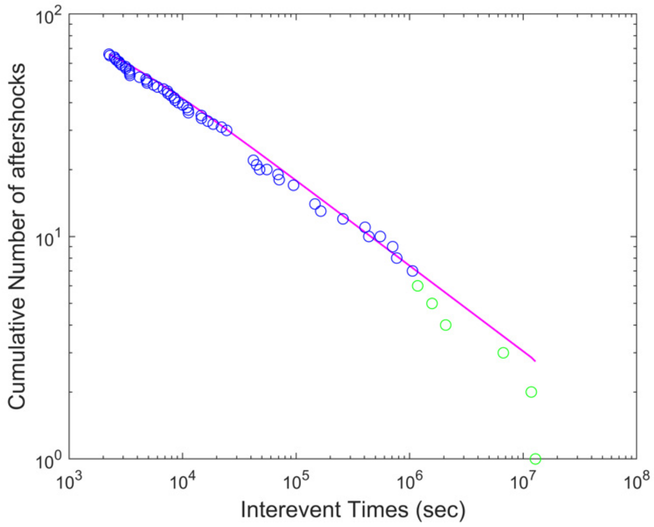

(**a**)

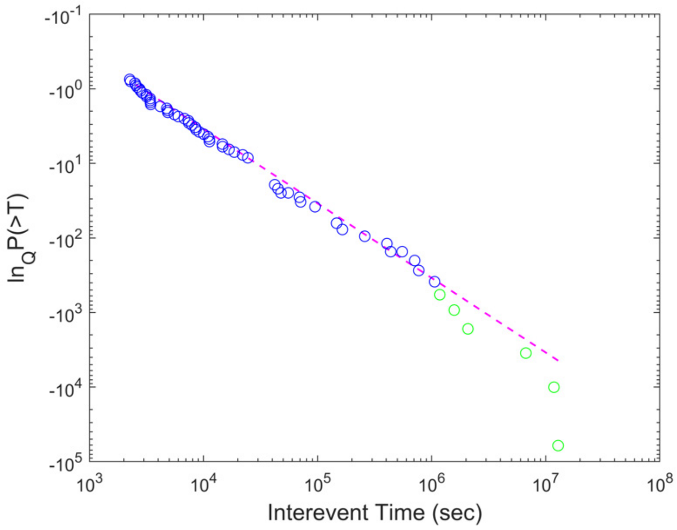

(**b**)

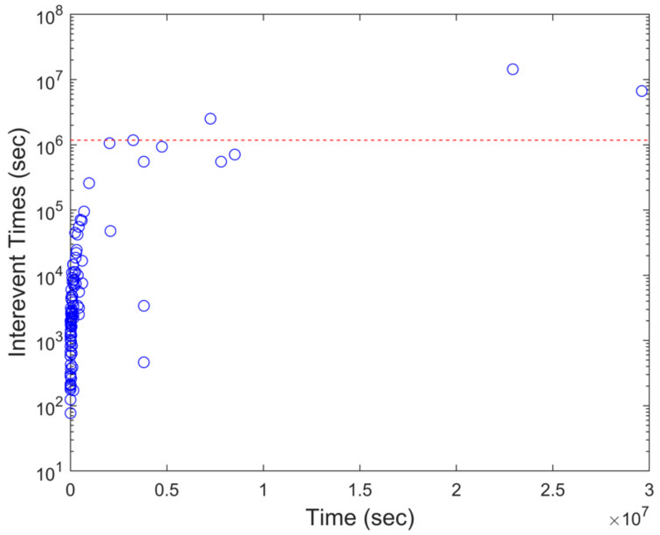

(**c**)

Figure S33. (**a**) The cumulative distribution function of the interevent times for the 2016 M_w 7.8 Waiau Earthquake (New Zealand). The magenta line is the Q-exponential function fitting with q = 1.72. (**b**) The Q-logarithmic function of $P(>T)$ as a function of the interevent times, where the dashed line is the fitting with q = 1.72. The deviation from linearity suggests T_c values close to $1\text{x}10^6$ s. The correlation coefficient for the Q-logarithmic function up to T_c is $R^2 = 0.9843$. (**c**) The evolution of the interevent time (T) as a function of the time (t) since the main event. The T value is indicated by the red dashed line.

34. The 2017 M_w 8.2 Chiapas (Mexico) Earthquake

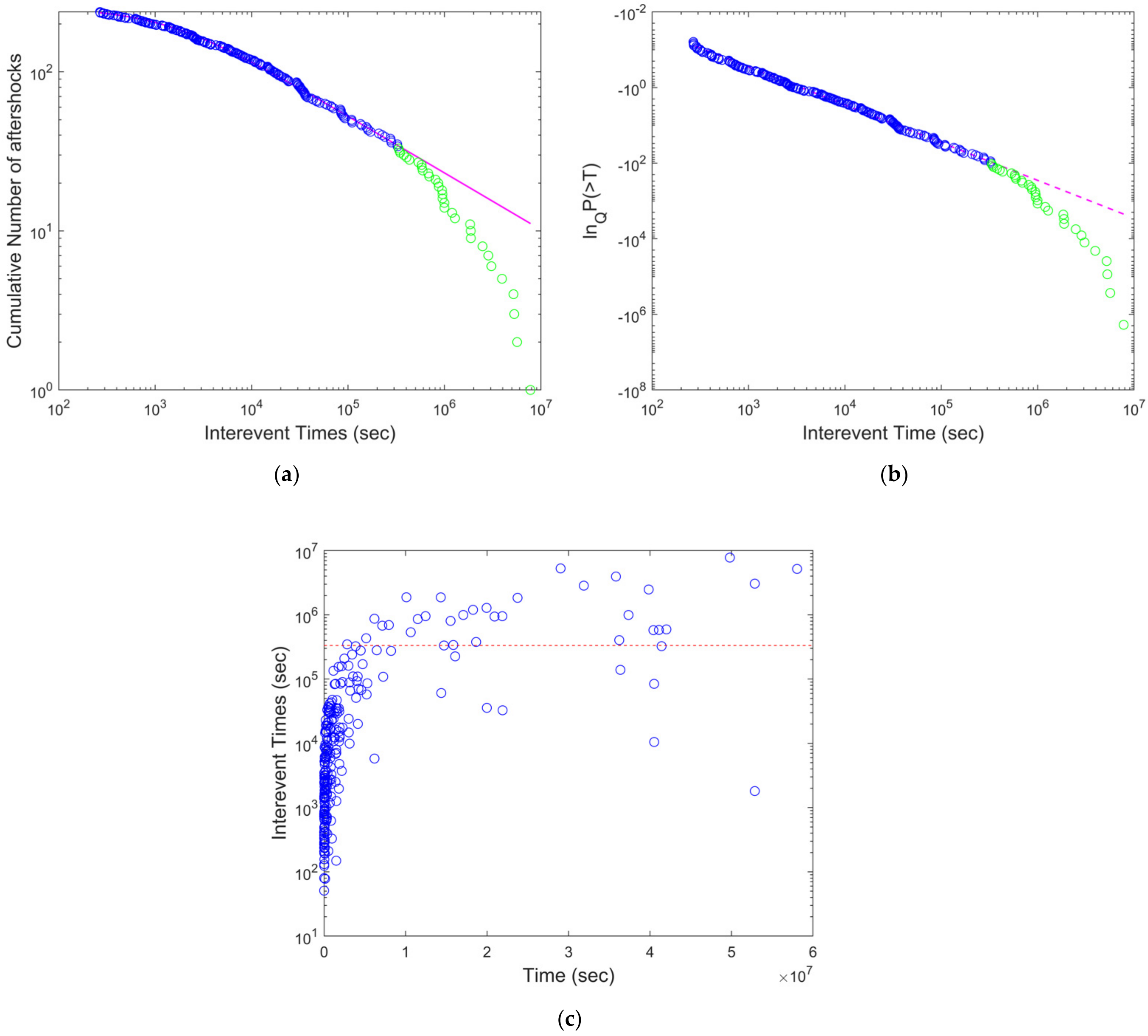

Figure S34. (**a**) The cumulative distribution function of the interevent times for the 2017 M_w 8.2 Chiapas (Mexico) Earthquake. The magenta line is the Q-exponential function fitting with $q = 1.74$. (**b**) The Q-logarithmic function of $P(>T)$ as a function of the interevent times, where the dashed line is the fitting with $q = 1.74$. The deviation from linearity suggests T_c values close to 3×10^5 s. The correlation coefficient for the Q-logarithmic function up to T_c is $R^2 = 0.9950$. (**c**) The evolution of the interevent time (T) as a function of the time (t) since the main event. The T value is indicated by the red dashed line.

35. The 2018 M_w 7.9 Chiniak Earthquake (Alaska)

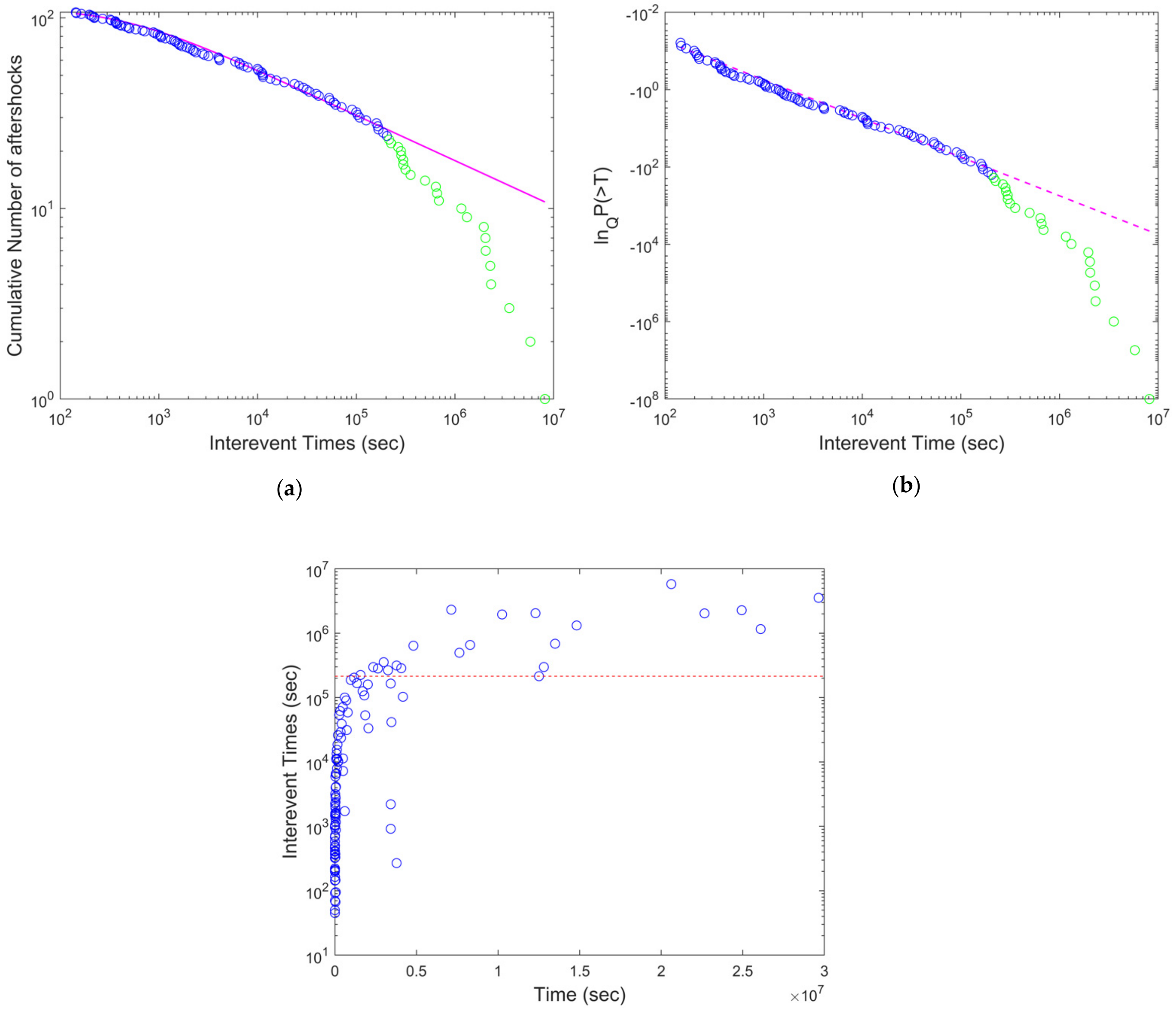

(**a**) (**b**) (**c**)

Figure S35. (**a**) The cumulative distribution function of the interevent times for the 2018 M_w 7.9 Chiniak Earthquake (Alaska). The magenta line is the Q-exponential function fitting with $q = 1.81$. (**b**) The Q-logarithmic function of *P(>T)* as a function of the interevent times, where the dashed line is the fitting with $q = 1.81$. The deviation from linearity suggests T_c values close to 2x10^5 s. The correlation coefficient for the Q-logarithmic function up to T_c is $R^2 = 0.9651$. (**c**) The evolution of the interevent time (*T*) as a function of the time (t) since the main event. The *T* value is indicated by the red dashed line.

36. The 2018 M_w 7.5 Tadine Earthquake (New Caledonia)

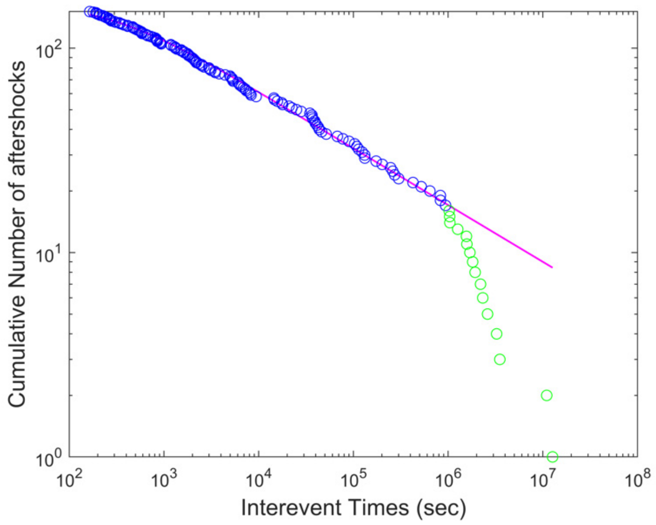

(**a**)

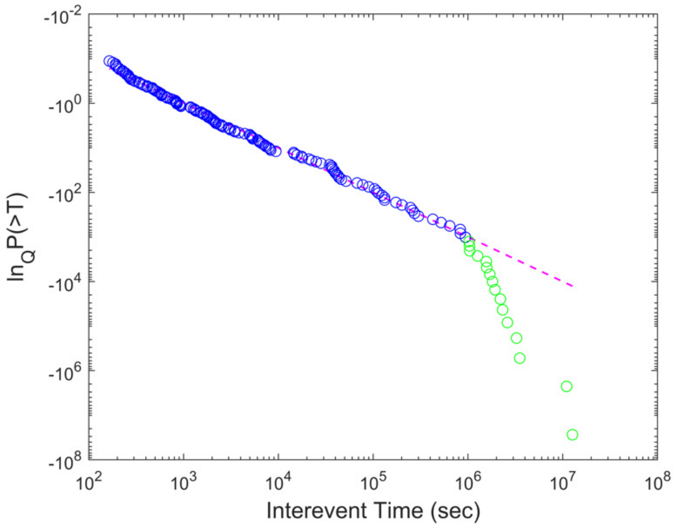

(**b**)

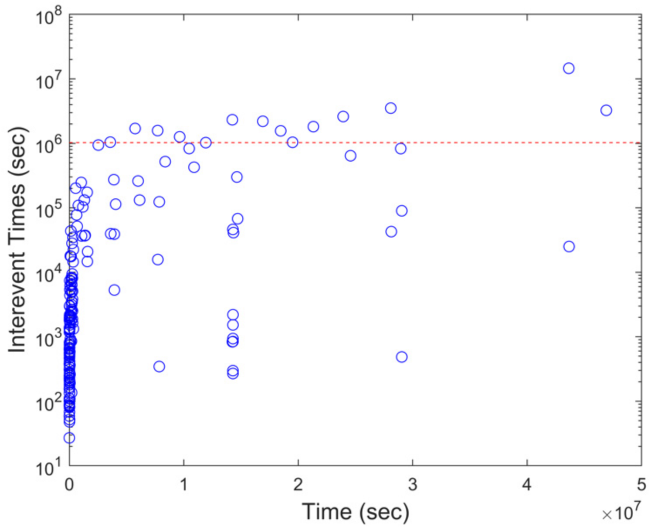

(**c**)

Figure S36. (**a**) The cumulative distribution function of the interevent times for the 2018 M_w 7.5 Tadine Earthquake (New Caledonia). The magenta line is the Q-exponential function fitting with q = 1.78. (**b**) The Q-logarithmic function of $P(>T)$ as a function of the interevent times, where the dashed line is the fitting with q = 1.78. The deviation from linearity suggests T_c values close to 1x10^5 s. The correlation coefficient for the Q-logarithmic function up to T_c is R^2 = 0.9882. (**c**) The evolution of the interevent time (T) as a function of the time (t) since the main event. The T value is indicated by the red dashed line.

37. The 2018 M_w 7.3 Uts′-Kamchatsk Staryy (Russia) Earthquake

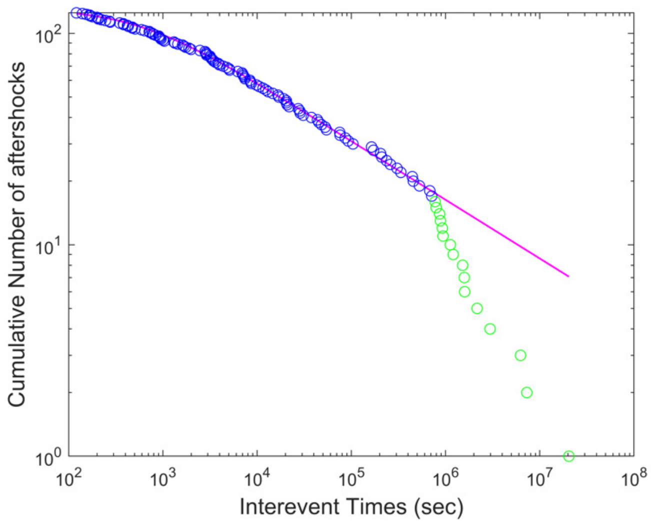

(**a**)

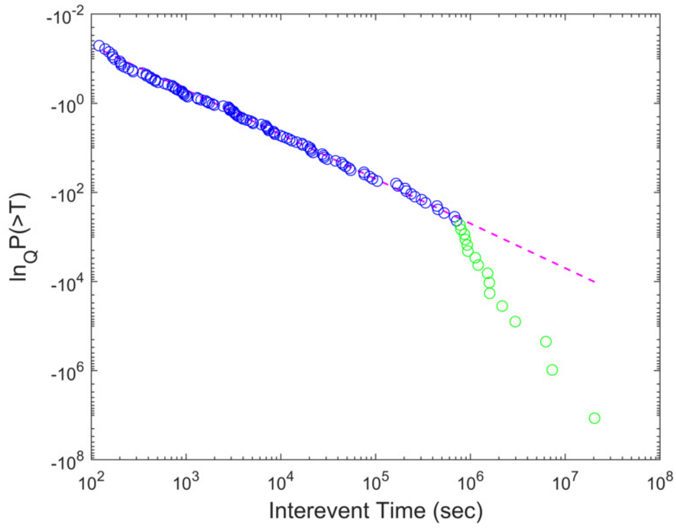

(**b**)

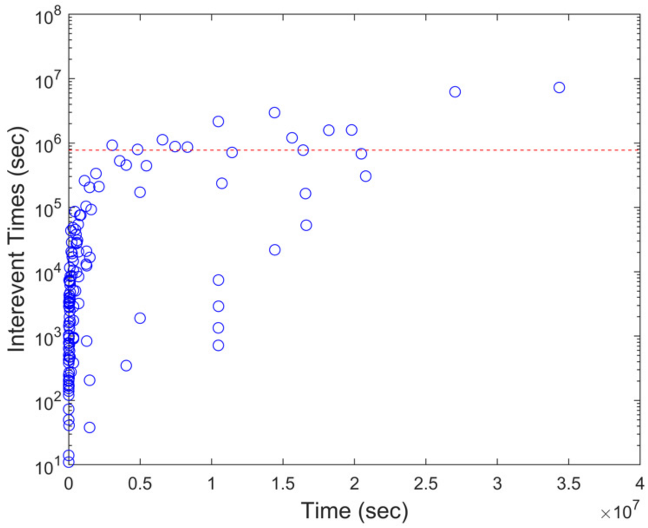

(**c**)

Figure S37. (**a**) The cumulative distribution function of the interevent times for the 2018 M_w 7.3 Uts′-Kamchatsk Staryy (Russia) Earthquake. The magenta line is the Q-exponential function fitting with q = 1.78. (**b**) The Q-logarithmic function of $P(>T)$ as a function of the interevent times, where the dashed line is the fitting with q = 1.78. The deviation from linearity suggests T_c values close to 8x10^5 s. The correlation coefficient for the Q-logarithmic function up to T_c is R^2 = 0.9870. (**c**) The evolution of the interevent time (T) as a function of the time (t) since the main event. The T value is indicated by the red dashed line.

38. The 2019 M_w 7.6 Kokopo Earthquake (Papua New Guinea)

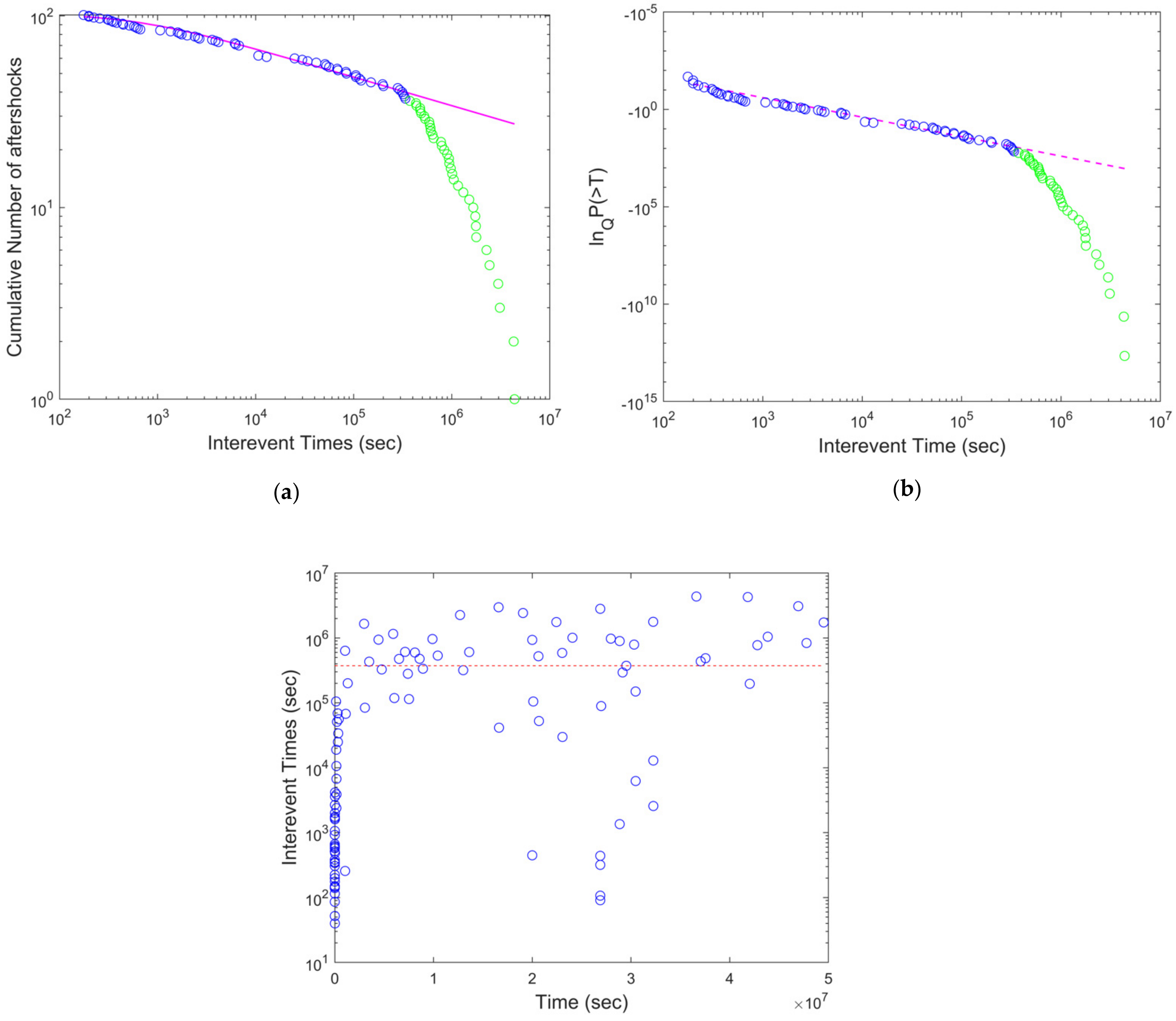

Figure S38. (**a**) The cumulative distribution function of the interevent times for the 2019 M_w 7.6 Kokopo Earthquake (Papua New Guinea). The magenta line is the Q-exponential function fitting with q = 1.86. (**b**) The Q-logarithmic function of *P(>T)* as a function of the interevent times, where the dashed line is the fitting with q = 1.86. The deviation from linearity suggests T_c values close to 4x10^5 s. The correlation coefficient for the Q-logarithmic function up to T_c is R^2 = 0.9327. (**c**) The evolution of the interevent time (*T*) as a function of the time (t) since the main event. The *T* value is indicated by the red dashed line.

39. The 2019 M_w 7.3 Kermadec Islands Earthquake (New Zealand)

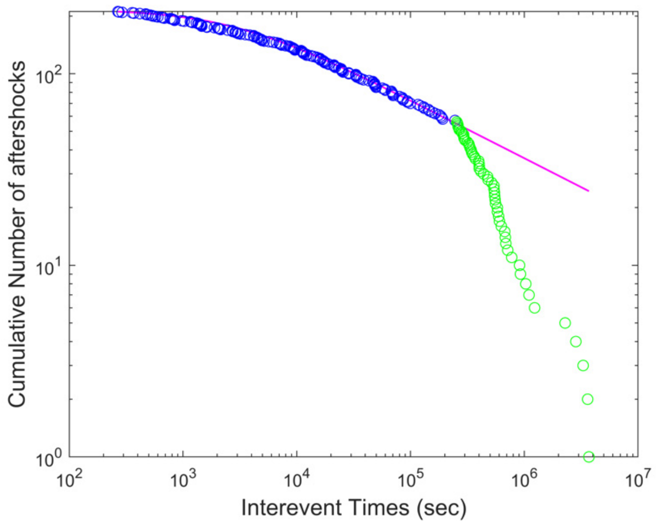

(**a**)

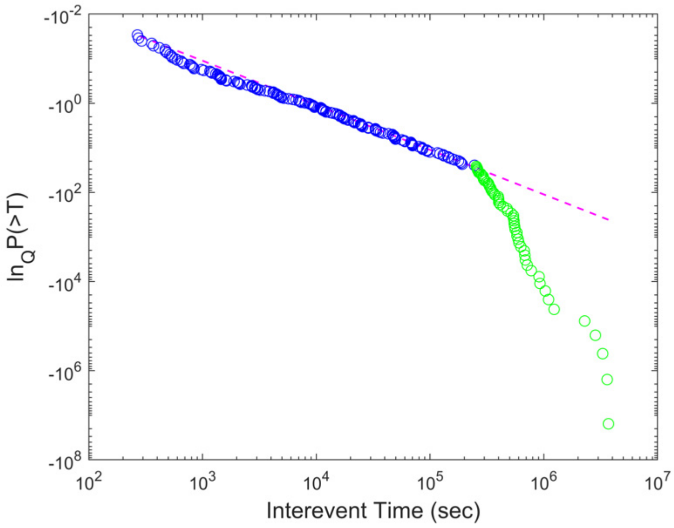

(**b**)

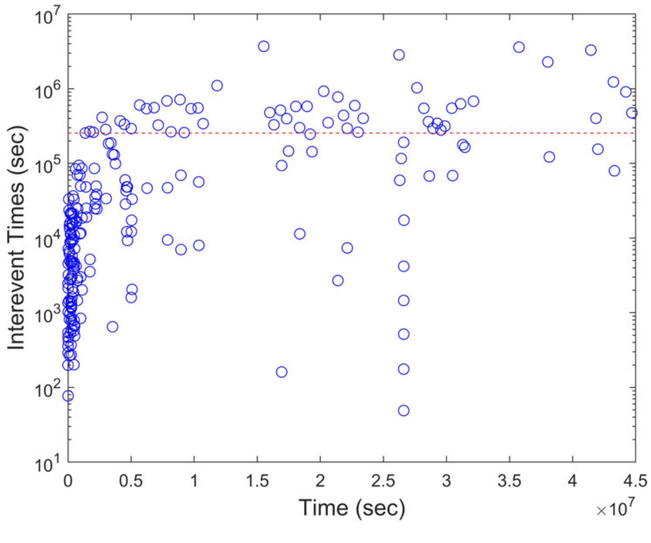

(**c**)

Figure S39. (**a**) The cumulative distribution function of the interevent times for the 2019 M_w 7.3 Kermadec Islands Earthquake (New Zealand). The magenta line is the Q-exponential function fitting with $q = 1.75$. (**b**) The Q-logarithmic function of $P(>T)$ as a function of the interevent times, where the dashed line is the fitting with $q = 1.75$. The deviation from linearity suggests T_c values close to 3×10^5 s. The correlation coefficient for the Q-logarithmic function up to T_c is $R^2 = 0.9953$. (**c**) The evolution of the interevent time (T) as a function of the time (t) since the main event. The T value is indicated by the red dashed line.

40. The 2019 M_w 7.1 Ternate (Indonesia) Earthquake

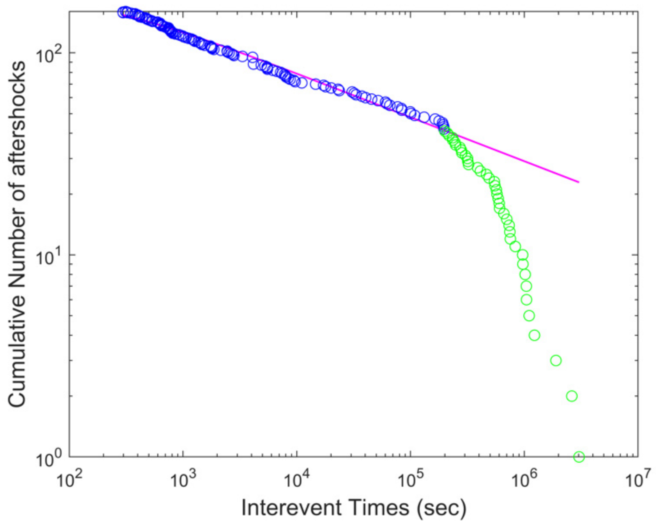

(**a**)

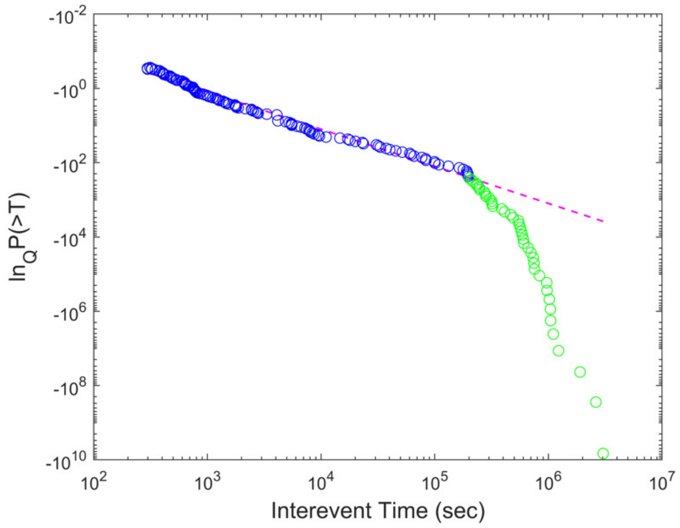

(**b**)

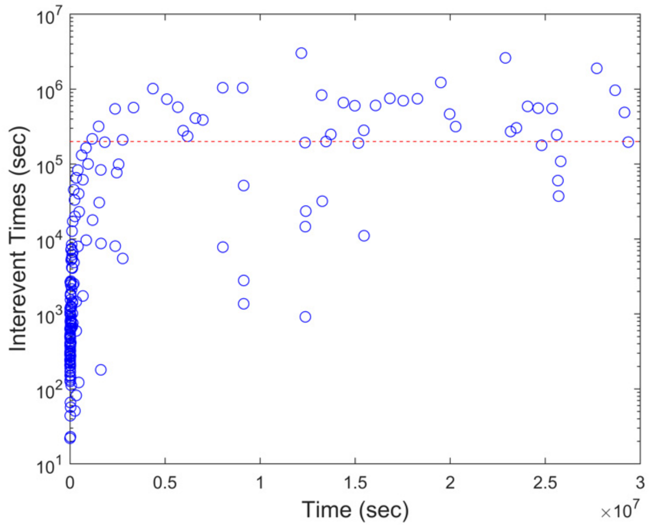

(**c**)

Figure S40. (**a**) The cumulative distribution function of the interevent times for the 2019 M_w 7.1 Ternate (Indonesia) Earthquake. The magenta line is the Q-exponential function fitting with q = 1.82. (**b**) The Q-logarithmic function of *P(>T)* as a function of the interevent times, where the dashed line is the fitting with q = 1.82. The deviation from linearity suggests T_c values close to 2x10^5 s. The correlation coefficient for the Q-logarithmic function up to T_c is R^2 = 0.9834. (**c**) The evolution of the interevent time (*T*) as a function of the time (t) since the main event. The *T* value is indicated by the red dashed line.

41. The 2020 M_w 7.4 Kermadec Islands Earthquake (New Zealand)

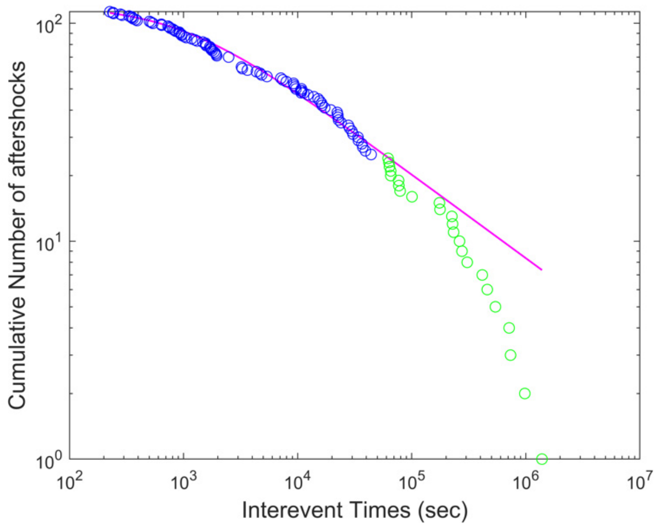

(**a**)

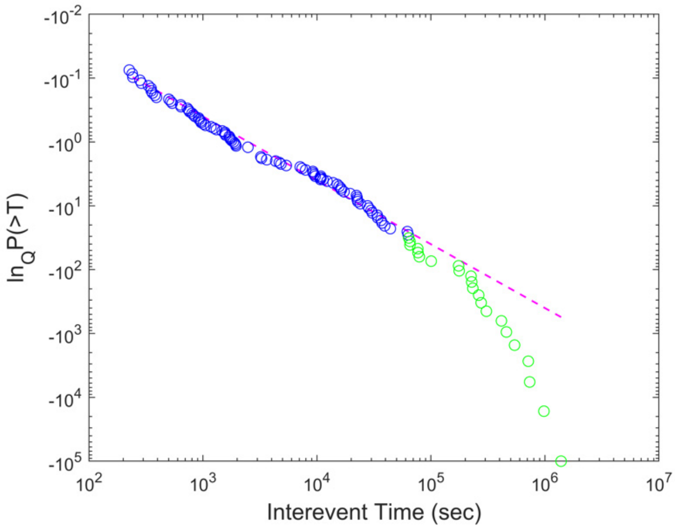

(**b**)

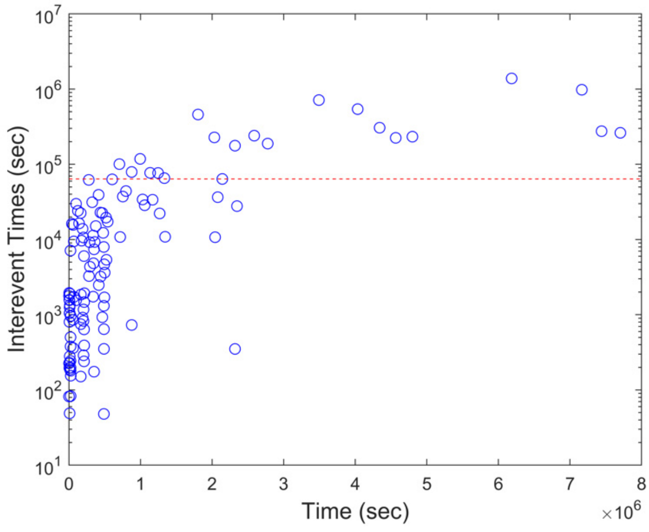

(**c**)

Figure S41. (**a**) The cumulative distribution function of the interevent times for the 2020 M_w 7.4 Kermadec Islands Earthquake (New Zealand). The magenta line is the Q-exponential function fitting with q = 1.72. (**b**) The Q-logarithmic function of $P(>T)$ as a function of the interevent times, where the dashed line is the fitting with q = 1.72. The deviation from linearity suggests T_c values close to 6×10^4 s. The correlation coefficient for the Q-logarithmic function up to T_c is R^2 = 0.9729. (**c**) The evolution of the interevent time (T) as a function of the time (t) since the main event. The T value is indicated by the red dashed line.

42. The 2020 M_w 7.8 Sand Point Earthquake (Alaska)

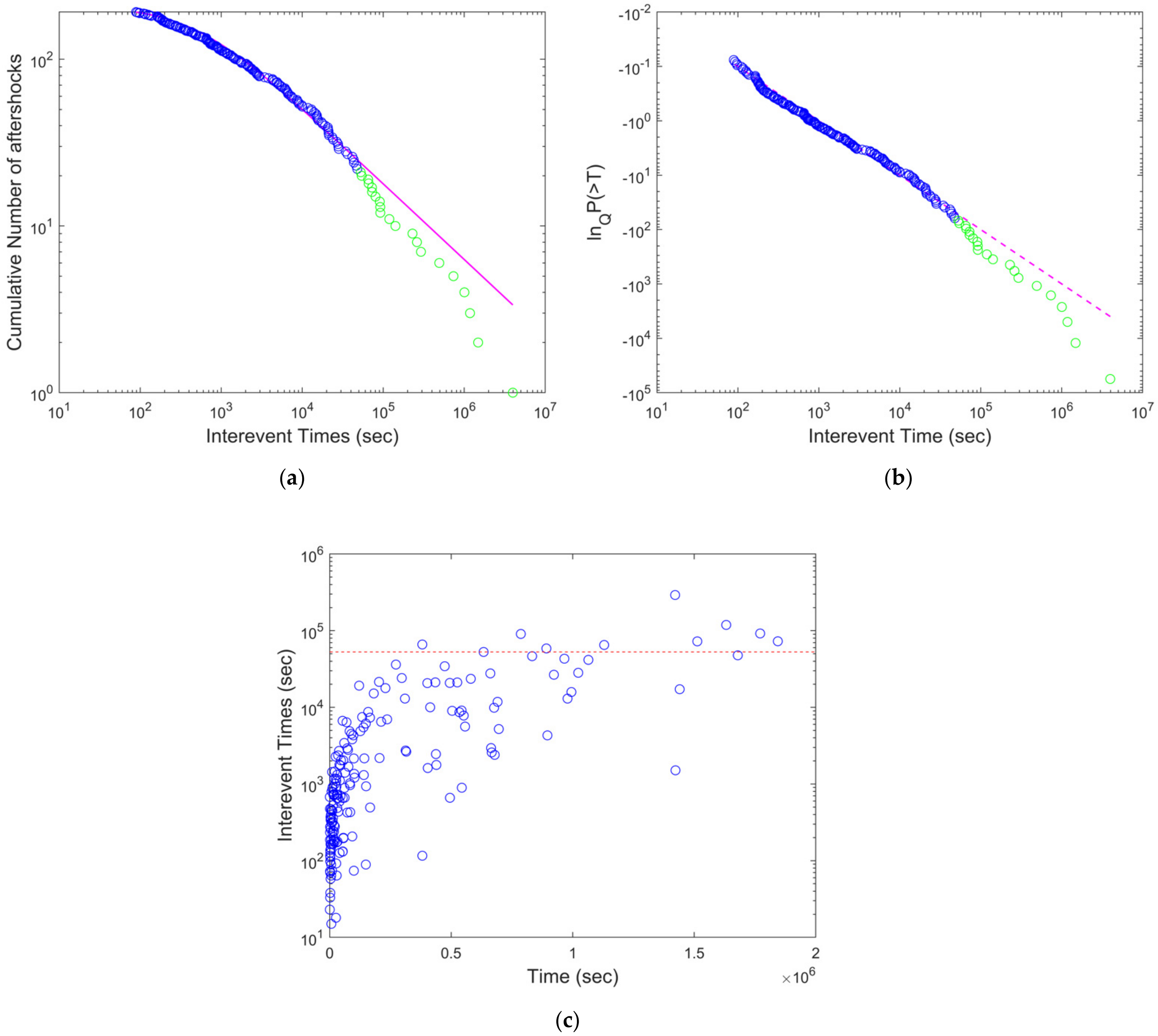

Figure S42. (**a**) The cumulative distribution function of the interevent times for the 2020 M_w 7.8 Sand Point Earthquake (Alaska). The magenta line is the Q-exponential function fitting with $q = 1.69$. (**b**) The Q-logarithmic function of $P(>T)$ as a function of the interevent times, where the dashed line is the fitting with $q = 1.69$. The deviation from linearity suggests T_c values close to 5×10^4 s. The correlation coefficient for the Q-logarithmic function up to T_c is $R^2 = 0.9775$. (**c**) The evolution of the interevent time (T) as a function of the time (t) since the main event. The T value is indicated by the red dashed line.
